# Study protocol for a multicentre, randomised, double-blinded, placebo-controlled, multi-arm, multi-stage, trial of SpironolacTone and famciclOovir in the treatment of Progressive Multiple Sclerosis to prevent disability progression: the STOP-MS trial

**DOI:** 10.1136/bmjno-2025-001313

**Published:** 2025-12-23

**Authors:** Kayla Ward, Vivien Li, Sudarshini Ramanathan, Lesley-Ann Hall, Katherine Buzzard, Kaylene Young, Fiona Mckay, Vanessa Vigar, Sabrina Oishi, Lidia Madrid San Martin, Belinda Kaskow, Grant Parnell, Julie A. Campbell, Jing Sun, Corey Smith, Vilija Jokubaitis, Tomas Kalincik, David Tscharke, Andrew Potter, Erin Brady, Jeannette Lechner-Scott, Lawrence Steinman, Mahesh Parmar, Jeremy Chataway, Todd Hardy, William M. Carroll, Michael H. Barnett, Bruce V. Taylor, Simon A. Broadley, Australian Multiple Sclerosis Clinical Trials Consortium, Suzanne Hodgkinson

**Affiliations:** 1 School of Medicine and Dentistry, Griffith University - Gold Coast Campus, Southport, Queensland, Australia; 2 Department of Neurology, Gold Coast University Hospital, Southport, Queensland, Australia; 3 The Florey Institute of Neuroscience and Mental Health, Parkville, Victoria, Australia; 4 Translational Neuroimmunology Group, Kids Neuroscience Centre and ANZAC Research Institute, The University of Sydney Sydney Medical School, Sydney, New South Wales, Australia; 5 Department of Neurology, Concord Hospital, Concord, New South Wales, Australia; 6 Flinders Medical Centre, Flinders University, Adelaide, South Australia, Australia; 7 Eastern Health Clinical School, Monash University, Box Hill, Victoria, Australia; 8 Menzies Institute for Medical Research, University of Tasmania, Hobart, Tasmania, Australia; 9 Multiple Sclerosis Australia, Multiple Sclerosis Australia, Summer Hill, New South Wales, Australia; 10 School of Medicine, Griffith University - Gold Coast Campus, Southport, Queensland, Australia; 11 National Centre for Naturopathic Medicine, Southern Cross University, Lismore, New South Wales, Australia; 12 Perron Institute for Neurological and Translational Science, Nedlands, Western Australia, Australia; 13 Centre for Immunology and Allergy Research, Westmead Institute for Medical Research, Westmead, New South Wales, Australia; 14 Infection, Immunity and Inflammation Group, School of Medical Sciences, The University of Sydney, Sydney, New South Wales, Australia; 15 University of Tasmania Menzies Institute for Medical Research, Hobart, Tasmania, Australia; 16 Rural Health Research Institute, Charles Sturt University, Albury, New South Wales, Australia; 17 QIMR Berghofer Medical Research Institute, Herston, Queensland, Australia; 18 Department of Neuroscience, Monash University Central Clinical School, Melbourne, Victoria, Australia; 19 CORe, The University of Melbourne Department of Medicine RMH, Parkville, Victoria, Australia; 20 Department of Neurology, The Royal Melbourne Hospital, Melbourne, Victoria, Australia; 21 Immunology and Infectious Diseases, Australian National University The John Curtin School of Medical Research, Canberra, Australian Capital Territory, Australia; 22 Multiple Sclerosis Australia, Summer Hill, New South Wales, Australia; 23 Department of Neurology, John Hunter Hospital, New Lambton Heights, New South Wales, Australia; 24 The University of Newcastle Hunter Medical Research Institute, New Lambton, New South Wales, Australia; 25 Departments of Neurology and Neurological Sciences, Stanford University, Stanford, California, USA; 26 ACORD at MRC Clinical Trials Unit, University College London Institute of Clinical Trials and Methodology, London, UK; 27 NIHR University College London Hospitals Biomedical Research Centre, London, UK; 28 Queen Square Multiple Sclerosis Centre, Department of Inflammation, UCL Queen Square Institute of Neurology, London, UK; 29 Department of Neurology, Concord Repatriation General Hospital, Concord, New South Wales, Australia; 30 The University of Sydney Brain and Mind Centre, Camperdown, New South Wales, Australia; 31 Department of Neurology, Sir Charles Gairdner Hospital, Nedlands, Western Australia, Australia; 32 The University of Sydney Sydney Medical School, Sydney, New South Wales, Australia

**Keywords:** MULTIPLE SCLEROSIS, RANDOMISED TRIALS, VIROLOGY

## Abstract

**Introduction:**

Targeting progressive multiple sclerosis (MS) addresses the current single biggest unmet need in the MS therapeutic landscape and anti-Epstein-Barr virus (EBV) therapy potentially strikes at the root cause. The SpironolacTone and famciclOvir in the treatment of Progressive MS to prevent disability progression (STOP-MS) trial has been developed to assess anti-EBV therapies in the treatment of progressive MS.

**Methods and analysis:**

STOP-MS is a multi-arm, multi-stage, randomised, double-blind, placebo-controlled trial testing spironolactone and famciclovir to prevent disability progression in MS. Australians with progressive forms of MS, aged 25 to 70 years with established disability, are eligible. Recruitment commenced in March 2025 and the first participant was enrolled on 15 April 2025. The sample size for STOP-MS is 150 in stage 1 and 300 in stage 2. In stage 1, the composite primary outcome measures will be reduction of EBV DNA in saliva and serum EBV nuclear antigen-1 antibody titres. Minimum criteria for consideration of progression to stage 2 will be a 10% reduction in the composite outcome measure. In stage 2, the primary outcome measure will be 6-month confirmed disability progression analysed using Cox-proportional hazards.

**Trial registration number:**

The STOP-MS trial has been acknowledged by the Therapeutics Goods Administration under the Clinical Trial Notification scheme (CT-2023-CTN-03 505-1) and is registered with the Australian and New Zealand Clinical Trial Registry (ACTRN12623000849695).

**WHAT IS ALREADY KNOWN ON THIS TOPIC**

- Epstein-Barr virus (EBV) is a primary driver of multiple sclerosis (MS) pathology and may contribute significantly to disease progression. There are existing drugs with evidence for anti-EBV effects which may be repurposed for this indication.

**WHAT THIS STUDY ADDS**

- People with progressive MS do not have time to await development of new therapeutics to slow progression. STOP-MS has the potential to rapidly deliver evidence for effective anti-EBV therapies for this group.

**HOW THIS STUDY MIGHT AFFECT RESEARCH PRACTICE OR POLICY**

- A positive result from the STOP-MS trial could be rapidly translated to clinical practice through applications for additional indications to regulatory authorities for these already approved therapies and through publication of clinical guidelines.

## Introduction

 Multiple sclerosis (MS) is a complex autoimmune and neurodegenerative condition that can manifest clinically with both discrete neurological events (relapses) and gradual worsening of disability (progression).^1^ The most common form of MS is relapsing remitting (90% at disease onset), where recurrent bouts of symptoms punctuate periods of relative clinical normality.^2^ After 10–30 years, without treatment, 60% of these cases will transition to secondary progressive MS, where disability continues to accrue independent of relapse activity.^3^ In around 10% of cases, the disease is progressive from the outset, which is termed primary progressive MS.^4^ These latter two forms have been collectively termed progressive MS, although current concepts of MS now recognise this as a continuum of the same disease process as relapsing forms of MS.^5^ Progressive MS currently affects over 20 000 Australians and 1.4 million individuals globally.^6^ Highly effective disease-modifying therapies (DMT) dramatically reduce the risk of relapse associated worsening but have limited impact on progression independent of relapse activity.^7^ Over 40 traditional phase II and phase III clinical trials evaluating putative therapies for progressive MS in the last three decades have yielded mostly negative or mixed results, or been constrained by the toxicity and side-effects of the tested drugs.^8^ Existing therapies for relapsing MS have demonstrated benefit for progressive forms of MS, but this effect may be mediated by their impact on relapse-related pathology rather than neuroprotective effects. A recent Cochrane network meta-analysis could not be confident of any benefit for these agents in progressive MS^9^ and they have not been approved for use in progressive MS in most jurisdictions. The Bruton tyrosine kinase inhibitors have been postulated to be effective for progressive disease biology and hold some promise for people with progressive MS, but come with additional risks.^10^ There remains an urgent need for an innovative and rapid new approach to identify and repurpose existing medications which are identified as efficacious and safe to treat progressive MS.

MS is known to arise as the result of a combination of genetic and environmental factors. Certain genes including human leucocyte antigen-DRB1*15:01 and over 200 other genetic loci are known to increase the risk of MS.^11^ Relative vitamin D deficiency, lack of sunlight prior to age 15, smoking, obesity and a diet high in saturated fat are all environmental factors that increase the risk of MS.^12^ A history of infectious mononucleosis (IM) is more common in people with MS than controls^13^ and MS risk is associated with IM at a later age.^14^ Evidence of infection with Epstein-Barr virus (EBV), the most common cause of IM, is essentially universal in people with MS, compared with being seen in 90% of the general adult population.^15^ Recent studies suggest that MS only occurs in people who acquire EBV prior to the onset of their disease.^16^ There is a near-linear relationship between age of IM and age of onset of MS with a latency of 10–20 years.^17^ Acute EBV infection often manifests as IM when this occurs in older children and adults, but may be asymptomatic or minimally symptomatic particularly in younger children. EBV is a herpes virus that following acute infection enters a latent phase within B cells and persists indefinitely, evading immune surveillance.^18^ There is evidence that autoreactive, EBV-infected, B cells sequestered in lymphoid tissue within the central nervous system (CNS), in particular the pial surface of the grey matter, are a primary driver of continuing low-grade inflammation which leads to progressive disability in the absence of relapses in MS.^19^ Once EBV has infected B cells, the virus goes through a cycle of phases but ultimately enters a latent state where specific antigens, including Epstein-Barr virus nuclear antigen-1 (EBNA1), are produced.^20^ It is known that people with MS have higher levels of antibodies to EBNA1, indicating that T cell responses (the normal mechanism for clearance of intracellular pathogens) may be dysfunctional in people with MS.^21^ EBNA1 antibodies are also known to cross-react with specific regions of glial cell adhesion molecule,^22^ anoctamin 2,^23^ alpha-crystallin B chain^24^ and myelin,^25^ potentially leading to the generation of autoantibodies directly against these components of the CNS in people with MS. These lines of evidence have resulted in the conclusion that EBV is ‘essential, but not sufficient’, to cause MS and that EBV may be the primary driver of MS pathology and disease activity.

Many existing therapies for MS can be postulated to work by either removing EBV-infected B cells (ocrelizumab, ofatumumab, rituximab, alemtuzumab, teriflunomide, cladribine), blocking their entry into the CNS (natalizumab), trapping them in lymphoid tissue (fingolimod, siponimod, ozanimod, ponesimod), or through upregulating anti-viral responses via γ-receptors (β-interferons).^26^ Recent studies of teriflunomide have demonstrated significant reductions in salivary EBV DNA and EBNA1 antibody titres in people with MS.^27^ Importantly, this study demonstrated that this effect was seen within 3–6 months of commencing teriflunomide. It is therefore logical to consider existing therapies with known efficacy against EBV as potential treatments for delaying or halting progression of MS. Several existing anti-viral and other agents are known to have in vitro and clinical activity against EBV.^28^ A key element to any putative therapy in this population is CNS penetration. The blood-brain barrier is a multi-layered filtering system that normally protects the brain from the passage of large molecules and immune cells.^29^ For efficacy against the pial lymphoid tissue, high CNS and blood-brain barrier penetrance would be desirable. A peer-reviewed process that involved consumers was undertaken for assessing putative anti-EBV therapies focusing on safety, CNS penetrance and evidence for efficacy against both the lytic and latent phases of the EBV life cycle.^30^ On this basis, spironolactone and famciclovir were selected as the investigational medicinal products (IMP) for STOP-MS from a final shortlist of four agents. The cost of maribavir was prohibitive and safety data pertaining to tenofovir excluded its use in a potentially older population.

Spironolactone inhibits EBV replication in the late lytic phase by blocking SM protein.^31^ In a pilot study of 9 people with MS (6 with progressive MS), a combination of spironolactone and aldosterone was effective in improving symptoms.^32^ Famciclovir is a prodrug of penciclovir which inhibits EBV replication in cell culture.^33^ Randomised, double-blind, placebo-controlled trials of valaciclovir (the prodrug of aciclovir) have shown a reduction in the number of new active lesions on MRI in those with active disease, and trends towards a reduction in annualised relapse rate and Expanded Disability Status Scale (EDSS) progression.^34^ Famciclovir was chosen over aciclovir and valaciclovir because of its greater bioavailability, higher intracellular concentration and greater persistence in infected cells.^35^ A more recent trial looking at salivary shedding of EBV DNA in people with MS taking famciclovir did not see any reduction in shedding rates.^36^ However, this was a small study and the treatment period was only 12 weeks. The active agents will be compared against placebo in order to demonstrate efficacy. Existing standard of care (SOC) treatments for MS will be permitted in all arms of the trial. This was a strong recommendation from our consumer advisory group.

### Objectives and hypotheses

To assess the efficacy of these repurposed therapies with potential efficacy against EBV, a multicentre, randomised, double-blind, placebo-controlled, multi-arm, multi-stage (MAMS) trial of SpironolacTone and famciclOvir in the treatment of Progressive MS to prevent disability progression (STOP-MS) is proposed. This multi-stage trial will initially assess efficacy of the two agents against placebo, evaluating laboratory measures of EBV activity (salivary EBV DNA shedding and EBNA1 titres) in stage 1 and standard clinical measures, time to 6-month confirmed disability progression (6mCDP), in stage 2, with only successful treatments from stage 1 progressing to stage 2. The primary aim of stage 1 of this trial will be to demonstrate that spironolactone or famciclovir plus SOC reduce the frequency of EBV DNA being present in saliva and/or reduce EBNA1 antibody titres in people with progressive MS when compared with placebo plus SOC over 6 months. The primary aim of stage 2 will be to demonstrate that spironolactone or famciclovir plus SOC reduce the likelihood of 6mCDP in people with progressive MS when compared with placebo plus SOC over 3 years. The secondary aims will be to demonstrate that spironolactone or famciclovir plus SOC are safe and cost-effective when used to treat people with progressive MS, there is improvement in patient-reported outcome measures (PROMs) and there is radiological evidence of reduced brain atrophy and reduced occurrence of new/expanding or gadolinium (Gd)-enhancing lesions compared with placebo plus SOC.

STOP-MS will test the hypothesis that spironolactone or famciclovir plus SOC will reduce the frequency of salivary EBV shedding or reduce EBNA1 antibody titres in people with progressive MS when compared with placebo plus SOC in stage 1. The most effective therapy, provided minimum requirements for efficacy are met, will then be tested in stage 2 with the hypothesis that spironolactone or famciclovir plus SOC will reduce the likelihood of 6mCDP when compared with placebo plus SOC.

## Methods and analysis

### Study overview

**Figure 1:**
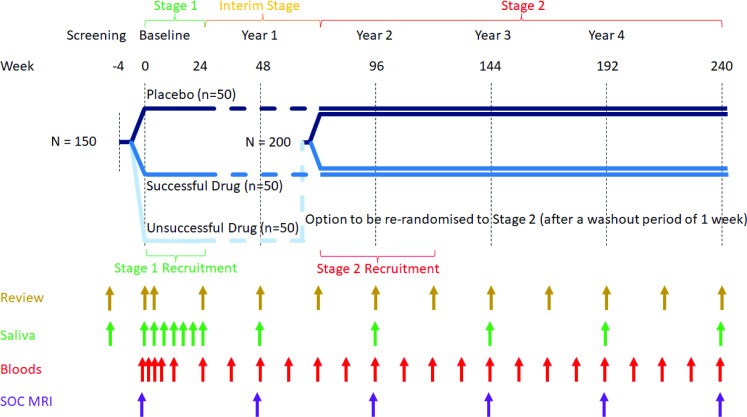
Schematic of STOP-MS clinical trial design indicating anticipated timeline, distribution of participants across study arms, trial stage and potential for re-randomisation. Arrows indicate timing of trial components. SOC, standard of care.

The STOP-MS protocol (currently v4.0 dated 14 February 2025 – online supplemental appendix 2) has been developed in accordance with the Standard Protocol Items: Recommendations for Interventional Trials (SPIRIT) reporting guidelines.^37^ STOP-MS will recruit participants with progressive MS across 20 MS centres spanning all six states in Australia. Sites have been selected on the basis of experience of treating MS, experience of clinical trials, availability and level of training of site staff, for example, International Clinical Harmonization-Good Clinical Practice and EDSS training. Enrolment commenced in April 2025 with an intended mean follow-up period of 3 years (range 2–5 years) across the two stages. STOP-MS will test two active agents (spironolactone and famciclovir) against placebo in a 1:1:1 ratio. The overall trial design is summarised in figure 1. The MAMS trial design aims to efficiently test putative anti-EBV therapies purely on their ability to reduce measures of EBV activity in stage 1 to determine the most likely clinically effective agent. The most effective therapy will then be tested in stage 2 using standard clinical measures of disability as the primary outcome measure. In order to optimise the information gained from STOP-MS, the protocol also includes biobanking and an exploratory cognitive assessment, MSReactor (Monash University, Victoria, Australia).^38^

### Study population

Stage 1 will aim to recruit 150 people with progressive MS, aged 25 to 70 years who have been diagnosed according to the 2017 McDonald criteria.^39^ Stage 2 will involve an additional 200 people meeting the same criteria. The full inclusion and exclusion criteria are listed in box 1.

Box 1 **Inclusion and exclusion criteria**

**Inclusion criteria**

- Age 25–70 years (inclusive).
- Diagnosed with primary or secondary progressive MS according to McDonald 2017 criteria.
- EDSS of 4.0–8.0 (inclusive) at the time of randomisation.
- Evidence of disability progression over the previous 24 months.
- English speaking or non-English speaking but can ensure external interpreter assistance (eg, relative or friend) to attend all visits for the duration of the clinical trial.
- Available to attend clinic visits.

**Exclusion criteria**

- A clinical relapse within 3 months of randomisation.
- A significant comorbidity that in the opinion of the principal investigator would negatively affect MS disease outcomes or preclude administration of spironolactone or famciclovir (including renal failure; estimated glomerular filtration rate <30 mL/min).
- Currently taking medication or supplements known to cause hyperkalaemia as listed (Table 2).
- Hypersensitivity to spironolactone or famciclovir.
- Female participants who are pregnant.
- Female participants who are breastfeeding.
- Women of childbearing potential who are unwilling or unable to use an acceptable method of contraception while on trial treatment and for up to 30 days after the last dose of study drug.
- Have received treatment with steroids (intravenous and/or oral) for MS relapse/progression within 3 months before randomisation.
- Have received any trial therapy within the last 6 months (other than as part of the STOP-MS stage 1 trial).
- Recent or current history of major depression, bipolar disorder, psychosis or suicidality.
- Currently or recently taking any illicit substances (excluding cannabis products used for symptomatic relief).

EDSS, Expanded Disability Status Scale; MS, multiple sclerosis.

### Patient and public involvement

People with lived experience of MS have been involved in every stage of the trial design for this project via formal consumer advocacy pathways. The core research team includes two people with MS. Our design meetings have been regularly attended by a team of people with MS and their input has been actively sought. Our drug selection committee included people with MS and a subcommittee of only people with MS was established to review tolerability profiles and routes of administration for the drugs being considered. Their input contributed 50% of the drug scores used to establish the final shortlist. These contributors were included as authors on a subsequent manuscript^30^ and several are members of the Australian MS Clinical Trials Consortium and our consumer advisory group.

### Standard of care

Participants will be permitted to continue, start or discontinue any currently approved SOC therapy for their form of MS. This was a strong recommendation from the consumer advisory group. Details of SOC DMTs will be documented so that this can be used in ancillary analyses of efficacy.

### Investigational medicinal products

#### Spironolactone

Spironolactone has been shown to reduce tumour necrosis factor-α levels of lipopolysaccharide-activated microglia by >50%.^40^ Spironolactone inhibits EBV replication in the late lytic phase by blocking SM protein.^31^ This occurs as a result of inhibition of xeroderma pigmentosum group B-complementing protein, a component of human transcription factor II H which EBV recruits in the transcription of several late lytic antigens.^41^ In a case report, spironolactone was effective in controlling EBV in a case of non-human immunodeficiency virus, AIDS.^42^ In a small pilot study in MS, a combination of spironolactone and aldosterone was effective in improving symptoms.^32^

#### Famciclovir

Famciclovir exerts its anti-EBV effects through the inhibition of EBV DNA synthesis.^43^ Famciclovir is a prodrug of penciclovir which is then phosphorylated to the active metabolite penciclovir triphosphate. Penciclovir is phosphorylated by herpesvirus thymidine kinase, so this only occurs in herpesvirus-infected cells. Penciclovir inhibits EBV replication in cell culture^44^ and brain concentrations in rats were 41.5% of the concentration in muscle.^45^ The related drug, aciclovir, has been used for the treatment of severe acute EBV infection^46^ and famciclovir has been successfully used to treat a case of overwhelming IM.^47^ Randomised, double-blind, placebo-controlled trials of valaciclovir (a prodrug of aciclovir) have shown a reduction in the number of new active lesions on MRI in those with active disease^48^ and trends towards a reduction in annualised relapse rate and EDSS progression.^34^ A randomised double-blind, placebo-controlled trial of aciclovir showed a trend towards reduction in annualised relapse rate which when dichotomised to low or high relapse rate became statistically significant.^49^ Famciclovir was chosen on the basis of its greater bioavailability, higher intracellular concentration and greater persistence in infected cells.^35^

### Dose and administration

#### SOC plus Spironolactone: - Spironolactone 50 mg twice daily administered orally

Spironolactone is approved for the treatment of essential hypertension, congestive heart failure, cirrhotic liver disease and nephrotic syndrome. It is contraindicated in renal insufficiency, hyperkalaemia and pregnancy. Agents that can cause hyperkalaemia should not be used in conjunction with spironolactone. Adverse events (AEs) include gynaecomastia which is usually reversible on discontinuation, gastrointestinal upset, drowsiness and allergic reactions. For these reasons, we have chosen a medium dose of spironolactone and will screen carefully and monitor throughout the study as outlined below. Dose escalation will also be used to minimise potential AEs. The proposed dose of spironolactone (50 mg two times per day) is likely to result in plasma levels that are in the order of 25%–50% of the half-maximal inhibitory concentration (IC50) for spironolactone and canrenone (table 1).^31^ It is likely the effect of these would be additive (75% of IC50) and other metabolites may also contribute additional anti-EBV effects. This dose has been well tolerated in other settings outside of the main indications for spironolactone and is associated with an acceptable rate of gynaecomastia of less than 10% in males. Therefore, 50 mg two times per day represents the best compromise of potential anti-EBV efficacy and tolerability.

**Table 1:** Pharmacodynamic data for spironolactone and famciclovir

Measure	Spironolactone	Famciclovir
EBV EC_50_		1.5 μg/mL^43^ (penciclovir)
EBV IC_50_	0.87 μg/mL^31^ (spironolactone) 1.16 μg/mL^31^ (canrenone)	5.1 μg/mL^44^ (penciclovir)
t_max_	2.6 hours^79^ (spironolactone) 4.3 hours^79^ (canrenone)	0.75 hours^50^ (penciclovir)
C_max_		
100 mg PO daily	0.21 μg/mL^79^ (spironolactone) 0.43 μg/mL^80^ (canrenone)	
200 mg PO daily	0.67 μg/mL^81^ (canrenone)	
250 mg PO daily		1.6 μg/mL^50^ (penciclovir)
500 mg PO daily		3.3 μg/mL^50^ (penciclovir)
Half-life	2 hours (spironolactone) 16.5 hours^79^ (canrenone)	2.3 hours^50^ (penciclovir)
Protein binding	95%^79^ (canrenone)	<20% (penciclovir)
Excretion	32% urine, 22% bile^80^	90% urine^50^ (penciclovir)
Molecular weight	416.57 g/mol (spironolactone) 340.46 g/mol (canrenone)	321.34 g/mol (famciclovir) 253.26 g/mol (penciclovir)

* Calculated from concentration resulting in >50% reduction of maximal viral replication=2.5 µM.

C_max_, maximum plasma concentration; EBV, Epstein-Barr virus; EC50, half maximal effective concentration; IC50, half maximal inhibitory concentration; PO, per os; t_max_, time to maximum concentration.

#### SOC plus Famciclovir: - Famciclovir 500 mg twice daily administered orally

Famciclovir is a synthetic nucleoside analogue and is approved for the treatment and prevention of herpes zoster and herpes simplex infections (shingles, genital herpes, herpes labialis and herpes encephalitis). Common adverse effects include headache and nausea. Renal impairment is a contraindication. We have chosen the chronic treatment dose to ensure adequate CNS penetrance, and participants will be screened for renal failure before proceeding to study randomisation. The proposed dose of famciclovir (500 mg two times per day) results in plasma levels that are within the predicted half-maximal effective concentration (EC50)/IC50 range for penciclovir and therefore has the potential to be effective against EBV (table 1).^50^ This dose has been well tolerated in prior studies over long periods (up to 12 months) for current indications,^51^,^54^ although the same dose was less well tolerated in a recent cohort of people with MS.^36^

#### SOC plus Placebo: - Matched placebo capsules twice daily administered orally

All capsules will be over-encapsulated in an opaque hard gelatin capsule with sufficient microcrystalline cellulose to fill the capsule by an equivalent amount.

### Dose escalation

To maximise tolerability, both spironolactone and famciclovir will be commenced at half-dose and then increasing to the full-dose, only if well tolerated and all safety parameters are satisfactory at 4 weeks. The inclusion of an option to remain on half-dose, or return to half-dose in the event of tolerability issues, ensures that tolerability can be further optimised. This also reflects what would likely be any future real-world use of these agents.

### Risk mitigation

#### Hyperkalaemia

There is a risk of death with spironolactone through its potential to cause hyperkalaemia.^55^ This principally occurs in the setting of pre-existing medical conditions known to cause hyperkalaemia, such as chronic kidney disease, uncontrolled diabetes, congestive cardiac failure, hyperaldosteronism, congenital adrenal hyperplasia, Addison’s disease, parathyroidectomy or the concomitant use of medications/supplements known to cause hyperkalaemia (eg, ACE inhibitors—see table 2 for full list). All site principal investigators will undergo training with regard to clinical impacts and treatment of hyperkalaemia. All pre-disposing conditions, serum markers of baseline renal impairment (estimated glomerular filtration rate (eGFR) <30 mL/min), serum potassium level >5.0 mmol/L and drugs/supplements that may contribute to hyperkalaemia (online supplemental appendix 3) are exclusion criteria to participation in STOP-MS. Participants will be provided at enrolment with a card indicating the list of drugs and supplements that should be avoided for the duration of the trial. Participants will be advised to indicate their involvement in the trial when being prescribed any new medications and in particular, if being prescribed antibiotics (eg, trimethoprim) or pain medication (eg, non-steroidal anti-inflammatory drugs).

**Table 2:** List of contraindicated medications

Classification	Medications
Potassium sparing diuretic	Amiloride; eplerenone; spironolactone; triamterene
Mineralocorticoid/metabolite	Canrenoate potassium; drospirenone; fludrocortisone
ACE inhibitor	Benazepril; captopril; enalapril; lisinopril; moexipril; perindopril; quinapril; ramipril; trandolapril
Angiotensin II receptor blocker	Azilsartan; candesartan; irbesartan; losartan; olmesartan; telmisartan; valsartan
Beta-blocker	Acebutolol; atenolol; bisoprolol; metoprolol; nadolol; nebivolol; propranolol
NSAID	Celecoxib; diclofenac; ibuprofen; meloxicam; naproxen
Antibiotic/anti-parasitic	Trimethoprim; pentamidine
Epoetin	Epoetin alfa; epoetin beta; epoetin lambda
Other prescribed medicines	Digoxin; enoxaparin; lithium; potassium chloride; sacubitril
Complementary medicine/supplement	Alfalfa; dandelion; horsetail; lily of the valley; milkweed; nettle; muscle-building supplement; salt substitute

ACE, Angiotensin-converting enzyme ; NSAID, Non-steroidal anti-inflammatory drug.

#### Coincidental renal failure

Coincidental development of renal impairment could potentially cause hyperkalaemia with spironolactone^56^ and confusion and drowsiness with famciclovir.^57^ All participants will undergo testing for serum potassium and eGFR at weeks 1, 3, 6 and 12, and then every 12 weeks for the duration of the trial, a potassium level >5.0 mmol/L or eGFR <30 mL/min, if confirmed to not be a spurious result, will be actively managed with either dose reduction or withdrawal of IMP as determined by the site principal investigator. Falling eGFR (>30 mL/min) will be managed as deemed appropriate by the treating physician through any combination of: removal/avoidance of potential precipitants; more frequent testing; reduction to half-dose treatment; cessation of IMP and/or withdrawal from the study. Any AEs of confusion or drowsiness will be investigated with an urgent check of renal function and electrolytes.

#### Gynaecomastia

There is a risk for development of gynaecomastia in males taking spironolactone.^58^ This risk may be related to dose and duration of therapy. Although there are no strategies known to reduce this risk, there is reassuring evidence that this complication of spironolactone therapy is usually reversible on withdrawal of the treatment. All principal investigators will be trained in the clinical assessment of gynaecomastia, with a recommendation for immediate cessation of the trial medication in the case of gynaecomastia being detected.

#### Urinary frequency and urgency

Spironolactone is a diuretic and can therefore worsen or bring out for the first time symptoms of urinary frequency, urgency, nocturia and incontinence in people with MS.^59^ Participants will be warned of the risks of increased urinary symptoms and asked to report these. Severe symptoms may require an adjustment of dosage or withdrawal of IMP.

#### Pregnancy

Spironolactone is potentially harmful to the fetus in pregnancy (category B3) and to the child during breastfeeding.^60^ Famciclovir has been shown to be safe in animals during pregnancy and lactation. There is currently insufficient safety data in humans regarding famciclovir, but some reassuring studies have been published.^61^ Pregnancy and current breastfeeding will be exclusion criteria for participation in STOP-MS. Women of childbearing potential will undergo a pregnancy test at screening and will be excluded if this is positive. Women of childbearing potential will be required to agree to using an acceptable form of contraception for the duration of the trial and for a period of 30 days after ceasing any IMP. Unwillingness or inability to comply with this will be an exclusion criterion to participation in the trial. There is no evidence that spironolactone or famciclovir taken by males is associated with any risk to a fetus conceived by them although spironolactone may affect male fertility.^62^

### Data and safety monitoring board

All AEs will be recorded in the eCRF. All AEs will be coded according to the Common Terminology Criteria for Adverse Events v5.0 (CTCAE - National Cancer Institute, National Institutes of Health, Washington, DC, USA).^63^ Severity will also be graded according to CTCAE v5.0 definitions. Relationship to IMP will be recorded as definitely related, probably related, possibly related, unrelated or prior condition as determined by the site principal investigator. De-identified, aggregate AE data will be reviewed by the Data Safety Monitoring Board (DSMB) every 3 months. Once a minimum of 50 AEs have been reported, these data will be summarised according to treatment allocation (unblinded). Count data comparing active treatment arms with placebo will be analysed using Volcano plots with correction for multiple testing. Proportions of participants with specific AEs will be compared using Fisher’s exact test and frequencies of AEs will be compared using a negative binomial model. Standardised AE rates (rates per person-years) will be analysed with Bayesian random effects models using the Poisson distribution.^64^ Comparisons will be made for all treatment-emergent AEs, treatment-related AEs (definitely or probably related), higher grade AEs (grade 2 or 3) where more than 5 events have occurred and for any SAEs and deaths. Event rates will be compared with reported expected rates of these AEs for the two active treatments using 95% CIs. A summary of AEs will be reported to the lead Human Research Ethics Committee (HREC) committee Research Governance Offices (RGO) annually and any suspected unexpected serious adverse reactions will be reported to the sponsor and RGO within 72 hours, to the Therapeutics Goods Administration within 7 days, if life-threatening (otherwise 15 days), and lead HREC within 30 days. All reports to third parties will be de-identified. Participants who experience any harm as a result of participation in STOP-MS would be able to seek compensation and medical expenses from Griffith University which holds institutional clinical trial insurance.

### Study-related procedures

#### Screening and consenting process

Potential participants will be recruited through the MS Clinics of 10–30 participating sites with a cap of 30 participants at any single centre in stage 1 and an additional 40 participants per site in stage 2. In addition, an online portal (MS Trial Screen) has been created where potential participants can register their interest in a number of MS clinical trials in Australia, including STOP-MS, and assess their eligibility. Details of eligible participants will then be visible to relevant sites through the same portal. Prior to any study-related procedures, written informed consent will be provided. Consent to participation in the biobanking analyses and MSReactor assessments will be optional through an opt-in process. This process will be undertaken by the site principal investigator or their delegate, who will be a trained neurologist with clinical trial experience who has undertaken training on the STOP-MS protocol. Participants will be required to undergo a screening visit prior to enrolment (−4 to −1 weeks) to enable a comprehensive review of medical history, including demographic parameters, details of MS history, other past medical and surgical history, family history, social history and allergies, review of past and current medications and health supplements, comprehensive medical and neurological examination including EDSS score,^65^ and Hospital Anxiety and Depression Scale.^66^ Screening blood tests will include a full blood count (FBC), electrolytes, urea and creatinine (EUC), eGFR, liver function tests (LFT), EBNA1 antibody titre and beta-human chorionic gonadotrophin level (for women of childbearing potential).

#### Trial visits

**Figure 2:**
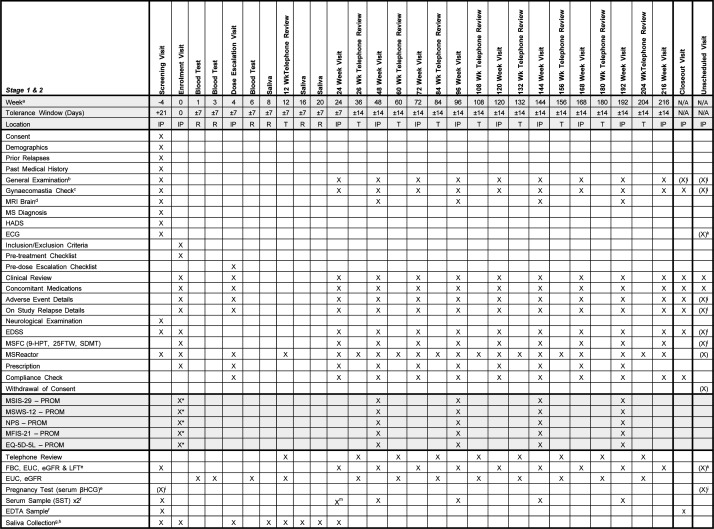
Details of study-related activities at each visit. ‘X’ indicates activity to be performed, (X) indicates procedure may be require (see relevant notes). ^a^, Day 0 and Week 0 are both a single point in time (i.e. lasts one day), all subsequent time points refer to the end of the specified week - if day 0 is a Monday, then week 1 is the following Monday (7 days later) and week -3 will be the Monday 21 days earlier; ^b^, include height, weight, temperature, pulse and blood pressure; ^c^, only required for male participants; ^d^, tolerance window for all MRIs (which are being conducted as parr of standard of care) is ±90 days; ^e^, performed through central labs; ^f^, for biobanking and EBNA1 titres in Stage 1; ^g^, can be performed either at the visit or at home and then posted to QIMR,only required in Stage 1; ^h^, only required in Stage 1; ^i^, only required for women of childbearing potential; ^j^, only required if adverse event suspected; ^k^, only required if hyperkalaemia suspected; ^l^, only required if the unscheduled visit is for a suspected relapse of multiple sclerosis or a suspected increase in disability; ^m^, only required in Stage 1; *, must be completed before or at enrolment. 25FTW, 25 Foot Timed Walk; 9-HPT, 9-Hole Peg Test; ECG, electrocardiogram; EDSS, Expanded Disability Status Scale; EDTA, ethylenediaminetetraacetic acid; eGFR, estimate glomerular filtration rate; EQ-5D-5L, EuroQuol 5 Domains 5 Levels; EUC, electrolytes, urea and creatinine; FBC, full blood count; HADS, Hospital Anxiety and Depression Scale; IP, in-person; LFT, liver function tests; MFIS-21, Modified Fatigue Impact Scale-21; MRI, magnetic resonance imaging; MSFC, Multiple Sclerosis Functional Composite; MSIS-29, Multiple Sclerosis Impact Scale-29; MSWS-12, Multiple Sclerosis Walking Scale-12; NPS, Neuropathic Pain Scale; PROM, patient reported outcome measure; R, remote collection; SDMT, Symbol Digit Modality Test; T, telephone review.

The schedule of trial-related activities is summarised in figure 2. At the enrolment visit (week 0), participants will be reviewed for neurological symptoms and relapse activity, new concomitant medications and supplements, EDSS score, completion of an eligibility criteria checklist and MS Functional Composite (MSFC) score,^67^ including Timed 25 Foot Walk (T25FW),^68^ 9-Hole Peg Test (9-HPT)^69^ and Symbol Digit Modalities Test (SDMT)^70^ in place of the Paced Auditory Serial Addition Test-3.^71^ Patient-reported outcome measures administered online through Research Electronic Data Capture (REDCap - Vanderbilt University, Nashville, TN, USA) will include the MS Impact Scale-29 (MSIS-29),^72^ MS Walking Scale-12 (MSWS-12),^73^ Neuropathic Pain Scale (NPS),^74^ Modified Fatigue Impact Scale (MFIS)^75^ and EuroQol-5 Domains-5 Levels (EQ-5D-5L).^76^ If all eligibility criteria are met, the participant will be enrolled by the site principal investigator, which will involve being randomised and commenced on IMP.

There will be a dose escalation visit at week 4 (±7 days) for review of AEs, concomitant medications and supplements, review of pathology (FBC, LFTs, EUC and eGFR) and a decision regarding dose escalation will be made.

Review visits will be scheduled every 24 weeks (±7 days) to review relapse history, AEs, intercurrent illness, concomitant medications and supplements, current MS SOC therapy, general physical examination, EDSS, MSFC and compliance monitoring (remaining pill count). General examination will include specific examination for gynaecomastia in males.

Telephone monitoring visits will be conducted every 24 weeks (±7 days), offset from review visits by 12 weeks, during which participants will be reviewed for relapse history, AEs and concomitant medications.

#### Safety monitoring blood tests and laboratory outcome measures

Participants will undergo the following blood tests (±7 days): EUC, eGFR at weeks 1, 3, 6 and 12 weeks and then every 12 weeks; FBC, LFT every 24 weeks; EBNA1 titre at week 24 (stage 1 only), week 48 and then every 48 weeks thereafter.

In stage 1, salivary collection for EBV DNA detection will be conducted at weeks −4, 0, 4, 8, 12, 16, 20 and 24.

#### Patient reported outcome measures

The following PROMs will be repeated at annual review visits (weeks 48, 96, 144 and 192): MSIS-29, MSWS-12, NPS, MFIS and EQ-5D-5L.

#### Standard of care MRI

SOC MRI brain will be collected annually provided these are obtained within +/− 3 months of the relevant visit.

#### MSReactor

Participants who opt-in to the MSReactor assessment component of STOP-MS will be invited to complete the 3 tasks of this online cognitive assessment tool that assesses psychomotor speed, visual attention and working memory. This takes only 5 min to complete and will be performed at screening, enrolment and the dose-escalation visit, and then every 12 weeks from the enrolment visit. This tool shows promise as a more sensitive measure of cognitive impairment in MS.

#### follow-up

If the STOP-MS trial progresses to stage 2, follow-up will be for a minimum of 2 years and a maximum of 5 years, with a mean of approximately 3 years.

### Data collection and integrity

Data will be collected in an eCRF created in REDCap. Extensive beta testing of the eCRF was conducted by the core research team and MS Australia staff. The eCRF has been designed with in-built checks for realistic values and internal consistency and will also be checked by an independent monitor from Griffith University. A risk-based approach to monitoring will be used (see online supplemental appendix 4) as both of the IMPs are already Therapeutic Goods Administration (TGA)-approved therapies for other indications with known safety profiles. All sites will be visited remotely or in person after enrolling three participants and if certain criteria are met in relation to eCRF completion and accuracy. Critical data to be reviewed against source documentation at these visits will include consent, eligibility criteria, randomisation, treatment allocation, outcome assessments, AEs and IMP accountability. A copy of the data collection tools is given in online supplemental appendix 5. Data will be entered by study coordinators at each site. All site staff will be required to complete training with regard to assessments: MSFC, SDMT, ECG, standard operating procedures for sample collection for study coordinators, EDSS scoring for principal investigators and independent EDSS assessors. An eCRF completion guideline has been created and distributed to sites. Access to the eCRF is password protected with multifactor authentication, and data access is further restricted according to utilisation requirements, by site and for maintenance of blinding (eg, only pharmacists can see treatment allocation). Participant confidentiality will be maintained using a unique participant identification number with any identifiable information only being visible at the site level. All data will be held at Griffith University for the duration of the trial and for a minimum of 15 years following completion of the trial.

### Blinding

This will be a double-blinded study with both participants and treating physicians blinded as to whether the participant is receiving active treatment or placebo. In addition, due to the potential for unblinding of treating physicians through recognition of well-established adverse effects of one of the repurposed therapies, independent raters will be used for EDSS assessments which is the principal component of the primary clinical outcome measure of stage 2. The randomisation module of REDCap will be used to generate a randomisation code (4 digit number) from the allocation table. Only pharmacists will be able to see the treatment allocation that corresponds to the randomisation code. They will then select the appropriate unblinded bottle of IMP. An unblinded tear-off label identifying the specific IMP contained within bottles will be removed and held on prescription logs within the pharmacy. To maintain study blinding, the IMPs/placebo will be over-encapsulated, so they all look the same. A different capsule will be used for the low-dose and high-dose capsules.

### Randomisation

At the enrolment visit, after eligibility is confirmed and the participant is to be enrolled, they will be randomised to one of the IMPs using the randomisation module of REDCap which selects the next randomisation code from the allocation table. The allocation table will be prepared in Excel, Microsoft (Seattle, CA, USA) stratified by sex (three brackets), age (three brackets) and site. The randomisation codes will be assigned randomly, and the treatment allocations will be randomly assigned in blocks of 3 sorted using the RAND function of Excel. In stage 1, participants will be randomised at enrolment to treatment with spironolactone, famciclovir or placebo at a 1:1:1 ratio. In stage 2, participants will be randomised to either the successful treatment arm from stage 1 (spironolactone or famciclovir) or placebo in a 1:1 ratio. The same randomisation process as above will be used but using a second randomisation schedule with just two treatment allocation options (treatment or placebo) in blocks of two. Randomisation procedures will all be performed by an unblinded monitor under the supervision of the unblinded statistician.

### Unblinding procedure

The randomisation code for an individual participant may only be unblinded in emergency situations. Requests for unblinding can be made by any medical practitioner involved in the participants’ care. It will be recommended that this be done in consultation with the site principal investigator or coordinating principal investigator, but in emergencies, it can be requested directly through the trial monitor who is unblinded.

### Stopping rules

There are no pre-planned stopping rules based on efficacy (futility), but if neither IMP shows a clinically significant difference on measures of EBV activity in stage 1, they will not progress to stage 2 and consideration will be given to trialling alternative agents in a new stage 1 trial. The DSMB will have the remit to stop the trial if a concerning safety profile emerges. This would include a significantly higher rate of any serious AE in any one active treatment arm.

### Loss to follow-up

Participants will be able to withdraw from STOP-MS at any time. If a participant wishes to drop out for any reason, site staff will negotiate with the participant to reduce trial duties with a focus on continued collection of data relating to the primary outcome. If the participant wishes to withdraw completely, wherever possible, the final assessment visit will be brought forward. This will include EDSS, MSFC assessments, AEs and concomitant medications. Wherever possible, reasons for withdrawal or cessation of IMP will be recorded.

### Biobanking

In addition to safety monitoring blood tests, an EDTA blood sample for DNA extraction will be collected at enrolment and serum samples will be collected at enrolment and then every 48 weeks. Serum samples will be centrifuged and divided into 5–8 separate 1 mL aliquots and stored locally at −80°C for later transfer to a central biobank. Details of all samples collected will be held in a separate REDCap database and will include location, stage of analysis, results of analysis and eventual use or destruction.

### Outcomes

#### Stage 1

During stage 1, DNA will be extracted from saliva samples using a saliva DNA extraction kit (Qiagen, Hilden, Germany). EBV DNA will be detected using a TaqMan (Applied Biosystems, Foster City, CA, US) assay and EBV shedding considered present if viral count is >5.8 virus copies/μL. Serum samples will be separated using a centrifuge and pipette. ELISA kits (Diamedix, Miami Lakes, FL, USA) with serial dilutions will be used to measure EBNA1 antibody titres with normalisation to the manufacturer’s cut-off calibrator standard. These tests will be performed at QIMR Berghofer Institute, Queensland, Australia.

#### Stage 2

The stage 2 primary outcome measure will be time to 6mCDP using a composite of EDSS, T25FW and 9-HPT. Definitions of progression will be: an increase in EDSS (of 1 point if baseline EDSS was <5.5, or 0.5 points if baseline EDSS was ≥5.5); ≥20% increase in 9-HPT time; or ≥20% increase in T25FW. Any qualifying change must be confirmed at repeat assessment 6 months later. Inclusion of a measure of upper limb function (9-HPT) also addresses consumer interest in assessing arm function and is particularly important for people with higher levels of disability.

#### Secondary outcome measures

Secondary outcome measures include clinical parameters: time to first relapse, time to 6mCDP based on EDSS alone and mean changes in EDSS, MSFC Score, using the SDMT in place of the Paced Auditory Serial Addition Test, T25FW and 9-HPT. There will also be MRI brain radiological measures including new and enlarging lesion counts between timepoints, and Gd-enhancing lesion counts on cross sectional imaging. On longitudinal imaging, whole brain atrophy (SIENA technique, Siena Imaging, Siena, Italy) will be assessed through the Sydney Neuroimaging Analysis Centre (SNAC). PROMs will include the MSIS-29, which measures physical and psychological well-being, the MSWS-12, NPS and the MFIS every 48 weeks. Cost-effectiveness from a health payer and societal perspective will be measured by cost per quality-adjusted life year (QALY) and will be assessed every 48 weeks using the EQ-5D-5L. For the analysis, results will be reported as the incremental cost per QALY gained as well as the expected net-benefit statistic. We will use societal cost data from our previously published work to attribute avoided societal costs with reduced disability progression.^77^

### Statistical analyses

The co-primary endpoint for STOP-MS stage 1 will be the number of participants with either salivary EBV DNA present in any of the four salivary samples at weeks 12, 16, 20 or 24, or an increase/no change of EBNA1 antibody titre in serum at week 26 compared with baseline. This analysis will be undertaken when all participants in stage 1 have completed the 24-week visit (approximately after 2 years).

The criteria for consideration of progression to stage 2 will be a 10% reduction in the number of participants per active treatment arm meeting the co-primary endpoint. The agent associated with the greatest change in these parameters will be selected for progression to stage 2. Where the lower bound of the CI does not exceed negative value of the clinically meaningful effect size (favouring the treatment group), non-inferiority of the placebo group will be accepted and the treatment arm will be terminated.

In the event of both drugs being tested proving to be highly effective in reducing evidence of EBV activity in stage 1, additional funding will be sought to support a third arm in stage 2. Otherwise, the more effective therapy will be selected. Participants in any unsuccessful arm will be notified and withdrawn from the study and offered the opportunity to be re-screened and randomised in stage 2 after a washout period of 1 week. All trial staff will remain blinded as to treatment allocation of participants remaining in stage 2.

If both agents fail to reach nominal levels of significance in stage 1, consideration will be given to extending stage 1 (in the case of borderline results) or potential continuation of stage 1 with new agents.

For stage 2, the primary analysis will be a Cox-proportional hazards analysis of cumulative hazard of 6mCDP adjusted for potential residual imbalance at baseline for age, sex, SOC DMT and disability level. This analysis will be conducted once the mean period of follow-up exceeds 3 years (approximately after 5 years). Balance of the compared treatment arms on patient demographic, clinical and paraclinical characteristics will be compared at baseline using standardised mean differences. Where the standardised mean differences are 0.2 or greater, the outcome statistical models will be adjusted for the corresponding variables. The proportionality-of-hazards assumption will be evaluated using inspection of Schoenfeld residuals and global test. Participants will be included from the time of their most recent randomisation (baseline). The number of randomisations (with values [1, 2]) will be modelled as a fixed term. Individual records will be right-censored at the time of reaching the outcome of interest or at the last recorded study time-point, whichever occurs first. Secondary outcome measures will be analysed with Cox (or accelerated failure time) models and mixed generalised regression models. Analyses will be conducted on an intention to treat basis. Observations will be censored on leaving the trial for participants without an event, including for those who have withdrawn from the trial, been lost to follow-up or who have died due to causes other than MS. Death due to MS (EDSS 10.0) will count as 6mCDP. Sensitivity analyses will investigate the per-protocol treatment exposure to assess the impact of participant factors (eg, non-compliance). Missing data will be imputed by multiple imputation with missingness-at-random assumed. All analyses will be conducted by JS and LC. The full statistical analysis plan is given in online supplemental appendix 6.

### Sample size calculation

For stage 1 sample size calculations α=0.1, which is suitable for this initial step, was used. Based on the observed rate of salivary samples with EBV DNA in the placebo arms of the teriflunomide clinical trial programme of 40% and a rate of 20% in treated arms, a sample size of 50 participants in each arm would provide 70% power to detect this level of difference. The same sample size would have 80% power to detect a 25% fall in EBNA1 titres towards healthy control levels.

For stage 2, sample size calculations were based on the observed rate of 6mCDP in the placebo arm of the ORATORIO trial of 40%.^78^ A sample size of 132 participants in each arm would have 80% power to detect a 40% reduction in 6mCDP with α=0.05. Allowing for a 14% drop-out rate gives a sample size of 150 in each of 2 arms.

## Registration and dissemination

The STOP-MS trial has a WHO, Unique Trial Number (U1111-1293-1787) and was registered with the Australia and New Zealand Clinical Trial Registry (ANZCTN12623000849695p - https://www.anzctr.org.au/Trial/Registration/TrialReview.aspx?id=386167&isReview=true) on 8 August 2023. The current protocol and statistical analysis are both accessible through this site and will be updated as necessary. The trial has been notified under the Clinical Trial Notification scheme with the Therapeutic Goods Administration (CT-2023-CTN-03 505-1-v1). In accordance with international publication and regulatory requirements, fully de-identified aggregate data and, where necessary, raw data will be made available for independent verification of results and regulatory processes. This data will be available on completion of the trial through Figshare (Digital Science and Research Solutions Ltd, London, UK) and will include de-identified patient level data, data dictionary and analytic codes. The results of each stage of this trial will be published in a leading clinical journal with the aim of submitting within 12 months of the completion of each stage. Participants who have elected to provide contact details on their consent form will be provided with a lay summary of the results on completion of the trial. STOP-MS is sponsored by Griffith University and has been supported by a Medical Research Future Fund grant through the National Health and Medical Research Council from the Department of Health, Disability and Aging, Australian Federal Government.

## Conclusions

STOP-MS has the potential to test novel therapies aimed at controlling EBV activity in people with progressive MS. The premise for trialling these repurposed drugs in this population rests on EBV being both a primary cause of MS and a potential driver of ongoing disease activity, particularly the degenerative component that contributes to progressive MS in the absence of inflammatory disease activity (relapses and new lesions on MRI). EBV might be responsible for ongoing disease activity in MS through a number of mechanisms, including periodic reactivation of auto-reactive B cells either in the periphery or within the nervous system. While the selected agents are unlikely to have any effect on the latent stage of EBV-infected cells, they may suppress reactivation. The STOP-MS trial has been designed in such a way as to explore both the clinical and biological effects of the putative anti-EBV therapies in such a way as to maximise the potential learnings of this treatment approach even in the event of a negative primary clinical outcome. If the outcome of the trial is positive, the MAMS trial design will provide for a more rapid translation to clinical practice and regulatory approval for any successful therapy. At the time of writing, STOP-MS has obtained all necessary regulatory approvals and the first participants have been enrolled.

## Supplementary material

10.1136/bmjno-2025-001313 online supplemental file 1

10.1136/bmjno-2025-001313 online supplemental file 2

10.1136/bmjno-2025-001313 online supplemental file 3

10.1136/bmjno-2025-001313 online supplemental file 4

10.1136/bmjno-2025-001313 online supplemental file 5

10.1136/bmjno-2025-001313 online supplemental file 6
