# Supplementary material for: Study protocol for a multicentre, randomised, double-blinded, placebo-controlled, multi-arm, multi-stage, trial of SpironolacTone and famciclOovir in the treatment of Progressive Multiple Sclerosis to prevent disability progression: the STOP-MS trial
Source: BMJ Neurol Open. 2025 Dec 23;7(2):e001313. doi: 10.1136/bmjno-2025-001313 (PMC12730750; doi:10.1136/bmjno-2025-001313)
Supplement: online supplemental file 1 [file bmjno-7-2-s001.pdf]

Additional members of the Australian MS Clinical Trials Consortium

Member	Affiliation
Suzanne Hodgkinson	University of New South Wales, Australia
Helmut Butzkueven	Monash University, Australia
Rajiv Khanna	QIMR/Berghofer Institute, Australia
Allan Kermode	Perron Institute for Neurological and Translational Science, Australia
Scott Byrne	University of Sydney, Australia
Anneke van der Walt	Monash University, Australia
Sean Riminton	University of Sydney, Australia
Marzena Pedrini	Perron Institute for Neurological and Translational Science, Australia
Stefan Blum	University of Queensland, Australia
Stephanie Trend	Perron Institute for Neurological and Translational Science, Australia
Heidi Beadnall	University of Sydney, Australia
Zara Ioannides	University of Queensland, Australia
Nevin John	Monash University, Australia
Izanne Roos	University of Melbourne, Australia
Janakan Ravindran	University of Adelaide, Australia
Tim Wang	University of Sydney, Australia
Trevor Kilpatrick	University of Melbourne, Australia
Anne-Louise Ponsonby	University of Melbourne, Australia
Pam McCombe	University of Queensland, Australia
Richard Macdonell	University of Melbourne, Australia
Tri Phan	Garvan Institute, Australia
Silvana Gaudieri	University of Western Australia, Australia
John Parratt	Royal North Shore Hospital, Australia

Anne Bruestle	Australian National University, Australia
Steven Petratos	Monash University, Australia
Sanjay Swaminathan	Westmead Hospital, Australia
Vicki Maltby	University of Newcastle, Australia
Yuan Zhou	University of Tasmania, Australia
Mastura Monif	Monash University, Australia
Joshua Barton	Sunshine Coast University Hospital
Chhavi Asthana	University of Tasmania, Australia
Lauren Giles	University of Tasmania, Australia
Julia Morahan	Multiple Sclerosis Australia, Australia
Nigel Caswell	People with MS - Victoria, Australia
Amit Bar-Or	University of Pennsylvania, USA
Michael Levy	Harvard University, USA
Gavin Giovannoni	University College London, UK
