# Supplementary material for: Study protocol for a multicentre, randomised, double-blinded, placebo-controlled, multi-arm, multi-stage, trial of SpironolacTone and famciclOovir in the treatment of Progressive Multiple Sclerosis to prevent disability progression: the STOP-MS trial
Source: BMJ Neurol Open. 2025 Dec 23;7(2):e001313. doi: 10.1136/bmjno-2025-001313 (PMC12730750; doi:10.1136/bmjno-2025-001313)
Supplement: online supplemental file 2 [file bmjno-7-2-s002.pdf]

# Phase III, multicentre, randomised, double-blinded, placebo-controlled, MAMS trial of SpironolacTone and famciclOvir in the treatment of Progressive MS to prevent disability progression (STOP-MS)

Version Number: 4.0

Amendment 5

Date: 14/02/2025

World Health Organisation, Universal Trial Number: U1111-1293-1787

Australian and New Zealand Clinical Trial Registry Number:

ACTN12621001502820p (registered 8/08/2023)

Local Ref: PLAT002

**Sponsor: Griffith University**

**Statement of Compliance**

This document is a protocol for a research project. This study will be conducted in compliance with all stipulations of this protocol, the conditions of the ethics committee approval, the NHMRC National Statement on Ethical Conduct in Human Research (2007) – Updated 2018, and the NHMRC and Universities Australia Australian Code for the Responsible Conduct of Research (2018). As a clinical trial, the study will also comply with the Note for Guidance on Good Clinical Practice (CPMP/ICH-135/95).

**Signature Page**

The undersigned confirm that the following protocol has been agreed and accepted and that the Chief Investigator agrees to conduct the study in compliance with the approved protocol and will adhere to the principles outlined in the Declaration of Helsinki, the Sponsor's SOPs, and other regulatory requirements.

I agree to ensure that the confidential information contained in this document will not be used for any other purpose other than the evaluation or conduct of the investigation without the prior written consent of the Sponsor.

I also confirm that I will make the findings of the study publicly available through publication or other dissemination tools without any unnecessary delay and that an honest accurate and transparent account of the study will be given; and that any discrepancies from the study as planned in this protocol will be explained.

Principal Investigator:

Signature: ______________________ Date: 08 / APR / 2025

Name (please print): Simon Broadley

Position: Coordinating Principal Investigator

## Contents

Study Investigators........ 5
Study Synopsis........ 15
Glossary of Abbreviations, Terms, and Acronyms........ 17
1. Background........ 19
1.1. The Problem........ 19
1.2. Comprehensive understanding of the cause of MS........ 19
1.3. The Solution........ 20
1.3.1. Innovative and informed approach to trial design........ 20
1.3.2. Rationale for adaptive trial design........ 20
1.4. EBV biology........ 21
1.5. Systematic approach to drug selection........ 22
1.6. Evidence-based outcome measures........ 22
1.7. Impact........ 23
1.8. Proposed Trial........ 24
1.9. Regulatory Approval........ 27
1.9.1. Trial Registration........ 27
1.9.2. Human Research Ethics Committee Approval........ 28
1.9.3. Governance approval........ 28
2. Study Objectives........ 29
2.1. Research Question and Aims/Objectives........ 29
2.1.1. Primary Aims/Objectives........ 29
2.1.1.1. Stage 1........ 29
2.1.1.2. Stage 2........ 29
2.1.2. Secondary Aims/Objectives........ 29
2.2. Hypotheses........ 29
3. Methods........ 31
3.1. Methodological Approach........ 31
3.2. Study Sites/Settings........ 31
3.3. Study Population........ 31
3.3.1. Inclusion criteria........ 32
3.3.2. Exclusion criteria........ 32
3.4. Recruitment/Selection........ 33
3.4.1. Randomisation........ 33
3.4.2. Blinding and allocation concealment........ 34

3.4.3. Labelling of IMP ... 34
3.4.4. Breaking of study blinding ... 35
3.4.5. On completion of the study ... 35
3.5. Consent ... 35
3.6. Risk Mitigation Procedures ... 36
3.7. Participant Withdrawal Procedures ... 41
3.7.1. Screen Failure ... 41
3.7.2. Lost to Follow Up ... 41
3.7.3. Stopping Rules ... 41
3.8. Study Procedures ... 42
3.8.1. Treatment arms ... 42
3.8.1.1. Intervention Description, Dosage and Route of Administration ... 42
3.8.1.2. Blinding ... 43
3.8.1.3. Concurrent MS therapies ... 43
3.8.1.4. Dose escalation ... 43
3.8.1.5. Dose reduction ... 43
3.8.2. Study Drug Accountability ... 43
3.8.2.1. Control of Supplies ... 43
3.8.2.2. Return of Supplies ... 43
3.8.2.3. Retention of Samples ... 43
3.8.3. Study Procedures and Visits ... 44
4. Translation to Changes in Clinical Practice ... 54
5. Timeline ... 54
6. Funding and Resources ... 55
6.1. Governance ... 55
6.1.1. Independent Steering Committee ... 55
6.1.2. Investigator Meetings ... 56
6.1.3. Data Safety Monitoring Board ... 56
6.1.4. Sponsor ... 56
6.1.5. Trial Site Resources ... 57
7. References ... 58

## Study Investigators

| | |
|---|---|
| **Coordinating Chief Investigator:** | Name: Prof Simon Broadley<br>Institution: Griffith University & Gold Coast University Hospital<br>Department: School of Medicine and Dentistry<br>Address: 1 Parklands Drive, Southport QLD 4215<br>Tel: 0466 207 444<br>Email: simon.broadley@griffith.edu.au<br>Role in Study: Coordinating CI and Site sub-investigator |
| **Co-Investigator:** | Name: Prof Bruce Taylor<br>Institution: University of Tasmania<br>Department: Menzies Institute for Medical Research<br>Address: 17 Liverpool Street, Hobart TAS 7000<br>Tel: 0409 231 919<br>Email: bruce.taylor@utas.edu.au<br>Role in Study: Study Design and Site PI |
| **Co-Investigator:** | Name: Prof Michael Barnett<br>Institution: University of Sydney<br>Department: Brain and Mind Centre<br>Address: Camperdown NSW 2006<br>Tel: 0416 288 208<br>Email: mbarnett@mail.usyd.edu.au<br>Role in Study: Study Design and Site PI |
| **Co-Investigator:** | Name: Prof Jeremy Chataway<br>Institution: University College London<br>Department: Neurology<br>Address: National Hospital for Neurology and Neurosurgery, Queen Square, London WC1N 3BG, UK<br>Tel: +44 02724 752295<br>Email: j.chataway@ucl.ac.uk<br>Role in Study: Study Design |
| **Co-Investigator:** | Name: Prof Mahesh Parmar<br>Institution: University College London<br>Department: MRC Clinical Trials Unit<br>Address: 90 High Holborn, London WC1V 6LJ, UK<br>Tel: +44 020 7670 4700<br>Email: m.parmar@ucl.ac.uk<br>Role in Study: Study Design and Statistical Plan |
| **Co-Investigator:** | Name: Prof Lawrence Steinman<br>Institution: Stanford University<br>Department: Beckman Centre for Molecular Medicine<br>Address: 279 Campus Drive, Stanford CA 94305-5316, USA<br>Tel: +1 (650) 725-6401<br>Email: steiny@stanford.edu<br>Role in Study: Study Design and Rationale |
| **Co-Investigator:** | Name: Professor David Tscharke<br>Institution: Australian National University<br>Department: Curtin School of Medical Research<br>Address: Raymond Terrace, South Brisbane QLD 4101<br>Tel: 02 6125 3020<br>Email: david.tscharke@anu.edu.au<br>Role in Study: Study Design and Person With MS |

| | |
|---|---|
| **Co-Investigator:** | Name: Prof Tomas Kalincik<br>Institution: University of Melbourne<br>Department: Royal Melbourne Hospital<br>Address: 300 Grattan St, Parkville VIC 3050<br>Tel: 0402 776 759<br>Email: tomas.kalincik@unimelb.edu.au<br>Role in Study: Study Design and Site PI |
| **Co-Investigator:** | Name: A/Prof Vilija Jokubaitis<br>Institution: Monash University<br>Department: Monash Data Futures Institute<br>Address: 13 Rainforest Walk, Clayton Campus, Clayton VIC 3800<br>Tel: 03 9903 0880<br>Email: vilija.Jokubaitis@monash.edu<br>Role in Study: Study Design, Rationale and Laboratory Work |
| **Co-Investigator:** | Name: Prof Corey Smith<br>Institution: QIMR Berghofer Institute<br>Department: Translational and Human Immunology<br>Address: 300 Herston Rd, Herston QLD 4006<br>Tel: 07 3362 0222<br>Email: corey.smith@qimrberghofer.edu.au<br>Role in Study: Study Design, Rationale and Laboratory Work |
| **Co-Investigator:** | Name: Prof Jing Sun<br>Institution: Griffith University<br>Department: Menzies Health Institute Queensland<br>Address: Parklands Drive, Southport QLD 4215<br>Tel: 07 5678 0924<br>Email: j.sun@griffith.edu.au<br>Role in Study: Statistician |
| **Co-Investigator:** | Name: Dr Julie Campbell<br>Institution: University of Tasmania<br>Department: Menzies Institute for Medical Research<br>Address: 17 Liverpool Street, Hobart TAS 7000<br>Tel: 03 6226 7700<br>Email: julie.campbell@utas.edu.au<br>Role in Study: Health Economist |
| **Co-Investigator:** | Name: Dr Vivien Li<br>Institution: University of Melbourne<br>Department: Royal Melbourne Hospital<br>Address: 300 Grattan St, Parkville VIC 3010<br>Tel: 03 9342 7000<br>Email: vivien.li@unimelb.edu.au<br>Role in Study: Study Design and Site AI |
| **Co-Investigator:** | Name: Dr Grant Parnell<br>Institution: University of Sydney<br>Department: The Westmead Institute for Medical Research<br>Address: 176 Hawkesbury Rd, Westmead NSW 2145<br>Tel: 02 8627 3915<br>Email: grant.parnell@sydney.edu.au<br>Role in Study: Study Design and Rationale |

| | |
|---|---|
| **Co-Investigator:** | Name: A/Prof Sudarshini Ramanathan<br>Institution: University of Sydney<br>Department: Translational Neuroimmunology Group<br>Address: 178A Hawkesbury Rd, Westmead NSW 2145<br>Tel: 0413 314 685<br>Email: sudarshini.ramanathan@sydney.edu.au<br>Role in Study: Study Design and Site PI |
| **Co-Investigator:** | Name: Dr Belinda Kaskow<br>Institution: University of Western Australia<br>Department: Perron Institute for Neurological and Translational Science<br>Address: QE II Medical Centre Ralph & Patricia Sarich Neuroscience Building, 8 Verdun St, Nedlands WA 6009<br>Tel: 08 6457 0222<br>Email: belinda.kaskow@perron.uwa.edu.au<br>Role in Study: Study Design and Rationale |
| **Co-Investigator:** | Name: Dr Katherine Buzzard<br>Institution: Monash University<br>Department: Eastern Health Clinical School Research<br>Address: 5 Arnold Street, Box Hill, VIC 3128<br>Tel: 03 9500 8366<br>Email: katherine.buzzard@easternhealth.org.au<br>Role in Study: Site PI |
| **Co-Investigator:** | Name: Dr Lesley-Ann Hall<br>Institution: Flinders Medical Centre<br>Department: Department of Neurology<br>Address: Flinders Dr, Bedford Park SA 5042<br>Tel: 08 8204 7777<br>Email: lesley-ann.hall@sa.gov.au<br>Role in Study: Site PI |
| **Co-Investigator:** | Name: A/Prof Kaylene Young<br>Institution: University of Tasmania<br>Department: Menzies Institute for Medical Research<br>Address: 17 Liverpool Street, Hobart TAS 7000<br>Tel: 0427 618 366<br>Email: kaylene.young@utas.edu.au<br>Role in Study: Study Design and Rationale |
| **Co-Investigator:** | Name: Dr Jennifer Massey<br>Institution: St Vincent’s Hospital Sydney<br>Department: Kinghorn Cancer Centre and St Vincent’s Centre for Applied Medical Research<br>Address: 370 Victoria St, Darlinghurst NSW 2010<br>Tel: 02 9355 5656<br>Email: jennifer.massey@svha.org.au<br>Role in Study: Site PI |
| **Co-Investigator:** | Name: Prof William Carroll<br>Institution: University of Western Australia<br>Department: Perron Institute for Neurological and Translational Science<br>Address: 8 Verdun St, Nedlands WA 6009<br>Tel: 08 6457 0222<br>Email: wm.carroll@me.com<br>Role in Study: Member of Australian MS Clinical Trials Platform Steering Committee |

| | |
|---|---|
| **Co-Investigator:** | Name: Mr Andrew Potter<br>Institution: c/o MS Australia<br>Department: Head Office<br>Address: 100 Miller Street, North Sydney NSW 2060<br>Tel: 1300 010 158<br>Email: andrew.potter@msaustralia.org.au<br>Role in Study: Study Design and Person With MS |
| **Co-Investigator:** | Name: Professor Richard Macdonell<br>Institution: Austin Health<br>Department: Department of Neurology<br>Address: 145 Studley Road, Heidelberg VIC 3084<br>Tel: 0401 675 308<br>Email: richard.macdonell@austin.org.au<br>Role in Study: Site PI |
| **Co-Investigator:** | Name: Professor Pamela McCombe<br>Institution: University of Queensland<br>Department: Centre for Clinical Research<br>Address: 71/918. Royal Brisbane & Women's Hospital Campus Herston, QLD, 4029<br>Tel: 07 3646 8111<br>Email: pamela.mccombe@uq.edu.au<br>Role in Study: Chair of Australian MS Clinical Trials Platform Steering Committee |
| **Co-Investigator:** | Name: Dr Zara Ioannides<br>Institution: Royal Brisbane and Women's Hospital<br>Department: Department of Neurology<br>Address: Butterfield Street, Herston QLD 4029<br>Tel: 0405 270 009<br>Email: zara.ioannides@health.qld.gov.au<br>Role in Study: Site PI |
| **Co-Investigator:** | Name: Dr Laura Clarke<br>Institution: Princess Alexandra Hospital<br>Department: Department of Neurology<br>Address: 199 Ipswich Road, Woolloongabba QLD 4102<br>Tel: 0412 442 417<br>Email: laura.clarke@health.qld.gov.au<br>Role in Study: Site PI |
| **Co-Investigator:** | Name: A/Prof Stefan Blum<br>Institution: Mater Hospital<br>Department: Department of Neurology<br>Address: Raymond Terrace, South Brisbane QLD 4101<br>Tel: 0410 777 184<br>Email: stefan.blum@brisbaneneurology.com.au<br>Role in Study: Site PI |
| **Co-Investigator:** | Name: Dr Joshua Barton<br>Institution: Sunshine Coast University Hospital<br>Department: Department of Neurology<br>Address: 6 Doherty Street, Birtinya QLD 4575<br>Tel: 0408 770 951<br>Email: joshua.barton@health.qld.gov.au<br>Role in Study: Site PI |

| | |
|---|---|
| **Co-Investigator:** | Name: Professor Suzanne Hodgkinson<br>Institution: Liverpool Hospital<br>Department: Department of Neurology<br>Address: Corner of Elizabeth and Goulburn Streets, Liverpool NSW 2170<br>Tel: 0429 573 234<br>Email: s.hodgkinson@unsw.edu.au<br>Role in Study: Site PI |
| **Co-Investigator:** | Name: Professor Jeannette Lechner Scott<br>Institution: John Hunter Hospital<br>Department: Department of Neurology<br>Address: Lookout Rd, New Lambton Heights NSW 2305<br>Tel: 0402 964 260<br>Email: jeannette.lechnerscott@health.nsw.gov.au<br>Role in Study: Site PI |
| **Co-Investigator:** | Name: Professor Anneke Van der Walt<br>Institution: The Alfred Hospital<br>Department: Department of Neurology<br>Address: 55 Commercial Rd, Melbourne VIC 3004<br>Tel: 03 9076 2000<br>Email: anneke.vanderwalt@monash.edu<br>Role in Study: Study design |
| **Co-Investigator:** | Name: Professor Helmut Butzkueven<br>Institution: The Alfred<br>Department: Department of Neurology<br>Address: 55 Commercial Rd, Melbourne VIC 3004<br>Tel: 0414 881 494<br>Email: helmut.butzkueven@monash.edu<br>Role in Study: Study design and site PI |
| **Co-Investigator:** | Name: Professor Todd Hardy<br>Institution: Concord Hospital<br>Department: Department of Neurology<br>Address: Hospital Rd, Concord NSW 2139<br>Tel: 02 9767 5000<br>Email: thar6109@sydney.edu.au<br>Role in Study: Study design and site sub-investigator |
| **Co-Investigator** | Name: Dr Marion Simpson<br>Institution: Austin Health<br>Department: Department of Neurology<br>Address: 145 Studley Road, Heidelberg VIC 3084<br>Tel: 03 9496 5529<br>Email: marion.simpson@austin.org.au<br>Role in Study: Site sub-investigator |
| **Co-Investigator** | Name: Dr Izanne Roos<br>Institution: University of Melbourne<br>Department: Royal Melbourne Hospital<br>Address: 300 Grattan St, Parkville VIC 3050<br>Tel: 0402 776 759<br>Email: izanne.roos@mh.org.au<br>Role in Study: Site sub-investigator |

| | |
|---|---|
| **Co-Investigator** | Name: A/Prof Mastura Monif<br>Institution: The Alfred Hospital<br>Department: Department of Neurology<br>Address: 55 Commercial Rd, Melbourne VIC 3004<br>Tel: 03 9076 2000<br>Email: mastura.monif@monash.edu<br>Role in Study: Study Design and Site sub-investigator |
| **Co-Investigator** | Name: Prof Allan Kermode<br>Institution: University of Western Australia<br>Department: Perron Institute for Neurological and Translational Science<br>Address: 8 Verdun St, Nedlands WA 6009<br>Tel: 0407 085 945<br>Email: kermode@me.com<br>Role in Study: Site PI |
| **Co-Investigator** | Name: Dr Nevin John<br>Institution: Monash Health<br>Department: Department of Neurology<br>Address: 246 Clayton Rd, Clayton VIC 3168<br>Tel: 03 9594 6666<br>Email: nevin.john@monash.edu<br>Role in Study: Site PI |
| **Co-Investigator** | Name: Dr Janakan Ravindran<br>Institution: Royal Adelaide Hospital<br>Department: Department of Neurology<br>Address: Port Rd, Adelaide SA 5000<br>Tel: 0412 349 734<br>Email: janakan.ravindran@sa.gov.au<br>Role in Study: Site PI |
| **Co-Investigator** | Name: Dr Lauren Giles<br>Institution: Launceston General Hospital<br>Department: Department of Neurology<br>Address: 274-280 Charles St Launceston TAS 7250<br>Tel: 03 6777 6777<br>Email: laurenpgiles@gmail.com<br>Role in Study: Site PI |
| **Co-Investigator** | Name: Dr John Parratt<br>Institution: Royal North Shore Hospital<br>Department: Department of Neurology<br>Address: Reserve Road, St Leonards NSW 2065<br>Tel: 0437 548 433<br>Email: john@sharpneurology.com<br>Role in Study: Site PI |
| **Co-Investigator** | Name: Prof Trevor Kilpatrick<br>Institution: University of Melbourne<br>Department: Royal Melbourne Hospital<br>Address: 300 Grattan St, Parkville VIC 3050<br>Tel: 0402 776 759<br>Email: tkilpat@unimelb.edu.au<br>Role in Study: Member of Steering Committee |

| | |
|---|---|
| **Co-Investigator** | Name: Dr Andrew Henderson<br>Institution: Westmead Hospital<br>Department: Department of Neurology<br>Address: Cnr Hawkesbury Road and, Darcy Rd, Westmead NSW 2145<br>Tel: 02 8890 5555<br>Email: andrew.henderson@health.nsw.gov.au<br>Role in Study: Site PI |
| **Co-Investigator** | Name: Professor Andrew Lloyd<br>Institution: University of New South Wales<br>Department: The Kirby Institute<br>Address: Wallace Wurth Building, Cnr High St & Botany St, UNSW, Kensington NSW 2052<br>Tel: 02 9385 2534<br>Email: a.lloyd@unsw.edu.au<br>Role in Study: Trial design |
| **Co-Investigator** | Name: A/Prof Silvana Guadieri<br>Institution: University of Western Australia<br>Department: School of Human Sciences<br>Address: 35 Stirling Highway, Perth WA 6009<br>Tel: 08 6488 1096<br>Email: silvana.gaudieri@uwa.edu.au<br>Role in Study: Trial design |
| **Co-Investigator** | Name: Professor Gavin Giovannoni<br>Institution: Queen Mary University of London<br>Department: Centre for Neuroscience, Surgery and Trauma<br>Address: 4 Newark St, London E1 2AT, UK<br>Tel: +44 020 7882 8954<br>Email: g.giovannoni@qmul.ac.uk<br>Role in Study: International advisor |
| **Co-Investigator** | Name: Professor Amit Bar-Or<br>Institution: University of Pennsylvania<br>Department: Perelman School of Medicine<br>Address: 3400 Spruce Street – 3 Gates Building<br>Philadelphia, PA 19104, USA<br>Tel: +1 (215) 662-3606<br>Email: amitbar@pennmedicine.upenn.edu<br>Role in Study: International advisor |
| **Co-Investigator** | Name: Professor Steve Vucic<br>Institution: University of Sydney<br>Department: Concord Clinical School<br>Address: Hospital Rd, Concord NSW 2139<br>Tel: 02 9767 8447<br>Email: steve.vucic@sydney.edu.au<br>Role in Study: Site sub-investigator |
| **Co-Investigator** | Name: Professor Anne-Louise Ponsonby<br>Institution: University of Melbourne<br>Department: Murdoch Children's Research Institute<br>Address: Cnr Hawkesbury Road and, Darcy Rd, Westmead NSW 2145<br>Tel: 03 8341 6200<br>Email: anne-louise.ponsonby@mcri.edu.au<br>Role in Study: Trial design |

| | |
|---|---|
| **Co-Investigator** | Name: Dr Timothy Spelman<br>Institution: University of Melbourne<br>Department: Burnet Institute<br>Address: 85 Commercial Rd, Melbourne VIC 3004<br>Tel: 03 9282 2111<br>Email: tim@burnet.edu.au<br>Role in Study: Statistician |
| **Co-Investigator** | Name: A/Prof Anne Bruestle<br>Institution: Australian National University<br>Department: John Curtin School of Medical Research<br>Address: 131 Garran Rd, Acton ACT 2601<br>Tel: 02 6125 9009<br>Email: anne.bruestle@anu.edu.au<br>Role in Study: Biomarker analysis |
| **Co-Investigator** | Name: Professor Tri Phan<br>Institution: Garvan Institute<br>Department: Intravital Microscopy Lab<br>Address: 384 Victoria St, Darlinghurst NSW 2010<br>Tel: 02 9295 8100<br>Email: t.phan@garvan.org.au<br>Role in Study: Site PI |
| **Co-Investigator** | Name: Professor Rajiv Khanna<br>Institution: QIMR/Berghofer Medical Research Institute<br>Department: Immunology Department<br>Address: 300 Herston Rd, Herston QLD 4006<br>Tel: 07 3362 0385<br>Email: rajiv.khanna@qimrberghofer.edu.au<br>Role in Study: Advisor on EBV biology |
| **Co-Investigator** | Name: Professor Michael Levy<br>Institution: Harvard University<br>Department: Mass General Research Institute<br>Address: CNY-Building #114, 114 16th Street, 3150, Charlestown, MA 02129-2000, USA<br>Tel: +1 (617) 726-0412<br>Email: mlevy11@mgh.harvard.edu<br>Role in Study: International advisor |
| **Co-Investigator** | Name: Dr Marzena Fabis-Pedrini<br>Institution: University of Western Australia<br>Department: Perron Institute for Neurological and Translational Science<br>Address: QE II Medical Centre Ralph & Patricia Sarich Neuroscience Building, 8 Verdun St, Nedlands WA 6009<br>Tel: 08 6457 0218<br>Email: marzena.pedrini@perron.uwa.edu.au<br>Role in Study: Biomarker analysis |
| **Co-Investigator** | Name: Mr Nigel Caswell<br>Institution: c/o MS Australia<br>Department: Research Department<br>Address: Level 19, Northpoint, 100 Miller Street, North Sydney NSW 2060<br>Tel: 1300 010 158<br>Email: caswellnigelp13@gmail.com<br>Role in Study: Person with MS |

| | |
|---|---|
| **Co-Investigator** | Name: Professor Leonid Churilov<br>Institution: Royal Melbourne Hospital<br>Department: Department of Medicine<br>Address: Building 104, Alan Gilbert Building University of Melbourne, 161 Barry St, Carlton VIC 3010<br>Tel: 03 8344 5892<br>Email: leonidc@unimelb.edu.au<br>Role in Study: Statistician |
| **Co-Investigator** | Name: Dr Jason Burton<br>Institution: University of Western Australia<br>Department: Perron Institute for Neurological and Translational Science<br>Address: QE II Medical Centre Ralph & Patricia Sarich Neuroscience Building, 8 Verdun St, Nedlands WA 6009<br>Tel: 08 9332 2861<br>Email: jason.burton@health.wa.gov.au<br>Role in Study: Site sub-investigator |
| **Co-Investigator** | Name: Dr Heidi Beadnall<br>Institution: University of Sydney<br>Department: Brain and Mind Centre<br>Address: 94 Mallett St, Camperdown NSW 2006<br>Tel: 0416 288 208<br>Email: heidi@mail.usyd.edu.au<br>Role in Study: Study Design and Site sub-investigator |
| **Co-Investigator** | Name: Dr Kayla Ward<br>Institution: Griffith University & Gold Coast University Hospital<br>Department: Department of Neurology<br>Address: 1 Hospital Boulevard, Southport QLD 4215<br>Tel: 0413 075 060<br>Email: kayla.ward@health.qld.gov.au<br>Role in Study: Site PI |

**Australian MS Clinical Trials Platform Governance**

The above team are conducting three nationwide clinical trials in MS. To provide efficiencies of scale and expertise we have determined that a combined Governance structure will be appropriate. We have therefore proposed the “Australian MS Clinical Trials Platform”, which will be administered by MS Australia. We propose that the three clinical trials (PLATYPUS, STOP-MS and FIRMS-EBV) will be overseen by the following committees.

***Steering Committee***

This committee will provide strategic direction and advice to the Coordinating Chief Investigators (Professor Simon Broadley and Professor Todd Hardy). Several prominent national and international authorities on MS clinical trials have already agreed to be members of this committee.

Professor Pamela McCombe has kindly agreed to chair this committee. Please see Appendix 1 for the terms of reference for this committee.

***Data Safety Monitoring Board (DSMB)***

This board will review safety reports and adverse event reports for the three trials.

The chair and members for this committee are yet to be selected. Please see Appendix 2 for the terms of reference for this board.

***Consumer Engagement Committee***

This committee will provide strategic advice on consumer engagement and external representation.

This committee will be constituted wholly by people with MS who will advise on matters relating to participants and external engagement. Professor David Tscharke has kindly agreed to chair this committee. Please see Appendix 3 for the terms of reference for this committee.

***Recruitment and Retention Committee***

This committee will review recruitment and retention progress in the trials.

This committee will be constituted by both people with MS and others with expertise in MS clinical trials and the MS community. Mr Andrew Potter has kindly agreed to chair this committee. Please see Appendix 4 for the terms of reference for this committee.

## Study Synopsis

| | |
|---|---|
| **Title:** | Phase III, multicentre, randomised, double-blinded, placebo-controlled, MAMS trial of SpironolacTone and famciclOvir in the treatment of Progressive Multiple Sclerosis to prevent disability progression (STOP-MS) |
| **Short Title:** | STOP-MS |
| **Study Sites:** | Griffith University, QLD<br>Royal Brisbane and Women's Hospital, QLD<br>Princess Alexandra Hospital, QLD<br>Mater Hospital Brisbane, QLD<br>Sunshine Coast Hospital, QLD<br>Menzies Institute for Medical Research, University of Tasmania, TAS<br>Launceston General Hospital, TAS<br>Brain and Mind Centre, NSW<br>Concord Hospital Sydney NSW<br>St Vincent's Hospital Sydney, NSW<br>Royal North Shore Hospital, NSW<br>Westmead Hospital, NSW<br>Liverpool Hospital, NSW<br>John Hunter Hospital, NSW<br>Royal Melbourne Hospital, VIC<br>Monash Medical Centre, VIC<br>Alfred Hospital, VIC<br>Austin Hospital, VIC<br>Box Hill Hospital, VIC<br>Flinders Medical Centre, SA<br>Royal Adelaide Hospital, SA<br>Perron Institute, WA |
| **Study Aims/ Objectives/Hypothesis:** | Stage 1: to demonstrate that spironolactone or famciclovir plus SOC reduce the frequency of EBV DNA being present in saliva and/or reduce EBNA1 antibody titres in people with progressive MS when compared to placebo plus SOC.<br>Stage 2: to demonstrate that spironolactone or famciclovir plus SOC reduce the likelihood of 6mCDP in people with progressive MS when compared to placebo plus SOC.<br>Secondary aims - to demonstrate that spironolactone or famciclovir plus SOC:<br>a. are safe when used to treat people with progressive MS<br>b. reduce the rate of brain atrophy at 3 years compared to placebo plus SOC<br>c. reduce the numbers of new/expanded T2/ FLAIR and Gd-enhancing lesions on MRI brain compared to placebo plus SOC<br>d. reduce the level of whole brain atrophy on MRI brain compared to placebo plus SOC<br>e. improve PROMs of disease impact compared to placebo plus SOC.<br>f. are cost-effective. |

| | |
|---|---|
| **Study Design:** | This is a multicentre, randomised, double-blinded, placebo-controlled, MAMS phase III clinical trial. |
| **Study Outcome Measures:** | **Stage 1:** co-primary outcome measures of salivary EBV DNA detection (viral shedding) and serum EBNA1 antibody titres.<br>**Stage 2:** time to 6mCDP using a composite of EDSS, T25FW and 9-HPT.<br>**Secondary outcome measures:**<br>Clinical – time to first relapse, time to 6mCDP using EDSS only, mean change in EDSS and MSFC scores.<br>MRI – new and enlarging lesion counts and whole brain atrophy.<br>PROMs – MSIS-29, MSWS-12, Neuropathic Pain Scale and FSMCF.<br>Health economics – EQ-5D-5L. |
| **Study Population:** | People with progressive MS (primary progressive or secondary progressive) either on current DMT or on no treatment. |
| **Number of participants:** | Stage 1: n = 150 (3 x 50 per arm)<br>Stage 2: n = 200 (2 x 100 per arm – additional)<br>Total = 350 |
| **Translation to Clinical Practice:** | If spironolactone or famciclovir prove to be effective in reducing disability progression in people with progressive MS, the research team will apply to the Therapeutic Goods Administration for a new indication (progressive MS) for the relevant drug and prepare new treatment guidelines. |
| **Key Ethical and Safety Considerations:** | People with progressive MS are a vulnerable group both in terms of disability level and potential cognitive impairment. This combined with a current paucity of effective treatment options can lead to a sense of desperation. It will be essential to ensure that the study design incorporates safeguards against unrealistic expectations and overly burdensome procedures, as well as ensuring the consent process is informed and inclusive.<br>The two therapies (spironolactone and famciclovir) are considered to be safe, but it will be necessary to monitor their safety in this population over a prolonged period of time. Specific risks include hyperkalaemia, renal failure and gynaecomastia. Both drugs should be avoided in pregnancy and whilst breast feeding. |

## Glossary of Abbreviations, Terms, and Acronyms

| Abbreviation/Acronym | Definition |
|---|---|
| 6mCDP | 6-month Confirmed Disability Progression |
| 9-HPT | 9-Hole Peg Test |
| AE | Adverse Event |
| AI | Associate Investigator |
| CD8 | Cluster of Differentiation 8 |
| CDP | Confirmed Disability Progression |
| CI | Chief Investigator |
| CNS | Central Nervous System |
| CONSORT | Consolidated Standards of Reporting Trials |
| CTN | Clinical Trial Notification |
| CV | Curriculum Vitae |
| DMT | Disease Modifying Therapy |
| DNA | Deoxyribonucleic Acid |
| DSMB | Data Safety Monitoring Board |
| EBNA1 | Epstein-Barr Nuclear Antigen-1 |
| EBV | Epstein-Barr Virus |
| eCRF | Electronic Cade Report Form |
| EDSS | Expanded Disability Status Scale |
| eGFR | Estimated Glomerular Filtration Rate |
| EQ-5D-5L | EuroQol - 5 Domains – 5 Levels |
| EUC | Electrolytes, Urea and Creatinine |
| FBC | Full Blood Count |
| FIRMS-EBV | Fatigue In Relapsing Multiple Sclerosis – Epstein Barr Virus |
| FLAIR | Fluid Attenuated Inversion Recovery |
| FSMCF | Fatigue Scale Motor and Cognitive Functions |
| Gd | Gadolinium |
| HADS | Hospital Anxiety and Depression Scale |
| HLA | Human Leukocyte Antigen |
| HREC | Human Research Ethics Committee |
| ICH-GCP | International Conference on Harmonisation – Good Clinical Practice |
| IM | Infectious Mononucleosis |
| IMP | Investigational Medicinal Product |
| LFT | Liver Function Tests |
| LTFU | Lost To Follow Up |
| MAMS | Multi-Arm, Multi-Stage |
| MRI | Magnetic Resonance Imaging |
| MRFF | Medical Research Future Fund |
| MS | Multiple Sclerosis |
| MSFC | Multiple Sclerosis Functional Composite |
| MSIS-29 | Multiple Sclerosis Impact Scale-29 |
| MSWS-12 | Multiple Sclerosis Walking Scale-12 |
| NPS | Neuropathic Pain Scale |

| | |
|---|---|
| PCR | Polymerase Chain Reaction |
| PI | Principal Investigator |
| PICF | Participant Information and Consent Form |
| PIDN | Participant Identification Number |
| PLATYPUS | PLatform Adaptive Trial for remYelination and neuroProtection in mUltiple Sclerosis |
| PROM | Participant Reported Outcome Measure |
| pwMS | Person with Multiple Sclerosis |
| QALY | Quality Adjusted Life Year |
| RCN | Randomisation Code Number |
| SAE | Serious Adverse Event |
| SDMT | Symbol Digit Modalities Test |
| SOC | Standard Of Care |
| SOP | Standard Operating Procedure |
| T25FW | Timed 25-Foot Walk |
| TNF | Tumour Necrosis Factor |
| UK | United Kingdom |
| US | United States |

# 1. Background

## 1.1. The Problem

Multiple sclerosis (MS) is a complex autoimmune and neurodegenerative condition which manifests differently in individuals over time. Without treatment, for a staggering 70% of people diagnosed with MS, their symptoms will become increasingly disabling.[1] The commonest form of MS is relapsing remitting (90% at disease onset), where recurrent bouts of symptoms punctuate periods of relative normality.[2] After 10-30 years 60% of these cases will transition to secondary progressive MS, where deterioration occurs regardless of relapses. In around 10% of cases the disease is progressive from the outset, which is termed primary progressive MS. These latter two forms are collectively termed progressive MS. Progressive MS has been likened to terminal metastatic cancer, chronic kidney disease, and severe heart disease due to its substantial impact on an individuals' quality of life.[3] A person with progressive MS can experience many significant symptoms which can greatly impact their quality of life. These include issues with mobilisation and use of legs and/or arms, severe pain, bladder and bowel incontinence, sensory issues, balance issues, loss of vision, spasticity, and issues with cognition. These symptoms can lead to dependency on others for care, social isolation and increased mortality. As people transition from no disability to severe disability, the annual per-person cost for MS more than triples from $30,581 to $114,813, which highlights that disease progression accounts for much of Australia's healthcare expenditure for MS (~$1.75 billion each year). Progressive MS currently affects over 20,000 Australians, and 1.4 million individuals globally. For these people, slowing, halting, or even reversing disability is an urgent priority which – unfortunately – current therapies fail to address.

Dishearteningly, over 40 traditional phase II and phase III clinical trials evaluating putative therapies for progressive MS in the last three decades have yielded mostly negative or mixed results, or been constrained by the toxicity and side-effects of the tested drugs. Further, standard routes to develop new therapies take 10 to 20 years to reach the market – a length of time that people living with progressive MS simply do not have. Importantly, this is not just a generational problem: the total number of people with progressive MS may reduce over time due to improved access to anti-inflammatory therapies early on. However, 15-50% of the newly diagnosed will still experience disability progression, either because they do not respond to current treatments or because they fail to access them early.[4] There is an urgent need for an innovative and rapid new approach to identify and translate safe and effective drugs to treat progressive MS, now and in the future.

## 1.2. Comprehensive understanding of the cause of MS

MS is known to arise as the result of a combination of genetic and environmental factors. Certain genes including human leukocyte antigen (HLA)-DR1501 and over 200 other genetic loci are known to increase the risk of MS. Relative vitamin D deficiency, lack of sunlight prior to age 15, smoking, obesity and a diet high in saturated fat are all environmental factors that increase the risk of MS. A history of infectious mononucleosis (IM) is more common in people with MS than controls[5] and MS is associated with a later age of IM infection.[6] There is a near linear relationship between age of IM and age of onset of MS with a latency of 10-20 years.[7] Evidence of infection with Epstein-Barr virus (EBV), the cause of IM, is essentially universal in people with MS, compared to being seen in 90% of the general adult

population.[8] MS is associated with higher titres of antibodies to Epstein-Barr nuclear antigen-1 (EBNA1).[9] Recent studies have shown that MS only occurs in people who acquire EBV prior to the onset of their disease.[10] Thus, it appears that EBV is "essential, but not sufficient", to cause MS. This has led to the conclusion that EBV is the primary driver of MS pathology and disease activity. Many members of this experienced clinical trial team have been at the forefront of demonstrating the roles of genes, latitude, vitamin D, diet and EBV in the pathophysiology of MS. This understanding underpins the proposed clinical translation.

Many existing therapies for MS can be postulated to work by either removing EBV-infected B cells (ocrelizumab, alemtuzumab, teriflunomide, cladribine), blocking their entry into the central nervous system (CNS) (natalizumab), trapping them in lymphoid tissue (fingolimod, siponimod, ozanimod, ponesimod), or through upregulating anti-viral responses via γ-receptors (β-interferons). Recent studies of teriflunomide have demonstrated significant reductions in salivary EBV deoxyribonucleic acid (DNA)[11] and EBNA1 antibody titres[12] in people with MS. Importantly this study demonstrated that this effect was seen within 3-6 months of commencing teriflunomide. It is therefore logical to consider existing therapies with known efficacy against EBV as potential treatments for MS.

## 1.3. The Solution

Our project will harness the potential of removing or reducing the underlying cause of MS (EBV) and deliver on the need for treatments for progressive MS by driving innovation across two core domains of traditional MS research. Firstly, by adopting a pioneering multi-arm, multi-stage (MAMS), design for clinical trials that generates robust results more quickly and cheaply. Secondly, selecting drugs for testing and the trial's outcome measures in a rigorous and systematic way enhances confidence in positive trial outcomes and facilitates their rapid translation into practice. These innovations promise to transform the status quo of research and clinical care for progressive MS.

### *1.3.1. Innovative and informed approach to trial design*

We have formed a group of national and international experts in neurology, virology, immunology, EBV biology, pharmacology, statistics clinical trial design and health economics to optimise the chance of a successful trial. The proposed protocol has been reviewed by international experts in MS clinical trials. We have consulted with a group of people living with progressive MS on the trial design and as a consequence, this will be an "add-on trial" where existing proven therapies for progressive MS will be permitted as standard of care (SOC), ensuring that participants entering the study will not be denied existing proven therapies.

### *1.3.2. Rationale for adaptive trial design*

Despite the identified clear unmet clinical need for effective neuroprotection, which has been prioritised by consumer and professional groups, comparatively few clinical trials aim to modify the disease course of progressive MS. Novel approaches to evaluating multiple treatments concurrently, which incorporate adaptive elements such that they evolve over time to address the most current, relevant questions (sometimes termed 'platform' trial designs) have been highly successful in speeding up the evaluation of therapies in other

disease settings, such as the STAMPEDE trial in prostate cancer[13] and the RECOVERY trial for the treatments of COVID-19.[14] These have led to practice-changing advances.

MAMS adaptive platform designs offer flexible features, which can provide efficiencies at various levels, especially in a setting where there are numerous candidate drugs, which require evaluation. These include:

- simultaneous evaluation of multiple treatments against a common control arm (with efficiencies in terms of both time and the numbers of control participants)
- the ability to add new treatments as they become relevant, reducing the set-up time for new interventions, and dropping treatments that are not showing sufficient promise allowing redirection of resources

In Stage 1, participants are randomised as indicated to investigational product (IMP) or control arms and then each followed for 24 weeks with the primary outcome being measures of EBV activity. Once a participant has had their week 24 visit, they move into Stage 2, until the analysis of Stage 1 is reported. Then a decision will be made whether to continue a trial arm into Stage 2 follow-up or to terminate that arm. Once all participants have been recruited into Stage 1, further participants will be recruited into Stage 2, until at analysis a trial arm continues or is terminated. For Stage 2 clinical outcome measures of progression will be used.

The scientific integrity can be maintained as the overall hypothesis will be consistent through adaptations and the objectives unchanged, with arms being added and dropped on the basis of pre-specified criteria. Utilising an adaptive trial design has significant potential for delivering trials as a rolling programme. On an operational level, this maximises the use of infrastructure established at the start of the trial, thus reducing cost and set-up times, which would be associated with multiple individual trials and would further delay time to results. It also avoids issues of managing competing trials. As such, a MAMS design will provide a structure through which re-purposed and novel anti-viral drugs can be evaluated in a time- and cost-efficient manner in people with progressive MS.

### 1.4. EBV biology

EBV is a herpes virus that following acute infection (referred to as IM when this occurs in older children and adults), enters a latent phase within B cells that persists for life. It has been demonstrated that EBV-infected B cells can be found in the brains of people with MS[15] and that progressive MS is associated with enrichment of these cells in cortical lesions.[16] EBNA1 antibodies are also known to cross-react with a specific region of GlialCAM[17] and myelin,[18] leading to the generation of autoantibodies directly against these components of the CNS in people with MS. EBV gains access to human tissue via epithelial cell and B cell-specific antigens (gp350, gH/gL, gp42 and gB). The protein gp350 engages with complement receptor 2 on B cells to gain entry. Once EBV has infected B cells the virus goes through a cycle of phases but ultimately ends up in a latent state where additional antigens (including EBNA1) are produced. This latent phase assists EBV in evading the immune system indefinitely. It is known that people with MS have higher levels of antibodies to EBNA1, indicating that T cell responses (the normal mechanism for clearance of intracellular pathogens) may be suboptimal in people with MS. Elevated EBNA1 levels are associated with the risk genotype of several MS risk loci.[19] A significant proportion of MS risk loci exhibit a genotype-dependent expression pattern in EBV-infected B cells[20] and are enriched in the genome binding locations of the EBV-encoded transcription factor EBNA2.[21]

There is evidence that autoreactive, EBV-infected, B cells sequestered within lymphoid tissue within the CNS (pial surface of the grey matter in particular) are a primary driver of continuing low grade inflammation which leads to progressive disability in the absence of relapses in MS.[16] This appears to occur through a combination of slow-burn inflammation and failure of repair mechanisms, mediated via cytokines and chemo-attractants released from activated B cells that are immortalised as a result of latent EBV infection. Imagine then an intervention for progressive MS that targets the primary driver of continued CNS inflammation, degeneration and failure of repair. A number of existing anti-viral and other agents are known to have in vitro and clinical activity against EBV.[22] A key element to any putative therapy in this population will be CNS penetration. The blood-brain barrier is a multi-layered filtering system that normally protects the brain from the passage of large molecules and immune cells. For efficacy against the pial lymphoid tissue, high CNS and blood-brain barrier penetrance would be desirable.

Animal studies have established that HLA-DR15 (the genetic locus with the highest association with MS susceptibility) results in higher viral load.[23] In MS there is evidence for a relative deficiency of CD8+ T cells,[24] cells that are crucial in targeting intracellular pathogens such as EBV. Levels of EBNA1 antibodies correlate with disease activity in MS and appear up to 5 years prior to onset.[10]

A comprehensive review of MS and EBV biology was undertaken in 2022 by this research team (see Appendix 5).

### 1.5. Systematic approach to drug selection

We have conducted a peer-reviewed process for assessing putative anti-EBV therapies focusing on safety, CNS penetrance and evidence for efficacy against both the lytic and latent phases of the EBV life cycle. Using published reviews of putative agents, we have generated "drug CV's" (see Appendices 6 and 7) for potential candidates and reviewed with in our Drug Selection Committee which included international experts as well as a group of 6 people with MS. A scoring system incorporating clinical and pre-clinical evidence of efficacy against EBV and in MS, route of administration, brain penetrance, toxicity and tolerability (as judged by people with MS) was used to produce a shortlist of final candidates (manuscript in preparation – see Appendix 8).

The team are aware of drugs specifically targeting the latent phase of EBV (e.g. EBNA1 inhibitors), but none of these are at a stage of development suitable for evaluation in a phase III clinical trial.

### 1.6. Evidence-based outcome measures

Stage 1 will use EBNA1 antibody titres and frequency of salivary EBV DNA as the co-primary outcome measures. These have been determined to be the best measures of EBV activity.[25] The UK MS Society Expert Consortium for Progression in MS Clinical Trials has undertaken a systematic review of clinical trial design and outcome measures for progressive MS showing that confirmed disability progression (CDP) was the best clinical outcome measure[26] and this will serve as our Stage 2 primary outcome measure. CDP is determined from a validated clinical composite score, derived at 6-monthly intervals using Expanded Disability Status Scale (EDSS),[27] the Timed 25-Foot Walk (T25FW)[28] and the 9-Hole Peg Test (9-HPT).[29] These outcome measures were influenced by both our community

advisory group and prior reviews of progressive MS outcomes undertaken for the UK OCTOPUS trial (see Appendices 9 and 10). In addition, we will use a web-based application (MSReactor) as an exploratory outcome measure (https://msreactor.com/).[30] MSReactor is an Australian developed application that has shown promise as a more sensitive marker of both physical and cognitive impairment using a simple reaction time test which takes only minutes to perform. This will be an optional component of the trial.

***MS community engagement:*** - People with lived experience of MS have been involved in every stage of the trial design for this project via formal consumer advocacy pathways (please see Appendix 11). The research team includes two people with MS (one PI and one Associate Investigator (AI)). Our design meetings have been regularly attended by several people with MS and their input has been actively sought. As outlined above our drug selection committee included people with MS and a subcommittee of just people with MS was established to review tolerability profiles of the drugs being considered. Their input contributed 50% of the drug scores used to establish the final shortlist. People with MS were also consulted in the selection of outcome measures by our UK colleagues.

### 1.7. Impact

If either agent is effective in reducing the accrual of disability in progressive MS the use of such inexpensive and safe therapies could be transformative for the clinical care of people with progressive MS. In addition, confirmation of the role of EBV in progressive MS would pave the way for a wide range of therapeutic strategies targeting EBV in all forms of MS (e.g. anti-virals, vaccines, T cell therapy) that could drastically change the long-term prognosis for MS.

We propose to run this innovative adaptive MAMS phase III clinical trial to evaluate the effectiveness of promising therapies (spironolactone and famciclovir) which act against Epstein-Barr virus (EBV) in the treatment of progressive multiple sclerosis (MS). This study has been designed in active collaboration with people with MS and facilitates the prospect of rapidly and efficiently repurposing existing approved therapies for the treatment of MS.

The STOP-MS trial meets several aims:

- The STOP-MS trial can be rapidly translated to clinical care as the agents being tested are currently approved for use in Australia and elsewhere (for indications other than MS). We would aim to repurpose these agents for the treatment of MS.
- The STOP-MS trial will provide an opportunity for people with progressive MS to participate in a clinical trial designed specifically for them – this is an opportunity that has barely existed in the past.
- People with MS have been involved in all stages of planning for the STOP-MS, have actively contributed to the design and will be involved in the ongoing running and monitoring of the study.
- We have built upon established collaborations with trial design experts in the US and UK.

If successful the STOP-MS trial will bring a completely new class of therapeutic intervention (anti-EBV therapy) to address the single greatest unmet need for people with MS, namely effective treatments for progressive MS. Additionally, if anti-EBV therapy proves to be effective in progressive MS there is every likelihood that it would be effective in other forms of MS.

## 1.8. Proposed Trial

We propose a phase III, multicentre, randomised, double-blinded, placebo-controlled, MAMS trial of SpironolacTone and famciclOvir in the treatment of Progressive MS to prevent disability progression (STOP-MS).

Targeting progressive MS addresses the current single biggest unmet need in MS therapy and anti-EBV therapy potentially strikes at the root-cause of progressive disease (EBV-infected B cells in CNS lymphoid tissue).[16] The MAMS trial design aims to efficiently test putative anti-EBV therapies purely on their ability to reduce measures of EBV activity in stage 1 to determine the most likely clinically effective agent. The most effective therapy will then be tested in stage 2 using standard clinical measures of disability as the primary outcome measure. This protocol has been developed in accordance with the Consolidated Standards of Reporting Trials (CONSORT) checklist (Appendix 12).

The trial design has been developed by a large groups of Australian MS clinical and basic science researchers, including input from prominent international collaborators. A summary of the steps in developing this protocol is included in Appendix 13. The trial was also reviewed through the NHMRC granting process and was granted an MRFF grant (see Appendix 14).

***Drug selection:*** - From the final shortlist of tenofovir alafenamide, maribavir, famciclovir and spironolactone from our international expert panel review (see Appendix 15) we have chosen spironolactone and famciclovir for this study in people with progressive MS. Mirabavir and tenofovir alafenamide were excluded based on cost given the long duration of this study.

Spironolactone has been shown to reduce tumour necrosis factor-α (TNFα) levels of lipopolysaccharide-activated microglia by >50%.[31] Spironolactone inhibits EBV replication in the late lytic phase by blocking SM protein.[32] This occurs as a result of inhibition of xeroderma pigmentosum group B-complementing protein, a component of human transcription factor II H which EBV recruits in the transcription of several late lytic antigens.[33] In a case report spironolactone was effective in controlling EBV in a case of non-human immunodeficiency virus, acquired immunodeficiency syndrome.[34] In a pilot study of 9 people with MS (6 with progressive MS) a combination of spironolactone and aldosterone was effective in improving symptoms.[35]

Famciclovir is a prodrug of penciclovir which is then phosphorylated to the active metabolite penciclovir triphosphate. Penciclovir is phosphorylated by herpesvirus thymidine kinase, so this only occurs in herpesvirus infected cells. Penciclovir inhibits EBV replication in cell culture[36] and brain concentrations in rats were 41.5% of the concentration in muscle.[37] The related drug aciclovir has been used for the treatment of severe acute-EBV infection[38] and famciclovir has been successfully used to treat a case of overwhelming IM.[39] Randomised, double-blind, placebo-controlled trials of valaciclovir (the prodrug of aciclovir) have shown a reduction in the number of new active lesions on magnetic resonance imaging (MRI) in those with active disease,[40] and trends towards a reduction in annualised relapse rate and EDSS progression.[41] A randomised double-blind, placebo-controlled trial of aciclovir showed a trend towards reduction in annualised relapse rate which when dichotomised to low or high relapse rate became statistically significant.[42] We have chosen famciclovir on the basis of its greater bioavailability, higher intracellular concentration and greater persistence in infected cells.[43]

***Pharmacodynamic and Pharmacokinetic Data***

The key pharmacodynamic and pharmacokinetic properties of the two drugs are given in Table 1.

**Table 1. Pharmacodynamic Data for Spironolactone and Famciclovir**

| **Measure** | **Spironolactone** | **Famciclovir** |
|---|---|---|
| EBV $EC_{50}$ | | 1.5 µg/mL[44] (penciclovir) |
| EBV $IC_{50}$ | 0.87 µg/mL[32] (spironolactone)<br>1.16 µg/mL[32] (canrenone) | 5.1 µg/mL[36] (penciclovir) |
| $t_{max}$ | 2.6 hrs[45] (spironolactone)<br>4.3 hrs[45] (canrenone) | 0.9 hrs (penciclovir) |
| $C_{max}$ | | |
| 100 mg PO Daily | 0.21 µg/mL[45] (spironolactone)<br>0.43 µg/mL[46] (canrenone) | |
| 200 mg PO Daily | 0.67 µg/mL[47] (canrenone) | |
| 250 mg PO Daily | | 1.6 µg/mL (penciclovir) |
| 500 mg PO Daily | | 3.3 µg/mL (penciclovir) |
| Half-life | 12 hrs (canrenone) | 2.3 hrs (penciclovir) |
| Protein binding | 90% (canrenone) | <20% (penciclovir) |
| Excretion | 50% Urine, 15% Bile | Urinary |
| Molecular weight | 416.57 g/mol (spironolactone)<br>340.46 g/mol (canrenone) | 321.34 g/mol (famciclovir)<br>253.26 g/mol (penciclovir) |

Data from Australian Product Information Sheets or listed articles

[a] calculated from concentration resulting in >50% reduction of maximal viral replication = 2.5 µM

EBV = Epstein-Barr virus

EC50 = half maximal effective concentration

IC50 = half maximal inhibitory concentration

$t_{max}$ = time to maximum concentration

$C_{max}$ = maximum plasma concentration

*Spironolactone*

Spironolactone is rapidly converted to canrenone which is the primary active metabolite in terms of the mineralocorticoid effect. There are additional metabolites that also have mineralocorticoid effects. In terms of the anti-EBV effects of spironolactone, only spironolactone and canrenone have been studied. These data indicate that spironolactone itself has a slightly more potent effect against EBV than canrenone, although the difference is marginal.[32] Spironolactone causes degradation of xeroderma pigmentosum group B-

complementing protein (XPB) which is a component of human transcription factor TFIIH, in both B lymphocytes and epithelial cells. Depletion of XPB inhibits EBV SM protein. SM protein acts as a transcriptional activator for 15 late lytic genes that are essential for virion production. In vitro studies suggest that spironolactone and canrenone have $IC_{50}$ concentrations of around 1 μg/mL.[32] However, it should be noted that there are technical difficulties with measuring plasma levels of spironolactone.[45] The peak concentrations of spironolactone (0.21 μg/mL) and canrenone (0.43 μg/mL) after a single dose of 100 mg are are approximately 25% and 40% of their respective $IC_{50}$ concentrations.[32, 46] Some increase in peak concentration (around 30%) is typically seen with repeated daily dosing. Peak concentrations with 200 mg are higher. With a half-life of 12 hours for canrenone a similar effect could be expected with twice daily dosing of 50 mg (equivalent daily dosage).

*Famciclovir*

Famciclovir exerts its anti-EBV effects through the inhibition of EBV DNA synthesis. For famciclovir the $EC_{50}$ and $IC_{50}$ values for the active metabolite penciclovir are in the range of 1.5 – 5.1 μg/mL. The maximum concentration of penciclovir after 500 mg daily (steady state) of famciclovir (3.3 μg/mL) sits in the middle of this range. The half-life of penciclovir is 2.3 hours and a dose of 500 mg BD is likely to results in slightly higher peak concentrations.

***Clinical Data***

*Spironolactone*

Spironolactone was used to treat reactivation of EBV in an immunosuppressed person with MS and non-Hodgkin lymphoma (in addition to ganciclovir), but the dose used was not provided.[34]

Spironolactone 12.5 - 25 mg OD was used to treat EBV related fatigue in chronic fatigue syndrome.[48] This dose was not tolerated in 5/21 (24%), but this is a group that may be particularly sensitive to the hypotensive effects of spironolactone.

Doses of 25 – 200 mg per day have been used in young women to treat PCOS for 6-12 months and have been found to be safe.[49]

A dose-doubling analysis of the effects of spironolactone on various measures indicates the following mean changes in serum potassium levels:

25 mg Daily = +0.22 mmol/L

50 mg Daily = +0.35 mmol/L

100 mg Daily = +0.50 mmol/L

*Famciclovir*

Famciclovir at doses of 125 - 1000 mg per day have been found to be safe and well tolerated for durations up to 12 months.[50-53]

Famciclovir 500 mg tds was used to successfully treat severe IM in an immunocompetent person.

A recent study, which is yet to be peer reviewed, has shown that famciclovir 500 mg bd did not show a statistically significant difference in EBV salivary shedding or EBNA1 antibody titres, but the duration of this study was only 12 weeks.[54] This dose was not tolerated in 6/30 (29%) of cases.

The TGA approved dose for suppression of genital herpes in people with HIV is 500 mg bd and can be taken indefinitely. Several trials have demonstrated the efficacy and safety of famciclovir for the suppression of genital herpes in adult populations at doses ranging from 125 mg bd up to 500 mg bd for prolonged periods of time (up to one year).[1-3] A recent phase II clinical trial of famciclovir at a dose of 500 mg bd for 12 weeks in people with multiple sclerosis showed a reassuring safety profile.[4]

***Final Dosage Selection***

The proposed dose of spironolactone (50 mg BD) is likely to result in plasma levels that are in the order of 25-50% of the $IC_{50}$ for spironolactone and canrenone. It is likely the effective of these would be added (75% of $IC_{50}$) and other metabolites may also contribute additional anti-EBV effects. This dose has been well tolerated in other settings outside of the main indications for spironolactone. Therefore 50 mg BD represents the best compromise of potential anti-EBV efficacy and tolerability. The proposed dose of famciclovir (500 mg BD) results in plasma levels that are within the predicted $EC_{50}/IC_{50}$ range for penciclovir and therefore has the potential to be effective against EBV. This dose has been well tolerated in prior studies over long periods (up to 12 months) for current indications, although the same dose was less well tolerated in a recent cohort of people with MS. For both spironolactone and famciclovir we have proposed a protocol of commencing with half-dose therapy and then increasing to the full-dose only if well tolerated and all safety parameters are satisfactory. The inclusion of an option to remain on half-dose, or return to half-dose in the event of tolerability issues, provides some reassurance that tolerability can be optimised. This also reflects would likely be any future real-world use of these agents.

***Add-on Study:*** - A clear message from our Consumer and Community Reference Committee was the need to ensure that the study would be open to as many people with progressive MS as possible and that enrolment should not be precluded by being on any existing MS therapy. The control arm has therefore been designed to ensure that participants will still have access to appropriate therapies currently available for progressive MS; current SOC. Thus, the control arm will be SOC plus placebo and the active arms will be SOC plus spironolactone or famciclovir.

## 1.9. Regulatory Approval

### *1.9.1. Trial Registration*

The STOP-MS trial has a World Health Organisation, Unique Trial Number (U1111-1293-1787) and has been registered with the Australia and New Zealand Clinical Trial Registry

(ANZCTN12623000849695p). A copy of this protocol is available at: https://www.anzctr.org.au/Trial/Registration/TrialReview.aspx?id=386167&isReview=true

The trial has been notified under the Clinical Trial Notification (CTN) scheme with the Therapeutic Goods Administration (CT-2023-CTN-03505-1-v1).

### *1.9.2. Human Research Ethics Committee Approval*

Ethics approval has been granted from the lead Human Research Ethics Committee (HREC), Gold Coast Health (HREC/2023/QGC/101052). Additional HREC approval will be sought for sites that are not included in the National Mutual Acceptance scheme.

### *1.9.3. Governance approval*

Site Specific Approval (SSA) will be sought at each participating site either through the ERM system directly or via separate applications.

# 2. Study Objectives

## 2.1. Research Question and Aims/Objectives

### *2.1.1. Primary Aims/Objectives*

#### *2.1.1.1. Stage 1*

The primary aim of stage 1 of this trial will be to demonstrate that spironolactone or famciclovir plus SOC reduce the frequency of EBV DNA being present in saliva and/or reduce EBNA1 antibody titres in people with progressive MS when compared to placebo plus SOC.

#### *2.1.1.2. Stage 2*

The primary aim of stage 2 will be to demonstrate that spironolactone or famciclovir plus SOC reduce the likelihood of 6-month CDP (6mCDP) in people with progressive MS when compared to placebo plus SOC.

### *2.1.2. Secondary Aims/Objectives*

The secondary aims will be to demonstrate that spironolactone or famciclovir plus SOC:

- Are safe when used to treat people with progressive MS.
- Reduce the rate of brain atrophy at 3 years compared to placebo plus SOC.
- Reduce the numbers of new/expanded T2/FLAIR and Gadolinium (Gd)-enhancing lesions on MRI brain compared to placebo plus SOC.
- Reduce the level of whole brain atrophy on MRI brain compared to placebo plus SOC.
- Improve participant-reported outcome measures (PROMs) of disease impact compared to placebo plus SOC.
- Are cost-effective.

## 2.2. Hypotheses

The hypotheses that the STOP-MS trial aim to address are:

1. Spironolactone or famciclovir plus SOC will reduce the frequency of salivary EBV shedding or EBNA1 antibody titres in people with progressive MS when compared to placebo plus SOC.
2. Spironolactone or famciclovir plus SOC will reduce the likelihood of 6mCDP in people with progressive MS when compared to placebo plus SOC.
3. Spironolactone or famciclovir plus SOC will be associated with a similar rate of adverse events compared to placebo plus SOC when used in people with progressive MS.

4. Spironolactone or famciclovir plus SOC will reduce the numbers of new/expanded T2/FLAIR and Gd-enhancing lesions on MRI brain when compared to placebo plus SOC (this hypothesis is subject to additional funding).
5. Spironolactone or famciclovir plus SOC will be associated with reduced whole brain atrophy on MRI brain when compared to placebo plus SOC.
6. Spironolactone or famciclovir plus SOC will be associated with improvement in PROMs when compared to placebo plus SOC.
7. Spironolactone or famciclovir will be cost effective in reducing disability accrual in progressive MS.

# 3. Methods

## 3.1. Methodological Approach

STOP-MS will be a MAMS trial designed to yield effective therapies for progressive MS as quickly as possible. It will be a phase III clinical trial of spironolactone and famciclovir against placebo in preventing disability progression in MS. The study will be conducted in two stages. In stage 1, which will last 6 months, the co-primary outcome measures will be presence of salivary EBV DNA and serum ENBA1 antibody titres. In stage 2, provided minimum criteria for evidence of anti-EBV efficacy, the best performing agent from stage 1 will be translated into a single treatment arm study against placebo with 6mCDP being the primary outcome measure. A summary of the study design is given pictorially in Figure 1.

**Figure 1. Summary of Study Design**

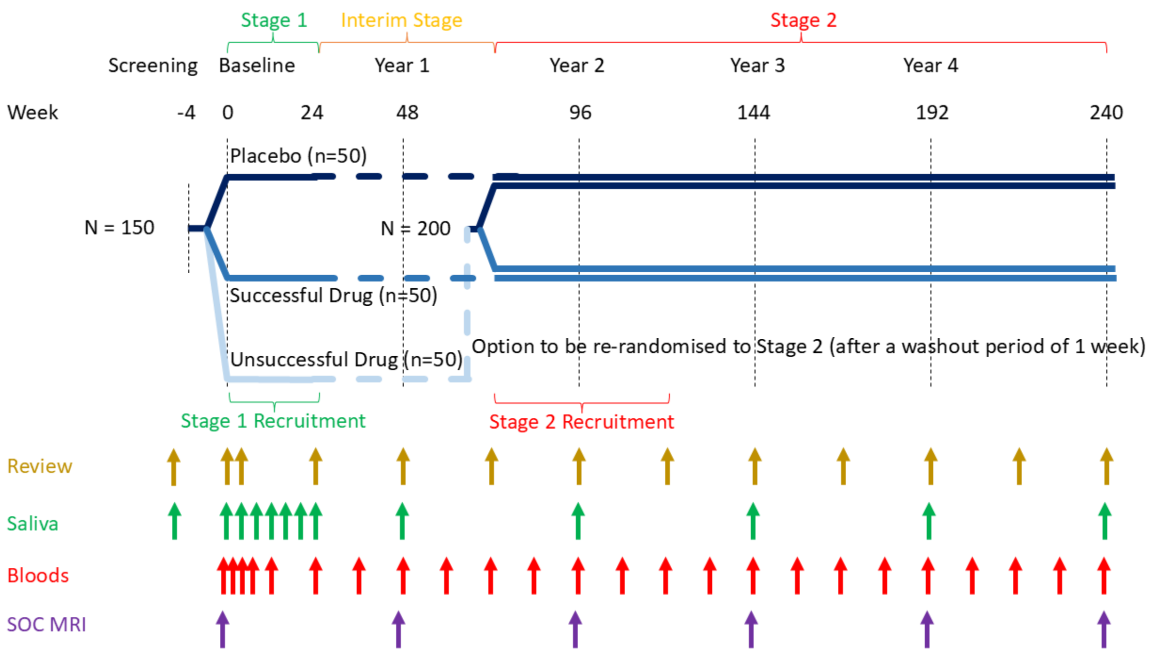


## 3.2. Study Sites/Settings

A total of 22 participating clinical sites across five states of Australia will participate in this study (Griffith University, Royal Brisbane and Women's Hospital, Princess Alexandra Hospital, Mater Hospital Brisbane, Sunshine Coast Hospital, John Hunter Hospital, Royal North Shore Hospital, Brain and Mind Centre, Westmead Hospital, Liverpool Hospital, Concord Hospital (Sydney), Monash Medical Centre, Alfred Hospital, Austin Hospital, Royal Melbourne Hospital, Box Hill Hospital, Royal Adelaide Hospital, Flinders Medical Centre, Menzies Institute for Medical Research, University of Tasmania, Launceston General Hospital, Perron Institute). Each site would be expected to recruit 5-10 participants over 6 months in stage 1 and 5-15 participants over 12 months in stage 2. These sites all have extensive experience of investigator-initiated and commercially sponsored clinical trials (this

team worked together on the PREVANZ trial [ACTRN12612001160820] – the largest clinical trial of vitamin D in MS). We have chosen a per patient funding model to most efficiently reflect the actual costs for sites and to facilitate recruitment.

### 3.3. Study Population

Potential participants with progressive MS will be recruited from MS Clinics at the participating sites. Inclusion criteria will only be considered at the time of enrolment for the purposes of determining eligibility to enter the trial. Any subsequent change in any relevant parameters after enrolment and randomisation (e.g. age advancing beyond 70 years or EDSS moving outside of 4.0 – 8.0) will not be considered as a reason for exclusion/withdrawal from the trial.

The following inclusion and exclusion criteria will be applied:

#### *3.3.1. Inclusion criteria*

- Age 25-70 years (inclusive)
- Diagnosed with primary or secondary progressive MS according to McDonald 2017 criteria[55]
- EDSS[27] of 4.0 – 8.0 (inclusive) at the time of randomisation
- Evidence of disability progression over the previous 24 months
- English speaking or non-English speaking but can ensure external interpreter assistance (e.g. relative or friend) to attend all visits for the duration of the clinical trial
- Available to attend clinic visits

#### *3.3.2. Exclusion criteria*

- A clinical relapse within 3 months of randomisation
- A significant co-morbidity that in the opinion of the principal investigator (PI) would negatively affect MS disease outcomes or preclude administration of spironolactone or famciclovir (including renal failure; estimated glomerular filtration rate < 30ml/min, significantly abnormal ECG result, or significant abnormality in pathology screening)
- Currently taking medication or supplements known to cause hyperkalaemia as listed in Appendix 16
- Hypersensitivity to spironolactone or famciclovir
- Female participants who are pregnant
- Female participants who are breast-feeding
- Women of childbearing potential who are unwilling or unable to use an acceptable method of contraception (see Appendix 17) whilst on trial treatment and for up to 30 days after the last dose of study drug
- Have received treatment with steroids (intravenous and/or oral) for MS relapse/progression within 3 months before randomisation

- Have received any trial therapy within the last 6 months (other than as part of the STOP-MS Stage 1 trial)
- Recent or current history of major depression, bipolar disorder, psychosis or suicidality
- Currently or recently taking any illicit substances (excluding cannabis products used for symptomatic relief)

### 3.4. Recruitment/Selection

Potential participants will be recruited through the MS or CNS inflammatory disease clinics of the 22 participating hospitals with a cap of 40 participants at any single centre. Participants may be identified by neurologists at each site and referred directly to the local trial team for screening (after providing consent to be contacted). Participants may also register initial interest by completing a pre-screening questionnaire through MS TrialScreen. This is an online portal operated through the University of Tasmania which serves as a patient facing pre-screening tool for multiple MS trials including STOP-MS (HREC approval granted through University of Tasmania Human Research Ethics Committee (HREC) on 18/12/2024 Project ID: 31237).

Potential participants will be identified by neurologists at each site, who will provide an introduction and overview of the study and an invitation to review more detailed information. Those potential participants who are interested will be provided with the Participant Information and Consent Form (PICF) to take away and read, and a further screening visit will be arranged at a later date when the participant will have the opportunity to ask questions and have all queries answered by the investigators, before indicating their understanding and providing written consent.

Participants will be enrolled in the study at the screening visit provided they:

- Have given written informed consent
- Meet all the inclusion criteria
- Meet none of the exclusion criteria
  Participants will not be financially compensated for their involvement in this trial but will be reimbursed for any reasonable costs of participation (e.g. travel and parking costs).

#### *3.4.1. Aboriginal and Torres Strait Islander Peoples*

Owing to the rarity of MS in Aboriginal and Torres Strait Islander Peoples, it is considered unlikely that anyone of these populations is likely to be recruited to the STOP-MS trial. However, if this were to eventuate, the following will be implemented to ensure culturally safe practices:

(i) All site staff will be required to be up to date with all locally mandated cultural safety training.

(ii) Local Aboriginal and Torres Strait Islander Health Support Services will be approached for guidance and support.

#### *3.4.2. Randomisation*

***Stage 1:*** Participants will be randomised at enrolment to treatment with spironolactone, famciclovir or placebo at a 1:1:1 ratio. Randomisation will be performed using the randomisation module of the REDCap® database which generates a randomisation code (4-digit number) from the randomisation sheet produced in Excel®, Microsoft (Seattle, CA, USA) which is stratified by sex, age and site. This randomisation sheet will be generated by Assoc/Prof Jing Sun (statistician). There will a cap of 30 participants for any single site in Stage 1. The randomisation code is used to determine treatment allocation (randomly assigned in REDCap® using blocks of 3) and the treatment allocation then shown on a form in REDCap® that is only accessible by site pharmacists.

***Stage 2:***

Scenario 1: Assuming one treatment arm fails to meet the primary endpoints of Stage 1 and one treatment arm meets the primary endpoints of Stage 1: One treatment arm will be terminated and one treatment arm will be retained in Stage 2. The participants in the terminated arm will be offered the opportunity to be re-enrolled (new PIDN) and re-randomised into the remaining two arms.

Participants will be randomised to either the successful treatment arm from stage 1 (spironolactone or famciclovir) or placebo at a 1:1 ratio. The same randomization process as above will be utilised but using a second randomisation schedule with just two treatment allocation options (treatment or placebo) and blocks of two. There will be a cap of 40 participants for any single site in phase 2. Randomisation procedures will all be performed by an unblinded monitor under the supervision of the unblinded statistician.

Scenario 2B: Both treatment arms fail to meet the primary endpoint of Stage 1: Both treatment arms will be terminated and new treatment arm or arms will be added, to re-commence Stage 1.

Scenario 2C: Both treatment arms meet the primary endpoints of Stage 1: Both treatment arms will be retained in Stage 2. Study time will not be reset; the study baseline will remain at the start of Stage 1 for all participants.

#### *3.4.3. Blinding and allocation concealment*

This will be a double-blinded study with both patient and treating physician blinded as to whether the participant is receiving active treatment or placebo. At the screening visit, participants will be assigned a unique Participant Identification Number (PIDN). The PIDN will consist of the participants three initials (using X for middle initial if no middle name), the site number and a unique number assigned by the REDCap® database. At the baseline/enrolment visit, after eligibility is confirmed and the full consent form has been signed, the PIDNs will be randomly assigned to intervention or placebo groups in REDCap® by the randomisation module. Treatment allocation codes will only be visible through REDCap® to site pharmacists, who will then provide active treatment or placebo.

To maintain study blinding, all tablets will be over-encapsulated, so they all look the same.

#### *3.4.4. Labelling of IMP*

The IMP will be delivered from the manufacturer with both a permanent blinded label indicating the trial details, batch numbers, dose (half strength or full strength) and expiry

date, and a detachable unblinded label indicating the specific IMP and dose (in mg). At the screening visit participants will be randomly assigned an PIDN once the patient details have been entered into the REDCap database, this code will be added to the patients prescription which will then be presented to the site unblinded pharmacist. The unblinded pharmacist will use this code to determine treatment allocation within the REDCap database. The IMP allocation details will be visible within the Pharmacy Details form, which is only visible to pharmacists and unblinded monitoring staff.

The on-site pharmacist will select the appropriate IMP, according to the randomization provided in REDCap, and add the participants identifying details (PIDN, name). The detachable unblinded label will be removed and attached to the prescription request for that participant and kept with trial pharmacy records.

#### *3.4.5. Breaking of study blinding*

The randomisation IMP allocation for an individual participant may only be unblinded in emergency situations, where the Local Principal Investigator (or delegate) decides a participant cannot be adequately treated without knowing the identity of their treatment allocation. Usually this will be an emergency situation, but requests can arise in any clinical scenario. Such circumstances might include the urgent use of a lifesaving medication where interaction with one of the investigational medicinal products (IMP) is known to carry a risk of inducing an adverse outcome.

Non-urgent requests for unblinding can be made by email to stop-ms@griffith.edu.au. During office hours, the Local Principal Investigator (PI) can be contacted using the telephone number provided on the IMP label and on the participant identification card, or the Trial Monitor can be contacted on 07 5678 0546. Out of hours, or where the above contacts are unavailable, the Coordinating Principal Investigator (Professor Simon Broadley) can be contacted on 0466 207 444. If Professor Broadley is unable to be contacted, then Professor Jeannette Lechner-Scott can be contacted on 0402 964 260.

The Local Principal Investigator (or delegate) may contact the site pharmacy to obtain the treatment identity. If possible, such emergencies should be discussed with the Coordinating Principal Investigator before breaking the blind, however, the ultimate decision to unblind participant treatment allocation rests with the local Principal Investigator (or delegate). If the blind is broken for a participant, the time, date, participant number and reason for unblinding must be clearly documented.

Additional information regarding unblinding processes can be found if needed in the STOP-MS Pharmacy Manual or the STOP-MS-SOP-011 Unblinding of Investigational Product.

#### *3.4.6. On completion of the study*

Unblinded study data will only be available once all data collected have been entered into the electronic Case Report Form (eCRF) for every participant and the database has been finalized (locked), except in the case of an emergency, as detailed above.

### 3.5. Consent

The PICF provides details of the clinical trial and the expectations for both the participant and those conducting the trial. In particular, the PICF makes it clear that participation is voluntary and that participants can withdraw at any time. It is also specified that any decision to participate or not, will in no way affect subsequent MS care at the treating facility for the potential participant.

At the screening visit prior to any study-related activity, the site investigator will review the details of the study, assess the potential participants understanding of the information in the PICF and having asked the potential participant if they have any questions, answer those questions. Having established that the potential participant is fully informed and wishes to proceed in the study, the participant will provide written informed consent using the PICF. Study staff will ensure that an adequate explanation is provided to the participant and their family about the aims, the requirements of the study that need to be adhered to strictly, and any potential known and unknown risks and benefits of the study. Study staff will ensure that all questions about the study are answered adequately, and that the participant understands the information provided about the study. The study staff conducting the informed consent discussion will ensure that consent is voluntary and free from coercion. The staff member that conducts the consent discussion will also sign the informed consent form. A copy of the PICF will be provided to the participant to keep.

Consent will be specific for this study only.

### 3.6. Risk Mitigation Procedures

There are five principal risks of physical harm for participants in the proposed trial:

1. Risk of death from hyperkalaemia - spironolactone can cause hyperkalaemia (see Appendix 18). This principally occurs in three settings:

a) A pre-existing medical condition known to cause hyperkalaemia (chronic kidney disease, uncontrolled diabetes, congestive cardiac failure, hyperaldosteronism, congenital adrenal hyperplasia, Addison's disease, parathyroidectomy).

b) Renal impairment with an eGFR <30 ml/min.

c) Concomitant use of medications/supplements known to cause hyperkalaemia (carenoate potassium, eperenone, dropirenone, captropril, enalapril, lisinopril, candesartan, losartan, ibuprofen, naproxen, diclofenac, meloxicam, enoxaparin, amiloride, triamterene, trimethoprim, pentamidine, propranolol, atenolol, metoprolol, bisoprolol, digoxin, lithium, potassium chloride, epoetin alfa, epoetin beta, alfalfa, dandelion, horsetail, Lily of the Valley, milkweed, nettle, muscle-building supplements, salt-substitutes, diets high in potassium – for a full list of medications and supplements that can cause hyperkalaemia with trade names see Appendix 16).

2. Coincidental development of renal impairment causing:

a) Hyperkalaemia with spironolactone.

b) Confusion and drowsiness with famciclovir.

3. Development of gynaecomastia in males taking spironolactone, the risk of which may be related to dose and duration of therapy (see Appendix 19)

4. Spironolactone is a diuretic and therefore worsen or bring out for the first time symptoms of bladder dysfunction in people with MS. This might include increase frequency, nocturia, urgency and incontinence.

5. Spironolactone is potentially harmful to the foetus in pregnancy (category B3) and to the child during breast feeding. Famciclovir has been shown to be safe in animals during pregnancy and lactation but there is insufficient data in humans.

We propose the following mitigation strategies for these risks:

A. To reduce the risk of hyperkalaemia:

a) All PIs and delegates will be required to undergo training with regards to clinical impacts of hyperkalaemia, this will include (but is not limited to), causes of hyperkalaemia, clinical consequences of hyperkalaemia, ECG changes of hyperkalaemia (see Appendix 20), acute management of hyperkalaemia (see Appendix 21) and monitoring for renal impairment.

In brief the treatment of hyperkalaemia will be as follows:

(i) Serum potassium of 5.0 -6.0 mmol/l – urgent ECG to look for signs of hyperkalaemia – if present treat as for (ii) below; repeat potassium level and check renal function (creatinine and eGFR); check for acidosis; full blood count to exclude haemolysis; remove all agents causing hyperkalaemia; remove potentially cardiotoxic agents (e.g. digoxin, lithium); treat any identified underlying cause of hyperkalaemia; ensure adequate hydration; Resonium A if hyperkalaemia confirmed (oral or PR).

(ii) Confirmed serum potassium >6.0 mmol/l or if ECG changes present – in addition to the above, IV calcium gluconate (stabilises cardiac membranes); IV insulin and glucose; consider nebulised salbutamol, sodium bicarbonate if acidotic, frusemide if fluid overloaded, intravenous calcium chloride (via central line) and dialysis. This level of care should be provided in a suitable environment (e.g. emergency department, high dependency unit or intensive care unit).

b) All the conditions in 1 a) and drugs/supplements in 1 c) above will be exclusion criteria for participation in the trial.

c) Participants will be provided at enrolment with a laminated card indicating the list of drugs and supplements in 1 c) above that should be avoided for the duration of the trial (see Appendix 22).

d) Participants will be advised to indicate their involvement in the trial when being prescribed any new medications and in particular, if being prescribed antibiotics (trimethoprim) or pain medication (NSAIDs), and if purchasing NSAIDs over the counter (see Appendix 23 and Appendix 22).

e) Participants will be recommended to check the list of complimentary medicines and supplements (see Appendix 22) when choosing to start any such products and consult with site staff if in doubt.

f) All participants will undergo an ECG at the initial screening visit and ECG features of hyperkalaemia (peaked T waves, widening/flattening of P waves, PR prolongation, bradyarrhythmia, conduction block, QRS widening) or an increased risk of death from

hyperkalaemia (sinus bradycardia, AV block, slow junctional rhythm, slow AF, bundle branch block, fascicular block) are exclusion criteria to participation in the trial.

g) All participants will undergo baseline testing for serum potassium level and a level >5.0 mmol/L is an exclusion criteria for participation in the trial.

h) All participants will undergo baseline testing of eGFR and a level <30 ml/min is an exclusion criteria for participation in the trial.

i) All participants will undergo testing for serum potassium and eGFR at weeks 1, 2, 6 and 12 during treatment initiation and dose escalation phases and every 12 weeks for the duration of the trial, a potassium level >5.0 mmol/L or eGFR <30 ml/min if confirmed to not be a spurious result will be actively managed and will result in withdrawal of the participant from the trial, except where remediable alternative explanation can be identified.

j) A serum potassium level >5.0 mmol/L will be managed with an urgent ECG to check for signs of hyperkalaemia and a repeat serum level. If confirmed, the hyperkalaemia will be managed as per recommended protocols.

k) An eGFR <30 ml/min will result in a repeat level, and if confirmed as <30 ml/min will result in withdrawal from the trial.

l) Persistently rising serum potassium levels (still within normal limits) and falling eGFR (>30 ml/min) will be managed as deemed appropriate by the treating physician through any combination of: removal/avoidance of potential precipitants; more frequent testing; reduction to half-dose treatment; and/or withdrawal from the study.

m) If for medical or other reasons a participant is required to commence on one of the contraindicated medications, one of the following outcomes will be determined by the treating neurologist in consultation with the participant and advice from any relevant treating specialist:

(i) Cease IMP.
(ii) Reduce to half-dose IMP with close monitoring of potassium levels (2, 4, 8 and 12 weeks after introduction of contraindicated medication).
(iii) Continue IMP at full dose with close monitoring of potassium levels (2, 4, 8 and 12 weeks after introduction of contraindicated medication).

If IMP is ceased the participant would be encouraged to continue in the study and undertake the annual outcome assessments, but they will be offered the opportunity to withdraw if they so choose.

B. To reduce the potential impacts of renal impairment.

a) An eGFR <30 ml/min at screening is an exclusion criteria for the trial.

b) A confirmed (confirmed at repeat testing) eGFR <30 ml/min at any time point in the trial will result in withdrawal of the participant.

c) Falling eGFR (>30 ml/min) will be managed as deemed appropriate by the treating physician through any combination of: removal/avoidance of potential precipitants; more frequent testing; reduction to half-dose treatment; and/or withdrawal from the study.

d) Any adverse events of confusion, drowsiness will be investigated with an urgent check of renal function and electrolytes.

C. To reduce the risk of gynaecomastia:

Gynaecomastia is a recognised adverse effect of spironolactone and we are not aware of any strategies to reduce this risk. Our approach has been to consider the risks and review these with our consumer advisory group.

a) Our panel of people with MS were reassured by the fact that this complication of spironolactone therapy is usually reversible on withdrawal of the treatment.

b) The dose of spironolactone chosen is a compromise between the dose anticipated to have an adequate anti-EBV effect (based on animal studies) and dose that has a lower risk of gynaecomastia. An analysis of dose response curves and the effect of duration of therapy from a published meta-analysis[56] suggests a peak incidence rate of around 10% of men at 3 years for the 100 mg per day dose planned for this current trial (see Appendix 24).

c) All PIs will be trained in the clinical assessment of gynaecomastia, with a recommendation for immediate cessation of the trial medication in the case of gynaecomastia being detected.

b) MS predominantly affects women, with a sex ratio of 4:1 (females:males), although this may be closer to 3:1 for progressive forms of MS. In Stage 1 a total of 50 people with MS will be treated with spironolactone. Approximately 10-15 will be male. From the above estimate of risk, we might expect to see 1 case of gynaecomastia in Stage 1. If spironolactone is the drug chosen to move into Stage 2 of the trial 150 people with MS will receive this drug. Therefore the total number of males that may be exposed to spironolactone would be 30-45, meaning that approximately 3-5 cases of gynaecomastia might occur.

D. Participants will be warned of the risks of increased urinary symptoms and asked to report these. Severe symptoms may require an adjustment of dosage or withdrawal from the study.

E. To reduce the potential risks of pregnancy or breast feeding we propose the following:

a) Pregnancy and current breast feeding will be exclusion criteria for participation in the trial.

b) Women of childbearing potential will undergo a pregnancy test (serum βHCG) at screening and will be excluded if this is positive.

c) Women of childbearing potential will be required to agree to using an acceptable form of contraception for the duration of trial and for a period of 30 days after ceasing any IMP. Unwillingness or inability to comply with this will be an exclusion criteria to participation in the trial.

There is no evidence that spironolactone or famciclovir taken by males is associated with any risk to a foetus conceived by them.[57] Therefore, only women of child-bearing age will be required to use contraception during the STOP-MS trial.

A list of currently approved therapies for MS and other commonly prescribed symptomatic therapies, and their potential interactions with spironolactone and famciclovir is given in Table 1 (source MIMS Online). Neither spironolactone or famciclovir have an effect on cytochrome P450 isoenzymes.

Spironolactone may interfere with the elimination of digoxin leading to potentially increased toxicity.[58] Spironolactone may also interfere with digoxin assays making interpretation of serum levels.[58] Digoxin has therefore been included on the list of contraindicated drugs.

In addition to the physical harms listed above, the following ethical risks have been considered:

1. People with progressive MS can be in a state of desperation, having tried available therapies and yet still progressing or not being eligible for existing therapies. In addition, the prospect of a new “clinical trial” might be seen as offering more than it can realistically deliver.

**Table 1**

| MS Therapy – generic (trade names) | Spironolactone | Famciclovir |
|---|---|---|
| β-interferon 1B (Betaferon) | – | – |
| Peginterferon-β 1A (Plegridy) | – | – |
| Glatiramer acetate (Copaxone) | – | – |
| Teriflunomide (Aubagio) | – | – |
| Dimethyl fumarate (Tecfidera) | – | – |
| Diroximel fumarate (Vumerity) | – | – |
| Fingolimod (Gilenya) | – | – |
| Siponimod (Mayzent) | – | – |
| Ozanimod (Zeposia) | – | – |
| Cladribine (Mavenclad) | – | – |
| Natalizumab (Tysabri) | – | – |
| Ocrelizumab (Ocrevus) | – | – |
| Ofatumumab (Kesimpta) | – | – |
| Alemtuzumab (Lemtrada) | – | – |
| Oxybutynin (Ditropan, Oxytrol) | – | – |
| Solifenacin (Vesicare) | – | – |
| Mirabegron (Betmiga) | – | – |
| Sildenafil (Viagra) | – | – |
| Amitriptyline (Endep) | – | – |
| Duloxetine (Dytrex, Duloxecor, Tixol, Cymbolta) | – | – |
| Citalopram (Celapram, Talam, Cipramil) | – | – |
| Baclofen (Clofen, Lioresal, Stelax) | – | – |
| Clonazepam (Rivotril, Paxam) | – | – |
| Modafinil (Modafin, Modavigil) | – | – |
| CBD Oil (Sativex) | – | – |
| Gabapentin (Neurontin, Gabacor, Nupentin) | – | – |
| Pregabalin (Lyrica, Lypralin, Lyzalon, Neuroccord) | – | – |
| Carbamazepine (Tegretol | – | – |
| Famridine (Fampyra) | – | – |
| 4-aminopyridine | – | – |

“-” = no interaction

“+” = interaction

In view of this, site PIs will carefully counsel potential participants with regards to the experimental nature of the STOP-MS trial and ensure that their expectations are realistic. It will be important to ascertain that potential participants are not waiving opportunities to access therapies of known potential benefit based on their preference to enter into a clinical trial. Participants already taking any existing proven SOC therapy for MS, will be permitted to participate in the STOP-MS trial. Whilst it would be preferred that this therapy not be

changed for the duration of the trial, a change of therapy to any other approved SOC therapy for MS will be permitted.

2. People with progressive MS are, by definition, significantly affected in their activities of daily living to a lesser or greater degree. This may add additional burdens upon participants.

Site PIs will discuss fully with participants the specific issues that they may face through enrolment in the STOP-MS clinical trial (e.g. need to travel for regular visits and assessments).

3. MS rarely causes cognitive impairment to such a degree that capacity to provide informed consent is affected. However, those with progressive MS are most at risk of this.

Site PIs will carefully assess capacity according to recommended criteria. Namely that the potential participant understands the nature of the trial, what alternatives might be available, that they can withdraw at any time, what the potential side effects of the investigational products are, what the practical requirement for participation are, and that they do not have any significant mental health issues.

## 3.7. Participant Withdrawal Procedures

### *3.7.1. Screen Failure*

Participants who do not meet the requirements for enrolment will be deemed to be screen failures. As such they will not be enrolled in the study and will not be randomised to any study drug. They will be given a unique trial ID and listed in the enrolment log as a screen failure. A minimal set of information (age, sex, date, reason for screen failure) will be recorded in the eCRF in order to meet the CONSORT publishing requirements,[59] respond to queries from regulatory authorities and to ensure transparent reporting of screen failure participants.

### *3.7.2. Lost to Follow Up*

Investigational staff will go to all reasonable lengths possible to minimise loss to follow-up (LTFU) of participants by active and continued follow-up of participants who fail to attend study visits or who are otherwise not contactable. A participant will not be considered LTFU until all routes of contact have been exhausted. If a previously lost participant is retrieved within 2 weeks of the relevant study visit, they will be brought into the relevant study visit as soon as possible for the relevant measures such as primary endpoint determination. If a participant wishes to withdraw due to study burden, the trial coordinator and site nurse will make reasonable attempts to reduce trial duties for the participant, with a focus on continued collection of data relating to the primary outcome. If the participant wishes to withdraw for health reasons (such as intolerable side effects), the final assessment visit will be brought forward.

### *3.7.3. Stopping Rules*

Participants may withdraw from the study at any time for any reason with no impact on any future care they may require from the recruitment site or study staff. Participants may also

choose to cease the study treatment at any time but remain in the trial with continued follow-up until the end of the study; this will have no impact on any future care they may require in the participating clinics. If the participant experiences a serious adverse event (SAE) that is suspected to be linked to the study medication or compromises the participant's ability to adhere to the intervention, the intervention will be ceased. However, the participant can remain in the trial and will be followed-up through to the end of the study. In the event of a participant withdrawing from the trial an end of study visit should be organised if possible. The participant should be asked to sign the withdrawal of consent section of the PICF and should be asked to indicate their preferences in terms of data and biobank samples collected to that point as included on the withdrawal of consent form. If they are happy to, participants should also be asked to indicate the reason for withdrawal but they are not obliged to do so.

## 3.8. Study Procedures

### *3.8.1. Treatment arms*

Stage 1

- Arm 1: Spironolactone + SOC
- Arm 2: Famciclovir + SOC
- Arm 3: Placebo + SOC

Stage 2

- Arm 1: Successful active drug + SOC
- Arm 2: Placebo + SOC

#### *3.8.1.1. Intervention Description, Dosage and Route of Administration*

***SOC plus Spironolactone:*** - Spironolactone 50 mg twice daily administered orally.

Spironolactone is approved for the treatment of essential hypertension, congestive heart failure, cirrhotic liver disease and nephrotic syndrome. It is contraindicated in renal insufficiency, hyperkalaemia and pregnancy. Agents that can cause hyperkalaemia should not be used in conjunction with spironolactone. Adverse events include gynaecomastia which is usually reversible on discontinuation, gastrointestinal upset, drowsiness and allergic reactions (see Appendix 18). For these reasons we have chosen a medium dose of spironolactone, screen carefully as outlined above and monitor throughout the study as outlined below. Dose escalation will also be used to minimise potential adverse events (see below).

***SOC plus Famciclovir:*** - Famciclovir 500 mg twice daily administered orally.

Famciclovir is a synthetic nucleoside analogue and is approved for the treatment and prevention of herpes zoster and herpes simplex infections (shingles, genital herpes, herpes labialis and herpes encephalitis). Common adverse effects include headache and nausea. Renal impairment is a contraindication (see Appendix 19). We have chosen the chronic treatment dose to ensure adequate CNS penetrance and participants will be screened for renal failure before proceeding to study randomisation.

***SOC plus Placebo:*** - Matched placebo capsules twice daily administered orally.

##### *3.8.1.2. Blinding*

The two investigational products and placebo will be manufactured as over-encapsulated capsules that will be indistinguishable from each other and the placebo.

##### *3.8.1.3. Concurrent MS therapies*

Participants will be permitted to continue, start or discontinue any currently approved SOC therapy for their form of MS.

##### *3.8.1.4. Dose escalation*

Both active study interventions will be commenced at half dose (spironolactone 25 mg twice daily and famciclovir 250 mg twice daily) for the first 4 weeks. Subject to satisfactory review of adverse events and laboratory investigations, dosage will be increased to the full dose at 4 weeks. In the event of concern regarding adverse events or changes in laboratory parameters there will be options to either, (1) discontinue the study intervention or (2) continue with the half-dose. If all parameters are satisfactory then the dose will be escalated to the full dose for both active treatment arms. Bottles of placebo will be marked as either half-dose or full dose, but will contain identical capsules.

##### *3.8.1.5. Dose reduction*

If during the follow up phase of both Stage 1 and Stage 2 of the STOP-MS trial the site PI suspects that a participant may be experiencing clinical or laboratory parameter IMP-related adverse event (AE) then they can elect to:

- Cease the IMP
- Reduce the dose of the IMP to half-dose for the remainder of the study.

In the case of IMP cessation, participants will be encouraged to remain in the study and complete all remaining visits and assessments as per the protocol so that their complete data can be used in the intention-to-treat analysis.

#### ***3.8.2. Study Drug Accountability***

A nominated pharmacist at each participating site will maintain an inventory of receipt, use, return and collection of study drug.

##### *3.8.2.1. Control of Supplies*

The nominated site pharmacist at each site is responsible for recording the dispensing of the product to participants on an investigational product dispensing log which will be recorded in

the REDCap® database. Accurate records will be maintained demonstrating dates and amount of product received, to whom dispensed, and accounts of any product accidentally or deliberately destroyed. Clinical trial materials will not be loaned or dispensed to another investigator or trial centre or used for any purpose other than the trial without the prior approval of the trial coordinator and site PI.

##### *3.8.2.2. Return of Supplies*

At the conclusion of the trial, the Principal Investigator (or delegate) will perform a final inventory. If any supplies cannot be accounted for, this will be documented on the product accountability form together with an explanation of the discrepancy. The original version of the product accountability and dispensing logs must be sent to the primary trial coordinator. The Principal Investigator will retain copies of these logs on file.

##### *3.8.2.3. Retention of Samples*

The Principal Investigator (or delegate) will maintain accurate records demonstrating dates and amount of product received, to whom dispensed, and accounts of any product accidentally or deliberately destroyed. It will be the responsibility of the Principal Investigator or delegate to ensure that adequate samples of all trial doses are retained in accordance with the relevant regulatory guidelines. For example, for trials conducted under the CTN scheme in Australia, at least a sample from each batch of product used in the trial should be retained for one year longer than the shelf-life of the product, and to comply with International Conference on Harmonisation – Good Clinical Practice (ICH-GCP) requirements, sufficient quantities of the product(s) should be retained either until the analyses of the trial data are complete or as required by the applicable regulatory requirements, whichever represents the longer retention period.

#### ***3.8.3. Study Procedures and Visits***

Participants will be required to undergo the following study procedures and visits.

***Screening Visit (-28 to -7 days)***

- Written informed consent will be provided
- Comprehensive review of medical history including; details of MS history, other past medical and surgical history, family history, social history and allergies
- Alcohol consumption will be assessed using Standard Drinks (see Appendix 28)
- Review of past and current medications and health supplements
- Comprehensive medical and neurological examination
- EDSS[27]
- MSReactor computerised cognitive assessment (optional)[30]
- Hospital Anxiety and Depression Scale (HADS)[60]
- Blood tests – full blood count (FBC), electrolytes, urea creatinine (EUC), eGFR, liver function tests (LFT)
- EBNA1 titres
- Pregnancy test (for women of child-bearing age)
- Collection and storage of DNA sample (biobanking)

- Collection and storage of serum sample (biobanking)
- Saliva collection for EBV DNA polymerase chain reaction (PCR) quantification
- MRI Brain (as routine standard of care – non-study protocol scan – see below for time constraints for MRI)

***Enrolment Visit (Day 0)***

- Review of neurological symptoms/relapse
- Review of concomitant medications and supplements
- EDSS[27]
- MS Functional Composite (MSFC)[28] - including T25FW, 9-HPT and SDMT
- MSReactor computerised cognitive assessment (optional)[30]PROMs – all PROMs will be administered online through REDCap®
    - Multiple Sclerosis Impact Scale-29 (MSIS-29)[61]
    - Multiple Sclerosis Walking Scale-12 (MSWS-12)[62]
    - Neuropathic Pain Scale (NPS)[63]
    - Fatigue Scale Motor and Cognitive Functions (FSMCF)[64]
    - EuroQol – 5 Domains – 5 Levels (EQ-5D-5L)[65]
- Completion of eligibility criteria checklist
- Randomisation
- Participants will be provided with a Participant ID Card (see Appendix 23)
- Participants will be provided with a list of drugs and supplements that can cause hyperkalaemia that they should avoid taking (see Appendix 22)

***Safety Blood Tests (+/- 7 days)***

- EUC, eGFR – weeks 1, 3, 6 and 12 weeks and then every 12 weeks
- FBC, LFTs – every 24 weeks

***Outcome Serum Collection (+/- 7 days)***

- EBNA1 titres – weeks -4 and week 24 (Stage 1 only)

***Biobanking Serum Collection (+/- 7 days)***

- Collection and storage of serum sample for biobanking (optional) – weeks -4, 24 (Stage 1) only, then weeks 48, 96 and 144
- Collection and storage of DNA sample for biobanking (optional) – week -4

***Saliva Collection (+/- 7 days)***

- EBV DNA detection – weeks -4, 0, 4, 8, 12, 16, 20 and 24 (Stage 1 only)

***Dose Escalation Visit (week 4 +/- 7 days)***

- Review of adverse events
- Review of concomitant medications and supplements
- MSReactor computerised cognitive assessment (optional)[30]
- Review of FBC, LFTs, EUC and eGFR
- Decision regarding dose escalation

***Review Visits (Every 24 weeks; +/- 7 days in first 6 months then +/- 14 days)***

- Review of relapse history
- Review of adverse events
- Review of intercurrent illness
- Review of concomitant medications and supplements
- Review of current MS SOC therapy
- General physical examination
- EDSS[27]
- MSFC[28]
- MSReactor computerised cognitive assessment (optional)[30]
- Compliance monitoring (remaining pill count)

General examination will include specific examination for gynaecomastia in males.

Review visits may be conducted remotely via telehealth whether for reasons of COVID-lockdowns or remoteness of the participant.

The following PROMs will be repeated at annual review visits (weeks 48, 96, 144 and 192):

- MSIS-29[61]
- MSWS-12[62]
- Neuropathic Pain Scale[63]
- FSMCF[64]
- EQ-5D-5L[65]

***Telephone Monitoring (Every 24 weeks; +/- 7 days in first 6 months then +/- 14 days – offset from Review Visits by 12 weeks)***

- Review of relapse history
- Review of adverse events
- Review of intercurrent illness
- Review of concomitant medications
- MSReactor computerised cognitive assessment (optional)[30]

***MRI (At baseline and every 48 weeks - +/- 90 days)***

- MRI Brain (as part of SOC – non-protocol scans)

The full schedule of study events is provided in Appendix 25.

The timeline of participant involvement and total time commitment per patient in the trial is outlined in Table 2.

**Table 2. Summary of patient participation as total time in hours***

| Procedure | Screen | MRI Brain 1 | Enrolment | Dose Escaltion Visit | 24 Week Visit | MRI Brain 2 | 48 Week Visit | 72 Week Visit | MRI Brain 3 | 96 Week Visit | 120 Week Visit | MRI Brain 4 | 144 Week Visit | 168 Week Visit | MRI Brain 5 | 192 Week Visit | Time (Hrs) | Total Time (Hrs) |
|---|---|---|---|---|---|---|---|---|---|---|---|---|---|---|---|---|---|---|
| Time (wks) | -1 | -1 | 0 | 4 | 24 | 48 | 48 | 72 | 96 | 96 | 120 | 144 | 144 | 168 | 192 | 192 | | |
| Screening Visit | 1 | | | | | | | | | | | | | | | | 1 | 1 |
| Clinical Rev | | | 1 | | 1 | | 1 | 1 | | 1 | | | 1 | 1 | | 1 | 0.5 | 4 |
| EDSS | 1 | | 1 | | 1 | | 1 | 1 | | 1 | | | 1 | 1 | | 1 | 0.5 | 4.5 |
| MRI | | 1 | | | | 1 | | | 1 | | | 1 | | | 1 | | 1 | 5 |
| Bloods | 1 | | | 1 | 1 | | 1 | 1 | | 1 | 1 | | 1 | 1 | | 1 | 0.25 | 2.5 |
| Saliva | 1 | | | 1 | 5 | | | | | | | | | | | | 0.1 | 0.7 |
| | | | | | | | | | | | | | | | | | Total participant hours | 17.7 |

*Estimate of participant time commitment does not include telephone reviews

Follow up will be for a minimum of 2 years and a maximum of 5 years, with a mean of approximately 3 years. This assumes that the trial progresses to the completion of Stage 2.

### 3.9. Outcome Measures

#### *3.9.1. Stage 1*

The co-primary outcome measures for stage 1 will be:

- Salivary EBV DNA detection
- Serum EBNA1 antibody titres

During stage 1, saliva samples (1-2 ml) will be collected monthly by participants at home which they will then post to QIMR-Berghofer using a pre-paid envelope and kit designed specifically for this purpose. DNA will be extracted using a saliva DNA extraction kit (Qiagen®, Netherlands). EBV DNA will be detected using a TaqMan® assay and EBV shedding considered present if viral count is >5.8 virus copies/µl. A serum sample will be collected at week -4 and at week 24 and separated using a centrifuge and pipette. Serum will be stored locally at -70 °C and shipped to QIMR-Berghofer in batches every 6 months. Enzyme-linked immunosorbent assay kits (Diamedix®, FL, US) with serial dilutions will be used to measure EBNA1 antibody titres with normalisation to the manufacturer's cut-off calibrator standard. These tests will be performed in the laboratory of Chief Investigator (CI)

Smith (QIMR-Berghofer, QLD). Standard Operating Procedures (SOPs) will be provided for these processes and should be followed by all participating sites.

### *3.9.2. Stage 2*

The Stage 2 primary outcome measure will be time to 6mCDP using a composite of EDSS,[27] T25FW[28] and 9-HPT.[29] over a period of between 144 and 240 weeks (variation depending on if participants have been retained in same treatment arms in Stage 1 and Stage 2). Definitions of progression will be: an increase in EDSS (of 1 point if baseline EDSS was <5.5, or 0.5 points if baseline EDSS was ≥5.5); ≥20% increase in 9-HPT time; or ≥20% increase in T25FW. Any qualifying change must be confirmed at repeat assessment 6 months later. Inclusion of a measure of upper limb function (9-HPT) also addresses consumer interest in assessing arm function and is particularly important for people with higher levels of disability. This composite definition of 6mCDP has been chosen for other recent clinical trials in MS.[66]

### *3.9.3. Secondary Outcome Measures*

The following outcome measures will also be used.

#### *3.9.3.1. Clinical*

Time to first relapse, time to 6mCDP based on EDSS alone and mean changes in EDSS, MS Functional Composite (MSFC) Score,[28] utilising the Symbol Digit Modalities Test (SDMT)[67] in place of the Paced Auditory Serial Addition Test, T25FW and 9-HPT.[68]

Cognitive assessment using MSReactor computerised assessment will also be collected. Participants will be requested to complete the MSReactor tasks at the first three trial visits and then once every 3 months. The tasks can be completed within 5 minutes. The recorded reaction times will be uploaded to a server hosted by Monash University in a de-identified format, using the participants MSReactor login details (email and user-defined password). These data will be compared with traditional measures of physical disability (EDSS) and cognitive function (SDMT) to assess if the MSReactor reaction times are any more sensitive in detecting change over time as a purely exploratory outcome measure. Participants may choose to opt-out of this part of the study.

#### *3.9.3.2. MRI*

MRI brain will be performed annually as part of SOC. We will leverage existing standard protocols for routine clinical MRI brain which have been implemented at the participating sites as part of the existing MSBase collaboration. These scans include fixed acquisition protocols that include 3D volumetric T1 (with and without Gd) and FLAIR sequences. New and enlarging lesion counts between timepoints, and Gd-enhancing lesion counts will be documented as reported by the local radiologist/neurologist. Subject to additional study funding, we will have the potential to use an artificial intelligence-based software with additional manual quality assurance by trained neuroimaging analysts to detect new/enlarging lesions and measure whole brain atrophy (SIENA technique)[69] through the Sydney Neuroimaging Analysis Centre (SNAC) under CI Barnett.

#### 3.9.3.3. PROMs

The MS Impact Scale-29,[61] which measures physical and psychological wellbeing, the MS Walking Scale-12,[62] Neuropathic Pain Scale[63] and the Fatigue Scale for Motor and Cognitive Functions[64] will be measured every 12 months. At each visit the number and severity of relapses since the last study visit will be recorded.

Health economics: A treatment that slows disability progression could represent a highly cost-effective use of public health resources given the high costs of progressive MS and the very low cost of repurposed drugs. Cost effectiveness from a health payer and societal perspective will be measured by cost per quality-adjusted life year (QALY) and will be assessed 6-monthly using the EQ-5D-5L.43 For the analysis, results will be reported as the incremental cost per QALY gained as well as the expected net-benefit statistic. We will use societal cost data from our previously published work to attribute avoided societal costs with reduced disability progression.[65]

PROMs surveys will be administered online directly into REDCap by participants. This will require participants to provide an email address and a first name (so that emails can be personalised). This potentially identifiable will be held in the REDCap database in such a way that this is only visible to staff at the site where the participant enrolled and will be marked as ‘identifiable’ in REDCap which means that this data cannot be seen or exported by any of the researchers on the project.

#### 3.9.3.4. Adverse Events

All AEs will be recorded in the eCRF. Information recorded will include the date of onset of the AE, a description of the AE, severity, relationship to IMP, whether or not the AE is an SAE and date of resolution or if ongoing. MS relapses will also require additional information to be reported in the on-study relapse form. All SAEs must be reported to the Sponsor and the lead HREC within 24 hours of study staff being made aware of the event. All adverse events will be coded according to the Common Terminology Criteria for Adverse Events v5.0 (CTCAE), National Cancer Institute, National Institutes of Health, MD, US. Severity will also be graded according to CTCAE v5.0 definitions. The CTCAE definitions for blood result AE grades will also be used (see Appendix 29).

SAE will be defined as any AE that:

- Results in death
- Is life-threatening – reported medical condition has a known substantial risk of death
- Results in hospitalisation – visits to the emergency department will not be counted as SAE unless other criteria apply
- Results in disability or permanent damage – a substantial change to a person’s ability to conduct their usual functions (i.e. significant persistent or permanent change, impairment, damage or disruption to body function/structure, physical activities or quality of life)
- Results in a congenital anomaly or birth defect
- Required significant intervention to avoid injury (e.g. treatment for an allergic reaction or seizures in an emergency department)
- Results in any other serious ongoing change in health (e.g. drug dependence, blood dyscrasias)

De-identified, aggregate AE data will be reviewed by the Data Safety Monitoring Board (DSMB) every 3 months. Once a minimum of 50 AEs have been reported these data will be summarised according to treatment allocation (unblinded) and analysed statistically using Fisher's exact test for comparison between each of the active treatment arms and placebo. Comparisons will be made for all treatment emergent AEs, treatment related AEs (definite or probable), higher grade AEs (grade 2 or 3), SAEs and deaths. SAEs as they occur, will be reported to the sponsor and the lead HREC within 24 hours of site study staff being made aware of them and to local HREC/RGO in accordance with local guidelines. Comparisons will also be made individual AE conditions where more than 5 events have occurred. Event rates will be compared with reported expected rates of these AEs for the two active treatments using 95% confidence intervals. Any rates significantly outside of these expected limits will be reported to the Sponsor and lead HREC. A data safety report will be provided to the Sponsor and lead HREC annually.

##### *3.9.3.5. Data Collection*

Data will be collected in an eCRF created using a specifically created REDCap® database. This database will be held on servers maintained by Griffith University and access to the database online will be restricted to study personal at the participating sites and necessary regulatory authorities. Access to the data will be further restricted according to utilisation requirements and for maintaining blinding.

The data to be collected is indicated in the Data Collection Sheet (see Appendix 26) and PROMs (Appendix 27).

In addition, data from the MSReactor mobile phone app will also be collected. Participants will be requested to complete the MSReactor tasks on their own mobile phone once every 3 months. The tasks can be completed within a minute or two. The recorded reaction times will be uploaded to a server hosted by Monash University in a de-identified format, using the participants PIN. These data will be compared with traditional measures of physical disability (EDSS) and cognitive function (SDMT) to assess if the MS Reactor reaction times are any more sensitive in detecting change over time as a purely exploratory outcome measure. This component of the study is optional.

#### ***3.9.4. Data Storage and Confidentiality***

Participants' privacy and confidentiality will be protected through the following:

- The PICF will be held in dedicated files for each participant at individual sites. These files will be held in a secure location (locked room, which only clinical staff will have access to).
- Patient specific data will be stored within medical case note files within medical records departments or in electronic medical record systems at each site. Medical records departments at state health facilities have restricted access that only permits those with requisite authority to have access to the files. Integrated electronic medical record system are password protected.
- This is a clinical trial and for safety reasons (accurate identification of participants) it will be essential that all clinically related records are identifiable. However, all

centrally recorded patient information (e.g. eCRF) and correspondence with regulatory bodies will be de-identified. All such records will use a unique participant identification code. Data connecting participant identifying information (participant folders and medical records) will also carry this code ensuring that all participants are potentially re-identifiable at the individual site. These identifiers will not be removed at each site at any point in case of the urgent need to re-identify a participant for safety reasons (e.g. unblinding of treatment allocation). All safety and statistical analyses will be conducted on fully de-identified data.

- In accordance with international publication and regulatory requirements fully de-identified aggregate data and where necessary raw data will be made available for independent verification of results and regulatory processes. These data will be held on servers at Griffith University in password protected files and only released to third parties if required for regulatory purposes or if requested from other researchers after approval by local HREC.
- Under medical practice guidelines and laws participants will have access to their own clinical data as held in the medical records at each participating site.
- All records will be kept for a minimum of 15 years. Paper and electronic documents will be held at each site as part of existing medical record protocols. Trial related documents (participant folders) will be stored locally using secure storage facilities. All central electronic data will be stored on Griffith University servers in password protected files.
- In accordance with recommendations of the funding body (MRFF) after the full analysis of data has been completed, fully de-identified data from the trial will be posted to an open-access data repository such as Figshare.
- Specific consent for potential future use of bio-banked samples (serum and DNA samples) together with de-identified clinical data has been included in PICF. The precise nature of these studies will depend upon the outcomes of the trial, but the current proposals and general outline of possibilities is included below.

### ***3.9.5. Data Analysis and Statistical Considerations***

Further details regarding data analysis can be found in the Statistical Analysis Plan.

#### *3.9.5.1. Stage 1*

Primary endpoints:

Proportion of patients with the composite primary end point of:

- EBV DNA present in saliva at 4 time points - in weeks 12, 16, 20 or 24,

OR

- increase/no change in EBNA1 IgG titre in serum at month 6 post-baseline compared to the study baseline.

**Arm termination rule:**

A treatment arm will be terminated at the end of Stage 1 if the absolute difference in the proportions of patients meeting the composite primary endpoint does not favour the intervention over placebo by at least 10% of the participants within the arm with a 95% confidence.

In the event of both drugs being tested proving to be highly effective in reducing evidence of EBV activity in Stage 1, the CI team will seek additional funding (e.g. MS Australia, National MS Society (US)) to support a third arm in Stage 2. If spironolactone (the cheaper of the two agents) proves to be the more effective agent in Stage 1, then the CI team would seek approval from the Medical Research Future Fund (MRFF) to divert the associated cost savings towards proposed biomarker studies.

If both agents fail to reach nominal levels of significance in Stage 1, in the case of borderline results or presence of a trend in support of effectiveness against EBV then the CIs would seek approval from MRFF to potentially extend participant numbers in Stage 1. Otherwise, the trial would be abandoned.

##### *3.9.5.2. Stage 2*

Primary Endpoint:
The ratio of cumulative hazards of 6mCDP using a composite of EDSS, T25FW and 9-HPT over a period of 240 weeks.

Secondary endpoints:

(Study periods as defined under Primary endpoints, Stage 2):

- The ratio of cumulative hazards of experiencing first relapse.
- The ratio of cumulative hazards of 6mCDP using EDSS only.
- Mean change in EDSS and MSFC scores.
- Cumulative number of new or enlarging lesion during the study period.
- Patient reported outcomes: Mean change in MSIS-29, MSWS-12, Neuropathic Pain Scale, FSMCF, recorded every 48 weeks during the study period
- Economic Impact: Change in EQ-5D-5L over time using both the overall impact score (visual analogue scale) and the 5-digit health state data. Economic impact will be assessed using utility values derived from Australia specific value sets.

Hypotheses will be tested at $\alpha \le 0.05$. In accordance with the US Food and Drug Administration guidance on platform trials, Type I error will not be controlled across treatment arms. The comparisons are pairwise against the control study arm. Appropriate point (mean) and interval estimates (95% confidence interval) will be calculated for the associations of interest for the primary, secondary and exploratory analyses.

Treatment arm allocations will be modelled as fixed terms. In mixed-effect models, multiple entries will be included for repeated measures of the outcomes over time, with patient ID included as random intercept.

#### *3.9.6. Sample size and power calculations*

**Stage 1**

EBV DNA is expected to be detected in 48% of the participants in the placebo arm. Based on a placebo-controlled RCT of teriflunomide, the proportion of patients in whom EBNA1 IgG titre will decrease is expected to be 35-55%. Thus, the number of participants reaching the

composite endpoint at the end of Stage 1 is predicted to range from 50% to 70%. An absolute reduction in this rate of 10% would be considered as clinically meaningful.

**Stage 2**

Sample size calculations are based on α=0.05. 6mCDP is expected to be detected in 40% of the participants in the placebo arm. Based on the placebo-controlled ORATORIO trial, a sample size of 132 participants in each arm will provide 80% power to detect a 40% relative reduction in 6mCDP to 24% of participants in the active treatment arms. Accounting for a 14% drop-out rate, 150 participants will be required in each arm.

We aim to recruit 50 participants into each of the 3 arms in Stage 1. After completion of the Stage 1 and re-randomisation of one of the treatment arms, we will recruit and additional 100 participants into each of the arms regained in Stage 2.

### 3.10. Biobanking and Future Studies

A part of Stage 1 the 4-weekly collection of saliva for the detection of EBV DNA from baseline to week 24 and the collection of serum samples at baseline and 24 weeks for EBNA1 antibody titres has been planned as part of primary outcome measures of the STOP-MS trial. In addition, it is proposed that blood for DNA extraction be collected at baseline and blood for serum separation be collected at baseline and every 48 weeks until the end of the trial. At present the research team have not secured funding to support this component of the project, but further applications for funding are planned. Specific consent for collection and storage of these samples will be included on the consent form ("opt-in" check box).

Specific studies to be potentially included are:

1. Whole blood (EDTA tube) will be stored locally at -70 °C and then transferred to a central laboratory (site yet to be determined, but in Australia) for DNA extraction. Genomwide sequencing for allelic variants of single nucleotide polymorphisms would be performed using chip technology on a standard platform (Affymetrix or Illumina). These data would be used to explore potential genetic markers of a positive clinical response to a particular treatment or correlation with clinical or laboratory markers of disease outcomes (e.g. disability or biomarker levels).
2. Whole blood samples (SST tube) will be separated using a centrifuge at each participating site and serum samples pipetted and aliquoted into 2 x 4 ml tubes and sored locally at -70 °C. These samples will be transferred to central facilities within Australia (sites yet to be determined) for further analyses as outlined below at regular intervals (e.g. annually).
3. Serum samples will be tested for EBNA1 antibody titres (most likely at QIMR/Berghofer Institute, Queensland) and potentially for other antibodies (e.g. VCA IgG).
4. Serum samples will be tested for serum neurofilament light levels as a marker of CNS axonal degeneration using SIMOA technology at one of several potential sites that can provide this service. Other potential markers of CNS degeneration may also be measured (e.g. GFAP, NCAM1).
5. Serum samples might also be used for other exploratory studies of immune cytokines and other soluble markers.

The research team have long established collaborations with the International MS Genetics Consortium who have been successful in identifying over 200 genetic loci associated with

MS susceptibility. Current endeavours are focused on potential genetic markers of progressive MS. Since the STOP-MS will be specifically recruiting people with progressive MS we would propose that fully de-identified genomic data (from analyses conducted in Australia on STOP-MS participants who have consent to such use) be combined in meta-analyses with other international cohorts.

Any additional future studies (outside of the scope of those outlined above) using biobanked samples from STOP-MS would require approval from the lead HREC.

Any approach to use de-identified data from STOP-MS by any third party would require a written proposal and approval of the lead HREC.

## 4. Translation to Changes in Clinical Practice

If spironolactone or famciclovir prove to be effective in reducing disability progression in people with MS, the CI team will apply to the Therapeutic Goods Administration for a new indication (progressive MS) for the relevant drug and prepare new treatment guidelines. The results of this study will be reported in a leading neurology journal. This study will be registered with the Australian and New Zealand Clinical Trials Registry and the results will be reported there when finalised. At the completion of the study, a lay summary of the results will be provided to participants (where they have requested this on the PICF) and will also be posted on the Griffith University Facebook page and the MS Australia website.

## 5. Timeline

It is proposed that this clinical trial will be conducted over a 5-year time span. All necessary regulatory approvals, HREC approval and funding will be sought in July 2023, with a proposed start date for the trial in January 2025. Recruitment of participants will span the first 3 years and follow up will continue for a minimum of 96 week (maximum of 240 weeks). Please see Gant chart of proposed timeline in Table 3.

**Table 3. Gantt chart of proposed timeline.**

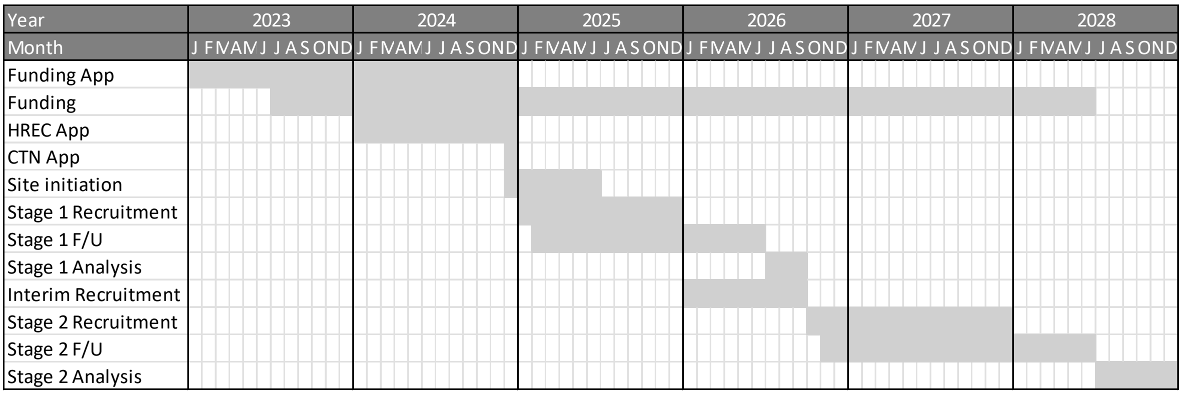

# 6. Funding and Resources

This project is being funded by an MRFF grant of $1,999,362.50 which was awarded in May 2023. Griffith University will be the sponsor of the trial.

These funds will be utilised to cover the costs of a clinical trial manager to be based at Griffith University a clinical trial monitor, manufacture of the investigational medical product, all site related costs related to conduct of the study and all of the proposed monitoring and investigational laboratory tests.

Additional funding for the proposed analysis of biobanked sample will be sought.

## 6.1. Governance

### *6.1.1. Independent Steering Committee*

The STOP-MS clinical trial will be overseen at a strategic level by the Australian MS Clinical Trials Platform Steering Committee which has been established for the PLATYPUS clinical trial funded by MS Australia. This committee consisting of national and international leaders in neurology, immunology, clinical trial design and statistics will convene twice per year to review trial design and their progress at a strategic level. The members of the committee will have no direct involvement in the conduct of any of the trials that they oversee. The Coordinating Chief Investigators of each trial (PLATYPUS, STOP-MS and a third study which also received MRFF funding, known as Fatigue In Relapsing Multiple Sclerosis – Epstein Barr Virus (FIRMS-EBV)) will report to the committee and seek guidance on specific issues and overall direction of the projects. This will include oversight of trial budgets. Members of the committee will be paid an honorarium for their services. Professor Pam McCombe (MS Neurologist at Royal Brisbane and Women’s Hospital) has kindly agreed to chair this committee.

The Steering Committee and investigators have agreed to an overarching Charter that will used to direct the conduct of activities of the Australian MS Clinical Trials Platform (see Appendix 30).

### *6.1.2. Investigator Meetings*

The Coordinating Chief Investigator will convene investigator meetings, initially once per fortnight in the setup phase and then monthly thereafter. All site PI’s, AI’s, clinical trial coordinators and pharmacists will be invited to the investigatory meetings. The purpose of these meetings will be to finalise the protocol, ensure consistent practices at all sites, relay any amendments, monitor progress of the trial and troubleshoot any day-to-day matters.

### *6.1.3. Data Safety Monitoring Board*

The data safety monitoring board will convene every 3 months to review summary reports of adverse events every 3 months. These meetings will commence once any adverse events have occurred. When fewer than 50 participants have been recruited reports will remain blinded. Once 50 participants have been recruited data safety reports will be presented in di-identified, aggregate, unblinded format to permit potential trends in safety profile between the investigational medicinal products. In addition to being reported to the relevant HREC

within 24 hours of notification, any SAE's will be reported to the Data Safety Monitoring Board out of session for comment and if there are significant concerns an ad hoc meeting will be convened. The members of the Data Safety Monitoring Board will be independent of the STOP-MS trial and will be shared with the PLATYPUS and FIRMS-MS trials. The members will be paid an honorarium for their time.

#### *6.1.4. Sponsor*

Griffith University will be the sponsor of the STOP-MS clinical trial. Griffith University is a leading public university in Australia with over 49,000 students, 4,000 academic staff and over $100 million in research funding for 2023. Griffith University holds indemnity insurance covering claims up to $25 million for research activity including clinical trials. The clinical trials unit at Griffith University has the staff and physical resources to provide clinical trial management and monitoring services.

#### *6.1.5. Trial Site Resources*

The STOP-MS Clinical Trial sites will be Academic MS Centres located in major public hospitals, private hospitals and universities. The site PIs are all experience MS Neurologists who have considerable experience of both investigator-driven and commercially sponsored clinical trials.

All key site staff (PIs, AIs, clinical trial coordinators and pharmacists) will be required to have current ICH-GCP certification and provide a current CV. Site PIs and AIs will be required to have current EDSS certification (performed as per Neurostatus® guidelines – neurostatus.net). Clinical trial coordinators/nurses will be required to have completed training for the 9-HPT and SDMT. Note that EDSS assessments need to be conducted by an independent rater (who has no role in routine care of the participant or other study related procedures, please see STOP-MS-SOP-012 for further details.

Trial sites will be required to have the following before trial commencement:

- A nominated PI
- A nominated AI (independent EDSS rater)
- A nominated clinical trial coordinator/nurse
- A nominated pharmacist
- A suitable space for clinical visits (e.g. clinical trials unit, clinic space)
- A paper-based or electronic medical record system with appropriate storage in case of paper-based system
- Secure storage space for relevant study files
- An accredited pharmacy
- An ECG machine and staff trained in performing ECGs
- Access to a centrifuge capable of 2000g (preferably refrigerated)
- Access to a -70 °C freezer
- EDTA and Serum collection tubes
- Measured 25-foot walking area
- Area suitable for assessing walking distances of up to 500m
- 9-HPT kit
- 6 m Snellen chart

The Sponsor will provide to sites the following:

- Templates for data collection
- 9-HPT kit (if required)
- Symbol Digit Modalities Test kit and score sheets
- Labels for biobanking samples

Sites will be responsible for facilitating relevant local HREC and SSA approvals.

Engagement of clinical sites will be through a Clinical Trial Research Agreement between the Sponsor and the host institution.

# 7. References


1. Weinshenker BG, Ebers GC. The natural history of multiple sclerosis. Can J Neurol Sci 1987;14:255-261.

2. Confavreux C, Vukusic S. The clinical course of multiple sclerosis. Handb Clin Neurol 2014;122:343-369.

3. Ahmad H, Palmer AJ, Campbell JA, van der Mei I, Taylor BV. Health Economic Impact of Multiple Sclerosis in Australia in 2017. Sydney, Australia: Menzies Institute for Medical Research, University of Tasmania, 2018 Aug 2018.

4. Brown JWL, Coles A, Horakova D, et al. Association of Initial Disease-Modifying Therapy With Later Conversion to Secondary Progressive Multiple Sclerosis. Journal of the American Medical Association 2019;321:175-187.

5. Handel AE, Williamson AJ, Disanto G, Handunnetthi L, Giovannoni G, Ramagopalan SV. An updated meta-analysis of risk of multiple sclerosis following infectious mononucleosis. PLoS One 2010;5:e12496.

6. Hernan MA, Zhang SM, Lipworth L, Olek MJ, Ascherio A. Multiple sclerosis and age at infection with common viruses. Epidemiology 2001;12:301-306.

7. Endriz J, Ho PP, Steinman L. Time correlation between mononucleosis and initial symptoms of MS. Neurol Neuroimmunol Neuroinflamm 2017;4:e308.

8. Jacobs BM, Giovannoni G, Cuzick J, Dobson R. Systematic review and meta-analysis of the association between Epstein-Barr virus, multiple sclerosis and other risk factors. Mult Scler 2020;26:1281-1297.

9. Simon KC, O'Reilly EJ, Munger KL, Finerty S, Morgan AJ, Ascherio A. Epstein-Barr virus neutralizing antibody levels and risk of multiple sclerosis. Mult Scler 2012;18:1185-1187.

10. Bjornevik K, Cortese M, Healy BC, et al. Longitudinal analysis reveals high prevalence of Epstein-Barr virus associated with multiple sclerosis. Science 2022;375:296-301.

11. Gold J, Holden D, Parratt J, et al. Effect of teriflunomide on Epstein-Barr virus shedding in relapsing-remitting multiple sclerosis patients: Outcomes from a real-world pilot cohort study. Multiple sclerosis and related disorders 2022;68:104377.

12. Zivadinov R, Ramanathan M, Hagemeier J, et al. Teriflunomide's effect on humoral response to Epstein-Barr virus and development of cortical gray matter pathology in multiple sclerosis. Multiple sclerosis and related disorders 2019;36:101388.

13. James ND, Clarke NW, Cook A, et al. Abiraterone acetate plus prednisolone for metastatic patients starting hormone therapy: 5-year follow-up results from the STAMPEDE randomised trial (NCT00268476). Int J Cancer 2022;151:422-434.

14. Group RC, Horby P, Lim WS, et al. Dexamethasone in Hospitalized Patients with Covid-19. N Engl J Med 2021;384:693-704.

15. Serafini B, Rosicarelli B, Franciotta D, et al. Dysregulated Epstein-Barr virus infection in the multiple sclerosis brain. J Exp Med 2007;204:2899-2912.

16. Magliozzi R, Serafini B, Rosicarelli B, et al. B-cell enrichment and Epstein-Barr virus infection in inflammatory cortical lesions in secondary progressive multiple sclerosis. J Neuropathol Exp Neurol 2013;72:29-41.

17. Lanz TV, Brewer RC, Ho PP, et al. Clonally expanded B cells in multiple sclerosis bind EBV EBNA1 and GlialCAM. Nature 2022;603:321-327.

18. Lunemann JD, Jelcic I, Roberts S, et al. EBNA1-specific T cells from patients with multiple sclerosis cross react with myelin antigens and co-produce IFN-gamma and IL-2. J Exp Med 2008;205:1763-1773.

19. Kreft KL, Van Nierop GP, Scherbeijn SMJ, Janssen M, Verjans G, Hintzen RQ. Elevated EBNA-1 IgG in MS is associated with genetic MS risk variants. Neurol Neuroimmunol Neuroinflamm 2017;4:e406.

20. Afrasiabi A, Parnell GP, Fewings N, et al. Evidence from genome wide association studies implicates reduced control of Epstein-Barr virus infection in multiple sclerosis susceptibility. Genome Med 2019;11:26.

21. Harley JB, Chen X, Pujato M, et al. Transcription factors operate across disease loci, with EBNA2 implicated in autoimmunity. Nat Genet 2018;50:699-707.

22. Pagano JS, Whitehurst CB, Andrei G. Antiviral Drugs for EBV. Cancers (Basel) 2018;10:197.

23. Zdimerova H, Murer A, Engelmann C, et al. Attenuated immune control of Epstein-Barr virus in humanized mice is associated with the multiple sclerosis risk factor HLA-DR15. Eur J Immunol 2021;51:64-75.

24. Pender MP, Csurhes PA, Pfluger CM, Burrows SR. Deficiency of CD8+ effector memory T cells is an early and persistent feature of multiple sclerosis. Mult Scler 2014;20:1825-1832.

25. AbuSalah MAH, Gan SH, Al-Hatamleh MAI, Irekeola AA, Shueb RH, Yean Yean C. Recent advances in diagnostic approaches for Epstein-Barr virus. Pathogens 2020;9:597.

26. Pardini M, Cutter G, Sormani MP. Clinical trial design for progressive MS trials. Mult Scler 2017;23:1642-1648.

27. Kurtzke JF. Rating neurologic impairment in multiple sclerosis: an expanded disability status scale (EDSS). Neurology 1983;33:1444-1452.

28. Cutter GR, Baier ML, Rudick RA, et al. Development of a multiple sclerosis functional composite as a clinical trial outcome measure. Brain 1999;122 ( Pt 5):871-882.

29. Goodkin DE, Hertsgaard D, Seminary J. Upper extremity function in multiple sclerosis: improving assessment sensitivity with box-and-block and nine-hole peg tests. Arch Phys Med Rehabil 1988;69:850-854.

30. Merlo D, Darby D, Kalincik T, Butzkueven H, van der Walt A. The feasibility, reliability and concurrent validity of the MSReactor computerized cognitive screening tool in multiple sclerosis. Ther Adv Neurol Disord 2019;12:1756286419859183.

31. Samanani S, Mishra M, Silva C, et al. Screening for inhibitors of microglia to reduce neuroinflammation. CNS Neurol Disord Drug Targets 2013;12:741-749.

32. Verma D, Thompson J, Swaminathan S. Spironolactone blocks Epstein-Barr virus production by inhibiting EBV SM protein function. Proc Natl Acad Sci U S A 2016;113:3609-3614.

33. Verma D, Church TM, Swaminathan S. Epstein-Barr virus co-opts TFIIH component XPB to specifically activate essential viral lytic promoters. Proc Natl Acad Sci U S A 2020;117:13044-13055.

34. DeRenzi A, Penico J. Lymphoproliferative disease in a non-transplant patient and spironolactone’s activity against Epstein-Barr virus. HCA Healthcare Journal of Medicine 2021;2:263-266.

35. Mertin J, Koczorek KR, Backmund H. Pilot study on the efficacy of high doses of aldosterone and spirolactonederivatives in the treatment of multiple sclerosis. Zeitschrift für Neurologie 1972;202:217-228.

36. Bacon TH, Boyd MR. Activity of penciclovir against Epstein-Barr virus. Antimicrob Agents Chemother 1995;39:1599-1602.

37. Borg N, Stahle L. Penciclovir pharmacokinetics and distribution to the brain and muscle of rats, studied by microdialysis. Antiviral Chemistry and Chemotherapy 1997;8:275-279.

38. Rafailidis PI, Mavros MN, Kapaskelis A, Falagas ME. Antiviral treatment for severe EBV infections in apparently immunocompetent patients. J Clin Virol 2010;49:151-157.

39. Goldani LZ. Treatment of severe infectious mononucleosis with famciclovir. J Infect 2002;44:92-93.

40. Bech E, Lycke J, Gadeberg P, et al. A randomized, double-blind, placebo-controlled MRI study of anti-herpes virus therapy in MS. Neurology 2002;58:31-36.

41. Friedman JE, Zabriskie JB, Plank C, et al. A randomized clinical trial of valacyclovir in multiple sclerosis. Mult Scler 2005;11:286-295.

42. Lycke J, Svennerholm B, Hjelmquist E, et al. Acyclovir treatment of relapsing-remitting multiple sclerosis. A randomized, placebo-controlled, double-blind study. J Neurol 1996;243:214-224.

43. Ertl P, Snowden W, Lowe D, Miller W, Collins P, Littler E. A comparative study of the in vitro and in vivo antiviral activities of acyclovir and penciclovir. Antiviral Chemistry and Chemotherapy 1995;6:89-97.

44. Boyd MR, Safrin S, Kern ER. Penciclovir: a review of its spectrum of activity, selectivity, and cross-resistance pattern. Antivral Chem Chemother 1993;4:3-11.

45. Gardiner P, Schrode K, Quinlan D, et al. Spironolactone metabolism: steady-state serum levels of the sulfur-containing metabolites. J Clin Pharmacol 1989;29:342-347.

46. Karim A. Spironolactone: disposition, metabolism, pharmacodynamics, and bioavailability. Drug Metab Rev 1978;8:151-188.

47. Karim A, Zagarella J, Hutsell TC, Chao A, Baltes BJ. Spironolactone. II. Bioavailability. Clin Pharmacol Ther 1976;19:170-176.

48. do Campo J, Taylor V. Spironolactone as treatment for chronic fatigue syndrome in patients with positive Epstein-Barr virus serology. Int Med J2021: 15-40.

49. Alesi S, Forslund M, Melin J, et al. Efficacy and safety of anti-androgens in the management of polycystic ovary syndrome: a systematic review and meta-analysis of randomised controlled trials. EClinicalMedicine 2023;63:102162.

50. Mubareka S, Leung V, Aoki FY, Vinh DC. Famciclovir: a focus on efficacy and safety. Expert Opin Drug Saf 2010;9:643-658.

51. Tyring SK, Diaz-Mitoma F, Shafran SD, Locke LA, Sacks SL, Young CL. Oral famciclovir for the suppression of recurrent genital herpes: the combined data from two randomized controlled trials. J Cutan Med Surg 2003;7:449-454.

52. Mertz GJ, Loveless MO, Levin MJ, et al. Oral famciclovir for suppression of recurrent genital herpes simplex virus infection in women. A multicenter, double-blind, placebo-controlled trial. Collaborative Famciclovir Genital Herpes Research Group. Arch Intern Med 1997;157:343-349.

53. Cirelli R, Herne K, McCrary M, Lee P, Tyring SK. Famciclovir: review of clinical efficacy and safety. Antiviral Res 1996;29:141-151.

54. Dobson R, Holden D, Vickaryous N, et al. A phase II open-label clinical trial to determine the effect of famciclovir on Epstein-Barr virus activity as measured by EBV shedding in the saliva of patients with multiple sclerosis. In: UCL, ed. medRxiv preprint. London, UK: medRxiv preprint, 2023.

55. Thompson AJ, Banwell BL, Barkhof F, et al. Diagnosis of multiple sclerosis: 2017 revisions of the McDonald criteria. Lancet Neurol 2018;17:162-173.

56. Trinchieri A, Perletti G, Magri V, Stamatiou K, Trinchieri M, Montanari E. Drug-induced gynecomastia: A systematic review and meta-analysis of randomized clinical trials. Arch Ital Urol Androl 2021;93:489-496.

57. Engeland A, Bjorge T, Daltveit AK, et al. Effects of preconceptional paternal drug exposure on birth outcomes: cohort study of 340 000 pregnancies using Norwegian population-based databases. Br J Clin Pharmacol 2013;75:1134-1141.

58. Thomas RW, Maddox RR. The interaction of spironolactone and digoxin: a review and evaluation. Ther Drug Monit 1981;3:117-120.

59. Boutron I, Altman DG, Moher D, Schulz KF, Ravaud P, Group CN. CONSORT Statement for Randomized Trials of Nonpharmacologic Treatments: A 2017 Update and a CONSORT Extension for Nonpharmacologic Trial Abstracts. Ann Intern Med 2017;167:40-47.

60. Zigmond AS, Snaith RP. The hospital anxiety and depression scale. Acta Psychiatr Scand 1983;67:361-370.

61. Hobart J, Lamping D, Fitzpatrick R, Riazi A, Thompson A. The Multiple Sclerosis Impact Scale (MSIS-29): a new patient-based outcome measure. Brain 2001;124:962-973.

62. McGuigan C, Hutchinson M. Confirming the validity and responsiveness of the Multiple Sclerosis Walking Scale-12 (MSWS-12). Neurology 2004;62:2103-2105.

63. Rog DJ, Nurmikko TJ, Friede T, Young CA. Validation and reliability of the Neuropathic Pain Scale (NPS) in multiple sclerosis. Clin J Pain 2007;23:473-481.

64. Penner IK, Raselli C, Stocklin M, Opwis K, Kappos L, Calabrese P. The Fatigue Scale for Motor and Cognitive Functions (FSMC): validation of a new instrument to assess multiple sclerosis-related fatigue. Mult Scler 2009;15:1509-1517.

65. Campbell JA, Ahmad H, Chen G, et al. Validation of the EQ-5D-5L and psychosocial bolt-ons in a large cohort of people living with multiple sclerosis in Australia. Qual Life Res 2022:ePub.

66. Kapoor R, Ho PR, Campbell N, et al. Effect of natalizumab on disease progression in secondary progressive multiple sclerosis (ASCEND): a phase 3, randomised, double-blind, placebo-controlled trial with an open-label extension. Lancet Neurol 2018;17:405-415.

67. Benedict RH, DeLuca J, Phillips G, et al. Validity of the Symbol Digit Modalities Test as a cognition performance outcome measure for multiple sclerosis. Mult Scler 2017;23:721-733.

68. Ontaneda D, Tallantyre EC, Raza PC, et al. Determining the effectiveness of early intensive versus escalation approaches for the treatment of relapsing-remitting multiple sclerosis: The DELIVER-MS study protocol. Contemp Clin Trials 2020;95:106009.

69. Smith SM, Zhang Y, Jenkinson M, et al. Accurate, robust, and automated longitudinal and cross-sectional brain change analysis. Neuroimage 2002;17:479-489.
