# Supplementary material for: Study protocol for a multicentre, randomised, double-blinded, placebo-controlled, multi-arm, multi-stage, trial of SpironolacTone and famciclOovir in the treatment of Progressive Multiple Sclerosis to prevent disability progression: the STOP-MS trial
Source: BMJ Neurol Open. 2025 Dec 23;7(2):e001313. doi: 10.1136/bmjno-2025-001313 (PMC12730750; doi:10.1136/bmjno-2025-001313)
Supplement: online supplemental file 3 [file bmjno-7-2-s003.pdf]

List of contraindicated medications and supplements

The following is a list of medications and supplements that are contraindicated and will be exclusion criteria for enrolment into the STOP-MS trial. Participants who are enrolled will be advised that they should not commence any of these medications for the duration of the trial. If for medical or other reasons a participant is required to commence one of the medications listed below, please contact the local principal investigator to discuss whether STOP-MS study medication should be continued or not.

**Drug Class**	**Generic name**	**Trade name / *latin name***	**Comment**
Potassium Sparing Diuretic	Amiloride	Moduretic	TGA Approved
	Eplerenone	Espler; Inpler; Inspra	TGA Approved
	Spironolactone	Aldactone; Spiractin	TGA Approved
	Triamterene	Dyrenium	Not generally available
Mineralocorticoid	Canrenoate potassium	Venactone; Soldactone	Not generally available
	Drospirenone	Yaz; Yasmin	Over the counter
	Fludrocortisone	Florinef	TGA Approved
ACE Inhibitor	Benazepril	Lotensin	TGA Approved
	Captropril	Capoten	TGA Approved
	Enalapril	Acetec; Malean; Renitec; Vasotec	TGA Approved
	Lisinopril	Fibsol; Zinopril; Zestril	TGA Approved
	Moexipril	Univasc	Not generally available
	Perindopril	Coversyl; Prexum	TGA Approved
	Quinapril	Accupril; Acquin	TGA Approved
	Ramipril	Altace	TGA Approved
	Trandolapril	Dolapril; Gopten; Tranalpha	TGA Approved
Angiotensin II Receptor Blocker	Azilsartan	Edarbi	Not generally available
	Candesartan	Adesan; Candesan; Atacand	TGA Approved
	Irbesartan	Avapro; Abisart; Avsartan; Karvea	TGA Approved
	Losartan	Cozavan	TGA Approved
	Olmesartan	Olmertan; Olmetec; Olsetan	TGA Approved
	Telmisartan	Micardis; Mizart; Teltartan	TGA Approved
	Valsartan	Dilart; Diovan; Entresto; Exforge	TGA Approved
Beta-blocker	Acebutolol	Sectral	Not generally available
	Atenolol	Noten; Tensig; Tenormin	TGA Approved
	Bisoprolol	Bicard; Bispro; Bicor	TGA Approved
	Metoprolol	Metrol; Minax; Topreloc; Toprol; Mistrom; Betaloc	TGA Approved
	Nadolol	Corgard	Not generally available
	Nebivolol	Bystollic; Nebilet; Nepiten	TGA Approved
	Propranolol	Deralin; Inderal	TGA Approved

**Drug Class**	**Generic name**	**Trade name / *latin name***	**Comment**
NSAID	Celecoxib	Celaxib; Celebrex; Celexi	TGA Approved
	Diclofenac	Clonac; Fenac; Voltaren	TGA Approved
	Ibuprofen	Brufen; Advil; Bugesic; Hedafen; Herron Blue; Neurofen; Penafen	Over the counter
	Meloxicam	Melobic; Meloxibell; Movalis; Moxicam; Mobic	TGA Approved
	Naproxen	Proxen; Naprosyn	TGA Approved
Antibiotic/anti-parasitic	Trimethoprim	Alprim; Triprim; Bactrim; Resprim; Septrin	TGA Approved
	Pentamidine	Nebupent; Pentam	Not generally available
Epoetin	Epoetin alfa	Eprex	TGA Approved
	Epoetin beta	Neorecormon; Micera	TGA Approved
	Epoetin lambda	Novicrit	TGA Approved
Other	Digoxin	Sigmaxin; Lanoxin	TGA Approved
	Enoxaparin	Clexane	TGA Approved
	Lithium	Lithicarb; Quilonum	TGA Approved
	Potassium chloride	Span K; Movicol; Macrovic; Molaxole; Chlorvescent; Renastep; O. R. S.; Renastart; Kindergen	TGA Approved
	Sacubitril	Entresto	TGA Approved
Complementary Medicine/ Supplement	Alfalfa	*Medicago sativa*	Complementary medicine
	Dandelion	Blowball; Lion’s Teeth; *Taraxacum officinale*	Complementary medicine
	Horsetail	*Equestium arvense*	Complementary medicine
	Lily of the Valley	*Convallaria mjalis*; Jacob’s Ladder; Mary Bells; May Lily	Complementary medicine
	Milkweed	*Asclepias sp*	Complementary medicine
	Nettle	Stinging nettle; *Urtica dioca*	Complementary medicine
	Muscle-building supplement	Creatine; Whey; Protein	Supplement
	Salt-substitute	AlsoSalt; LoSalt	Food substitute
