# Supplementary material for: Study protocol for a multicentre, randomised, double-blinded, placebo-controlled, multi-arm, multi-stage, trial of SpironolacTone and famciclOovir in the treatment of Progressive Multiple Sclerosis to prevent disability progression: the STOP-MS trial
Source: BMJ Neurol Open. 2025 Dec 23;7(2):e001313. doi: 10.1136/bmjno-2025-001313 (PMC12730750; doi:10.1136/bmjno-2025-001313)
Supplement: online supplemental file 4 [file bmjno-7-2-s004.pdf]

STOP-MS RISK BASED MONITORING PLAN

ANZCTR NUMBER: ACTN12621001502820p

UNIVERSAL TRIAL NUMBER: U1111-1293-1787

TGA CTN SCHEME: CT-2023-CTN-03505-1-v1

TITLE: Phase III, multicentre, randomised, double-blinded, placebo-controlled, MAMS trial of SpironolacTone and famciclOvir in the treatment of Progressive MS to prevent disability progression (STOP-MS)

**WRITTEN BY**	**NAME**	**SIGNATURE**	**DATE**
Study Monitor	Lidia Madrid San Martin		*31/07/2025*
**APPROVED BY**			
Trial Manager	*Sabrina Oishi*		*31/07/2025*
**APPROVED BY**			
Coordinating Principal Investigator	*Simon Broadley*		*31/07/2025*

TABLE OF CONTENTS

1. Abbreviations ... 3

2. Introduction And Study Information ... 6

3. Monitoring Objectives ... 12

4. Roles And Responsibilities ... 12

4.1 CRA Responsibilities ... 12

4.1.2 General Oversight And Protocol Adherence ... 12

4.1.3 Data And Documentation Verification ... 13

4.1.4 Investigational Product (Imp) Accountability ... 14

5. Risk Category For The Trial ... 15

6. Monitoring Communication Plan ... 16

7. Critical Roles, Processes And Data ... 16

8. Types Of Visits And Monitoring Activities ... 18

8.1 Site Initiation Visit (SIV) (Online) ... 18

8.2 Interim Monitoring Visits (Imv) ... 21

8.3 Statistical And Remote Monitoring ... 22

8.4 For-Cause Visits ... 23

8.5 Clinical Trial Close-Out Visit ... 24

9. Document Access Requirements For Participating Sites ... 26

9.1 File Naming Requirements For Scanned Documents ... 26

10. Monitoring Processess ... 27

10.1 Monitoring Reports And Action Items ... 27

10.2 Logging Monitoring Visits ... 27

11. Guidelines For Monitoring Of Serious Adverse Events ... 28

12. Essential Documents ... 28

13. Document Version History ... 29

1. ABBREVIATIONS

6mCDP	6-month Confirmed Disability Progression
9-HPT	9-Hole Peg Test
AE	Adverse Event
ANZCTR	Australian New Zealand Clinical Trials Registry
ARTG	Australian Register of Therapeutic Goods
BD	Twice a day
CDP	Confirmed Disability Progression
CI	Coordinating / Lead Site Principal Investigator
COV	Close-out Visit
CRF	Case Report Form – paper and electronic
CRO	Clinical Research Organisation
CTAC	Clinical Trials Advisory Committee
CTC	Clinical Trial Coordinator
CTM	Clinical Trial Manager
CTN	Clinical Trial Notification scheme
CV	Curriculum Vitae
DMT	Disease Modifying Therapy
DSMB	Data Safety Monitoring Board
EBNA1	Epstein-Barr Nuclear Antigen-1
EBV	Epstein-Barr Virus
eCRF	Electronic Case Report Form
ED	Essential Documents
EDSS	Expanded Disability Status Scale
EUC	Electrolytes, Urea and Creatinine
EQ-5D-5L	EuroQol – 5 Domains – 5 Levels
FBC	Full Blood Count
FLAIR	Fluid Attenuated Inversion Recovery
FSMCF	Fatigue Scale Motor and Cognitive Functions
GCP	Good Clinical Practice

Gd	Gadolinium
HREC	Human Research Ethics Committee
ICH	International Conference on Harmonisation
IMP	Investigational Medicinal Product
IMV	Interim Monitoring Visit
ISF	Investigator Site File
LFT	Liver Function Tests
MAMS	Multi-arm Multi-stage
MOP	Manual of Procedures
MRI	Magnetic Resonance Imaging
MS	Multiple Sclerosis
MSFC	Multiple Sclerosis Functional Composite
MSIS-29	Multiple Sclerosis Impact Scale -29
MSWS-12	Multiple Sclerosis Walking Scale -12
PI	Principal Investigator
PICF	Participant Information and Consent Form
PROM	Participant Reported Outcome Measure
RCT	Randomised Controlled Trial
RGO	Research Governance Office
SAE	Serious Adverse Event
SAR	Serious Adverse Reaction
SC	Study Coordinator
SDMT	Symbol Digit Modalities Test
SDV	Source Data Verification
SIV	Site Initiation Visit
SOC	Standard of Care
SOP	Standard Operating Procedure
SUSAR	Suspected Unexpected Serious Adverse Reaction
T25FW	Timed 25-Foot Walk
TGA	Therapeutics Goods Administration
TMG	Trial Management Group

TMT	Trial Management Team
TMF	Trial Master File

2. INTRODUCTION AND STUDY INFORMATION

Compliance with the Protocol is important for ensuring ethical conduct of the clinical trial and collection of accurate and complete data. The purpose of this monitoring plan is to define clinical trial-specific monitoring requirements to ensure compliance with the trial protocol, ethical guidelines, regulatory requirements, and data accuracy for the STOP-MS trial.

This Monitoring Plan establishes the guidelines for conducting monitoring visits and related tasks for monitoring STOP-MS trial; Phase III, multicentre, randomised, double-blinded, placebo-controlled, MAMS trial of SpironolacTone and famciclOvir in the treatment of Progressive MS to prevent disability progression (STOP-MS) and is a requirement of the Integrated Addendum to ICH E6 (R1): Guideline for Good Clinical Practice E6 (R2). Monitoring will be conducted for all participating sites across Australia to maintain the integrity of the trial's data and participant safety.

**2.1 INFORMATION ABOUT THE CLINICAL TRIAL**	
Clinical trial identifier	ANZCTR12623000849695P
Type of study	Clinical Trial of an investigational medicinal product (IMP)
Methodology	Phase III, Adaptive, multicenter, randomised, double-blinded, placebo-controlled trial (RCT)
Type of IMP/Type of Therapeutic Good:	Spironolactone: 50mg BD oral capsules<br>Famciclovir: 500mg BD oral capsules<br>Placebo: Matched placebo capsules, twice daily oral administration to enable blinding and allocation concealment
Treatment groups	Arm 1: Spironolactone + SOC<br>Arm 2: Famciclovir + SOC<br>Arm 3: Placebo + SOC
Study Population:	Adults with progressive MS (primary progressive or secondary progressive) either on current DMT or on no treatment.

Primary Objective:	Stage 1: Demonstrate that spironolactone or famciclovir plus SOC reduce the frequency of EBV DNA being present in saliva and/or reduce EBNA1 antibody titres in people with progressive MS when compared to placebo plus SOC.<br><br>Stage 2: Demonstrate that spironolactone or famciclovir plus SOC reduce the likelihood of 6-month CDP (6mCDP) in people with progressive MS when compared to placebo plus SOC.
Primary Outcome Measure:	Stage 1: Co-primary outcomes measures: frequency of salivary EBV DNA detection in monthly samples and serum EBNA1 antibody titres in serum at 6 months post-commencement of intervention.<br><br>Stage 2: Time to 6mCDP using a composite of EDSS, T25FW28 and 9-HPT.<br><br>Definitions of progression will be an increase in EDSS (of 1 point if baseline EDSS was <5.5, or 0.5 points if baseline EDSS was ≥5.5); ≥20% increase in 9-HPT time; or ≥20% increase in T25FW.
Secondary objective	To demonstrate that spironolactone or famciclovir plus SOC:<br>a. are safe when used to treat people with progressive MS<br>b. reduce the rate of brain atrophy at 3 years compared to placebo plus SOC

	c. reduce the numbers of new/expanded T2/ FLAIR and Gd-enhancing lesions on MRI brain compared to placebo plus SOC<br>d. reduce the level of whole brain atrophy on MRI brain compared to placebo plus SOC<br>e. improve PROMs of disease impact compared to placebo plus SOC.<br>f. are cost-effective.
Secondary outcome measure	Clinical – time to first relapse, time to 6mCDP using EDSS only, MSFC Score.<br>MRI – new and enlarging lesion counts.<br>PROMs – MSIS-29, MSWS-12, Neuropathic Pain Scale and FSMCF.<br>Health economics – EQ-5D-5L.
Number of participants (for all sites)	Stage 1: n=150 (50 per arm – 6 months)<br>Stage 2: n=200 (2X 100 per arm; placebo + most effective treatment – 3 yrs)<br>Total = 350
CI	Prof Simon Broadley
Number of sites (planned)	Up to 22 (10 are expected)

2.2 STUDY SITES

	SITE NAME	SITE ADDRESS	PI
**1.**	Griffith University	School of Medicine and Dentistry,<br>Gold Coast Campus,<br>Griffith University QLD 4222	Prof Simon Broadley
**2.**	Concord Hospital (Sydney)	Neurology Department,<br>Concord Hospital,<br>Concord West NSW 2139	A/Prof Todd Hardy/ A/Prof Sudarshi Ramanathan

**3.**	John Hunter Hospital (Newcastle)	John Hunter Hospital,<br>Neurology Dept,<br>New Lambton Heights NSW 2305	Prof Jeannette Lechner-Scott
**4.**	Mater Hospital Brisbane	Mater Health and Mater Centre for Neurosciences,<br>Mater Hospital Brisbane,<br>Raymond Tce, South Brisbane 4101	Dr Andrew Swayne
**5.**	Lyell McEwin Hospital	Lyell McEwin Hospital,<br>OPD2 Haydown Rd,<br>Elizabeth Vale SA 5112	Dr Deborah Field
**6.**	The Alfred (Melbourne)	L6 (Clinical Neurosciences),<br>99 Commercial Rd,<br>Melbourne VIC 3004	A/Prof Anneke van der Walt/ Prof Helmet Butzkueven
**7.**	The Austin (Melbourne)	Austin Hospital,<br>145 Studley Rd,<br>Heidelberg VIC 3084	Dr Marion Simpson/ Prof Richard Macdonell
**8.**	Royal Melbourne Hospital	The Royal Melbourne Hospital,<br>Neuroimmunology Centre,<br>L7, 635 Elizabeth St,<br>Melbourne VIC 3000	Dr Vivien Li
**9.**	Perron Institute (Perth)	Perron Institute for Neurological and Translational Science,<br>8 Verdun St,<br>Nedlands WA 6009	Prof William Carroll/ Prof Allan Kermode
**10.**	Brain and Mind Centre (University of Sydney)	Brain and Mind Centre,<br>94 Mallett St,<br>Camperdown NSW 2050	Prof Michael Barnett
**11.**	Launceston General Hospital	Clifford Craig Foundation,<br>Launceston Hospital,<br>274-280 Charles St,<br>Launceston TAS 7250	Dr Lauren Giles

**12.**	Menzies Institute for Medical Research	Menzies Institute for Medical Research,<br>17 Liverpool St,<br>Hobart TAS 7000	Prof Bruce Taylor
**13.**	Box Hill Hospital/Eastern Health Building	Box Hill Hospital Eastern Health Bldg<br>8 Arnold St, 5 Arnold St<br>Box Hill VIC 3128 Box Hill VIC 3128	Dr Katherine Buzzard
**14.**	Liverpool Hospital	Neurology Department,<br>L1 Mental Health Building,<br>Elizabeth St,<br>Liverpool NSW 2170	A/Prof Suzanne Hodgkinson
**15.**	Royal Brisbane and Women's hospital	Neurology OPD,<br>Royal Brisbane and Women's Hospital,<br>Cnr Butterfield St and<br>Bowen Bridge Rd<br>Herston QLD 4029	Dr Zara Ioannides
**16.**	Monash Medical Centre	Monash Medical Centre,<br>246 Clayton Rd,<br>Clayton VIC 3168	Dr Nevin John
**17.**	Barwon Health	Neuroscience Department,<br>Barwon Health,<br>Bellerine St, Geelong VIC 3220	Dr Cameron Shaw
**18.**	Royal North Shore Hospital	MS Unit, Neurology Department,<br>Royal North Shore Hospital,<br>Reserve Rd, St Leonard's NSW 2065	Dr John Parratt
**19.**	Sunshine Coast University Hospital	Neurology Department,<br>Sunshine Coast University Hospital,<br>6 Doherty St,<br>Birtinya QLD 4575	Dr Joshua Barton
**20.**	Flinders Medical Centre	Department of Neurology<br>Flinders Dr,<br>Bedford Park SA 5042	Dr Lesly-Ann Hall

**21.**	Princess Alexandra Hospital	Department of Neurology<br>199 Ipswich Rd<br>Woolloongabba QLD 4102	Dr Laura Clarke
**22.**	Westmead Hospital	Department of Neurology,<br>Cnr Hawkesbury Rd and Darcy Rd,<br>Westmead NSW 2145	Dr Andrew Henderson

**2.3 CLINICAL TRIAL – SPECIFIC INFORMATION FOR MONITORING**	
Planned number/frequency of monitoring visits	Remote SIV. IMV after recruitment of first 3 participants at each site. All subsequent IMVs will take place every 6 months unless a more frequent monitoring schedule is identified as necessary
Expected duration of each monitoring visit	1 day
Time of first monitoring visit at a site in relation to first participant first visit	Once site has enrolled 3 patients
Time of close-out visit at a site in relation to last participant last visit	After the final participant visit and all data has been entered into CRF.
IMPs for investigation	Famciclovir and Spironolactone
Comparator	Placebo
Blinding method	Double-blinded study

3. MONITORING OBJECTIVES

Monitoring is designed to:

- help ensure that the rights, safety and wellbeing of human subjects enrolled in a clinical trial are protected.
- have an objective assessment of the Investigator's adherence to the approved Protocol and amendments.
- confirm adherence to all applicable government regulations, HREC approval and to ICH GCP guidelines.
- validate the integrity of the data collection and recording process.

4. ROLES AND RESPONSIBILITIES

This trial will be monitored by a CRA employed by Griffith University. The appointed Monitor will be qualified by education and experience to monitor the trial conduct at each site is being conducted according to applicable SOPs (STOP-MS-SOP-001 – Investigator Site File Maintenance; STOP-MS-SOP 003 – Documentation of Protocol Deviations; STOP-MS-SOP-006 – Source Documentation; STOP-MS-SOP-008 – Defining Roles and Responsibilities; STOP-MS SOP-009 – Obtaining Informed Consent; STOP-MS SOP-010 – Managing Training; STOP-MS SOP-011 – Unblinding of Investigational Product; STOP-MS SOP-013 – Destruction of Investigational Product; ICH GCP and local requirements.

4.1 CRA RESPONSIBILITIES

4.1.2 GENERAL OVERSIGHT AND PROTOCOL ADHERENCE

At each Monitoring Visit, the Monitor should confirm the continued ability and commitment of the PI and site staff to conduct the trial. This includes the following tasks:

1. The CRA will verify that the PI adheres to the approved protocol, amendments, Good Clinical Practice (GCP), and relevant regulatory requirements while staying well-informed about the trial's progress. Any deviations will be reported to the CI, with appropriate action designed to prevent recurrence of the detected deviations taken.
2. The CRA will verify that the PI and their team have the necessary qualifications, resources, and facilities, including laboratories, equipment, and adequately trained staff, to conduct the trial safely and properly. This must be maintained throughout the trial.

3. The CRA will ensure that trial responsibilities are performed as designated and not delegated to unauthorised personnel by reviewing the Delegations Log and Training Log to ensure it is complete, current and delegation is in accordance with qualifications and training.
4. The CRA will ensure that informed consent is obtained and documented before any participant takes part in the trial, and only eligible participants are enrolled as outlined in the trial's protocol.
5. Ascertain participant recruitment rates and ensure enrolment aligns with protocol requirements.
6. Verify that the PI is providing adequate supervision to any individual or party to whom they have delegated trial-related duties and functions. Evidence of supervision may include email correspondence and meeting minutes with attendees listed.
7. The CRA will ensure that the source data location list is up-to-date and reflects current practices.

4.1.3 DATA AND DOCUMENTATION VERIFICATION

Source data verification entails checking that all entries in CRFs are consistent with data in the participant records or other source data for the purpose of confirming the trial is being conducted according to the Protocol and applicable regulations, including GCP, and confirming accurate reporting of participant safety data and trial endpoints. A check is made of whether all information relevant to the trial has been transferred to the CRF.

1. The CRA will verify that source documents and other trial records are accurate, complete and current, ensuring the CRF entries align with these records.
2. The CRA will confirm that Informed consent was obtained appropriately – as described in the trial Protocol.
3. The participants enrolled in the trial meet the Protocol criteria for eligibility.
4. With respect to the CRFs, the CRA will verify that:
    a) All outcome data required by the protocol is accurately recorded in the CRFs and matches the source documents.
    b) Any dose modifications and the reason for the dose modification for the investigational product are documented for each participant in both the medical record and the CRF.

c) Concomitant medications and concurrent illnesses are reported as per the protocol.

d) Adverse events/SAEs/SARs/SUSARs are accurately recorded in source documents and reported within specified timeframes.

e) Missed visits, unperformed tests, and skipped examinations are clearly documented in the CRFs and recorded as protocol deviations.

f) All withdrawals and dropouts of enrolled participants from the trial are reported and explained on the CRFs.

5. The CRA will inform the PI of any errors, omissions, or illegible entries in the CRF and ensure these are corrected, dated, explained (if needed), and initialled by the investigator or an authorised team member. The CRA is not allowed to make these changes directly. If the CRFs are paper the corrections shall be dated and initialled. Each parameter that is not recorded is a missing value. Write ND (Not Done) where procedures have not been carried out. If data is missing for a whole page of the CRF, a diagonal line shall be drawn through the whole page, and the letters "ND". The reason for missing data may be that the trial Participant has withdrawn from the trial or has not come for some of the check-ups.

4.1.4 INVESTIGATIONAL PRODUCT (IMP) ACCOUNTABILITY

The Monitor will be an unblinded monitor and will therefore not be involved in any aspect of participant visits.

At each IMV, the Monitor will verify the following:
Reconciliation of the accounts for received, dispensed, returned and destroyed IMPs against the trial site's stock of unused and returned IMPs. The following details should be checked:

1. Participant number.
2. Delivery date / return date.
3. Number of delivered / returned units.
4. Check of visit interval against actual use.
5. Storage conditions meet Investigator's Brochure / product information requirements
6. Supplies are sufficient.
7. That participants are provided with necessary instruction on properly using, handling, storing and returning the investigational product/s.

8. IMPs are administered only to eligible participants, at the correct doses, and according to the randomised treatment allocations.
9. IMP receipt, use, and return are adequately controlled and documented.
10. That the disposal of unused investigational product/s at the trial sites complies with applicable regulatory requirement/s and is in accordance with the Sponsor's requirements (e.g. Protocol and sops describing process for Investigational Product Accountability).
11. The CRA will confirm that the trial's blinding is maintained and that any code breaks are handled and documented following the protocol and relevant SOPs.
12. If the CRA suspects scientific misconduct, fraud, or GCP breaches, they will immediately inform the Chief Investigator (CI). The issue will then be addressed per organisational policies.

5. RISK CATEGORY FOR THE TRIAL

Type B – Risk associated with modified use of an existing product.

- Trials involving a drug entered onto the ARTG and such products are used for a new indication (different population/disease group).
- Requires moderate intensity monitoring using on-site, remote, and statistical monitoring strategies.
    - Site Initiation Visit (SIV) conducted in in-person or online.
    - First Interim Monitoring Visit (IMV) conducted in person or online (if appropriate).
    - Subsequent IMV conducted in person or online (if appropriate).
    - Close Out Visit (COV) conducted in person or online (if appropriate).
- Requires monitoring of key data protocol adherence and data quality:
    - 100% informed consent process review for all eligible participants
    - 100% SAE or AEs of interest
    - 100% eligibility of all participants
    - 100% SDV of primary critical data and critical processes for first 3 enrolled participants per site.
    - 100% SDV of primary critical data and processes for 20% selected participants after first IMV

- Additional site visits may be triggered by concerns identified from central monitoring that cannot be address by other means.

6. MONITORING COMMUNICATION PLAN

The monitor will send monitoring communication, including site visit confirmation emails, agendas, follow up emails, etc, to the lead Clinical Trial Manager (CTM) as well as to relevant site PIs and Clinical Trial Coordinators (CTCs). Monitoring Reports will be sent to Griffith University Office for Research and Prof Simon Broadley as CI.

The monitor will work with the CI, PI and primary contact to schedule monitoring visits. The CI will be informed of visit scheduling at participating sites.

Prior to the visit, the PI will receive a visit confirmation email providing a brief agenda, a list of participant files to be monitored and the expected time required for the Monitoring Visit. The Monitor will ensure that this information is communicated to the site personnel within a mutually agreed upon timeframe to allow sufficient time for recording requests. The PI and research staff will be expected to secure a workspace for the Monitor and to be available during the visits to facilitate Monitoring activities.

The Monitor and site staff should be available during each monitoring visit to discuss any findings and answer questions.

7. CRITICAL ROLES, PROCESSES AND DATA

At each monitoring visit, the Monitor will Review the Delegations Log and Training Log to ensure it is complete, current and delegation is in accordance with qualifications and training.

Critical Delegated roles

- Coordinating Principal Investigator
- Clinical Trial Manager
- Site Principal Investigators
- Associate Investigators
- Clinical Trial Pharmacists
- Clinical Research Coordinators

At each monitoring visit, the Monitor will verify the following critical processes:

- **Assessment of patient eligibility:** Ensuring participants meet specific inclusion and exclusion criteria for progressive MS and EBV infection.
- **Informed Consent:** Participants provide written informed consent using current applicable informed consent form (screening, intervention, biobanking).
- **Randomisation:** Maintaining the integrity of the double-blind, placebo-controlled design.
- Study Drug Administration: Accurate dosing and adherence to the study drug regimen (spironolactone and famciclovir).
- **Outcome Assessment:** Standardised evaluation of disability progression using the Expanded Disability Status Scale (EDSS).
- **Safety Monitoring:** Thorough collection and reporting of adverse events (AEs), particularly those related to the study drugs.
- **Investigational Medicinal Product accountability:** Manufacture, storage, handling, and accountability.

At each monitoring visit, the Monitor will verify the following critical data:

CRITICAL DATA	SOURCES
Co-primary outcomes measures: frequency of salivary EBV DNA detection in monthly samples and serum EBNA1 antibody titres in serum at 6 months post-commencement of intervention	Pathology results: – full blood count (FBC), electrolytes, urea creatinine (EUC), eGFR, liver function tests (LFT) and EBNA1 titres.
Time to 6mCDP using a composite of EDSS, T25FW and 9-HPT.	Source document records reporting the following outcome measures - Time to 6mCDP: EDSS, MS Functional Composite (MSFC) including T25FW, 9-HPT and SDMT.
All AEs and SAEs reported where the cause has been determined to be related to the study treatment.	Patient medical records recording medical history, concomitant medications, adverse events/SAEs/SARs/SUSARs.

Randomisation records.	RedCap randomisation output and confirmation of participant assignment to correct allocation.
Study drug compliance.	Participant study drug compliance record and Investigational Medicinal Product accountability records.

8. TYPES OF VISITS AND MONITORING ACTIVITIES

8.1 SITE INITIATION VISIT (SIV) (ONLINE)

Once all regulatory documents and approvals are received, a site initiation visit via Teams/Zoom will be scheduled. During this visit, the Monitor will review the following with the PI and his/her staff as appropriate:

- Trial goals and obligations including
  - Verify that the PI understands and accepts responsibility for overseeing the conduct of the study in accordance with the protocol, applicable regulations and GCP, as well as ensuring the conduct of all staff performing study procedures.
  - Ensuring staff performing assessments have appropriate training and required certification. Some assessments (i.e. EDSS and FMSC) may be performed by non-neurologists, but staff must be specifically trained in these tests.
  - Verify that the PI understands and accepts the responsibility to obtain HREC/RGO approval of any amended protocols, consent documents, or advertisements, and to ensure continuing review of this study by the HREC/RGO.
- Protocol procedures
  - Review study objectives, study design and study population
  - Review study inclusion/exclusion criteria
  - Review participant randomisation process
  - Review study schedule of events and sample collection requirements
  - Review protocol required clinical and laboratory assessments (including EDSS, T25FW and 9-HPT)
  - Review responsibility to review, sign, and follow-up on laboratory reports

- Review process for premature discontinuation of study participants

- Review of facilities – PI to confirm appropriate spaces are available for consent discussions, participant visits, laboratory specimen collection, processing, and storage, records and ISF management, and monitoring workspace.
- Informed consent procedure
  - Discuss the site's informed consent procedures.
  - Verify that the PI understands and accepts the responsibility to obtain informed consent in accordance with all applicable regulations and to document the informed consent process for each participant.
- Manual of Procedures (MOP)/ Standard Operating Procedures (SOP)
  - Review to ensure understanding of the necessity of standardisation of protocol execution across all relevant study team members.
  - In the absence of a MOP, review the applicable site SOPs.
- Investigator site file
  - Verify that all study documents are present in the ISF and make the PI and site personnel aware of their responsibility to keep the file complete and current.
  - Verify that the Delegation of Responsibilities Log is current and signed by the PI.
  - Review maintenance requirements of the investigator binder and site visit log
- Electronic Case Report Form (eCRF) and Laboratory tracking
  - Confirm site personnel have received training on the use of the electronic data capture system, paper-based CRF worksheets and the specimen management and tracking system for the study.
  - Review and provide further instruction for eCRF completion as required.
  - Ensure that the PI and site personnel are aware of eCRF correction and data clarification requirements.
- Study documentation
  - Verify that the PI understands that he/she is responsible for retaining all study records and making them available for monitoring and audits during the conduct of the study and throughout the retention period.
  - Review the document retention requirement for all study-related records. Inform the PI that all study records must be retained for a 15-year archiving period.

- Review requirements for maintaining adequate source documentation that supports the data recorded in the eCRFs.

- Safety Reporting
  - Discuss adverse event (AE) and serious adverse event (SAE) grading, causality, reporting and review.
  - Review requirements for HREC/RGO/Sponsor notification of AEs, and SAEs.
- Laboratory supplies and procedures
  - Verify that the site has adequate supplies available as detailed in Site procedure documents
  - Review collection, handling, storage, and transport procedures for laboratory samples (including salivary EBV DNA samples and serum EBNA1 samples).
  - Discuss Site-Level Quality Management Activities
  - Laboratory supplies and procedures

- Document Access Requirements
  - Verify that the PI understands that Sites must ensure that clinical trial monitors have timely and appropriate access to participant and trial documentation.
  - The access protocol depends on the record-keeping system and CRF in use at the site.
- Investigational Medicinal Product
  - Review requirements for drug storage and accountability.
- Discussion of general items
  - Discuss enrolment goals and timelines.
  - Obtain documentation of all site personnel present for the SIV on the SIV Training Log.
  - Ensure that all required supplies/clinical trial materials have been received by the clinical study site prior to screening or enrolling the first study participant.
  - Discuss the expected schedule of monitoring visits with site personnel, including the timing of the first monitoring visit, personnel availability, and monitoring space availability.
  - Initiate discussion of site close-out procedures. Study close-out procedures will be discussed in further detail during IMVs.
- Any other issue as deemed important to the conduct of the trial.

8.2 INTERIM MONITORING VISITS (IMV)

The first IMV will take place once three patients have been recruited at a site. This is an important visit as it allows any errors to be addressed / not repeated. IMVs should be conducted onsite but may be conducted remotely if monitor and PI are agreeable and systems are in place to share documentation adequately and securely.

Monitoring Visits shall be coordinated with the trial site staff to enable trial personnel to be available during the monitoring visit and to ensure source documentation is available for review.

The purpose of Monitoring Visits is to verify that:

- the PI is conducting the trial in accordance with the Protocol, applicable SOPs, GCP and applicable regulatory requirements
- participants' safety, rights and wellbeing are being protected
- data recorded on the CRFs are accurate, complete and verifiable from source documentation.

During the visit the following should be reviewed:

- Informed consent process for each participant, 100% verification
  a) Ensure all participants have signed and dated an HREC approved consent form including reconsent if applicable. Ensure there is documentation confirming that the trial was explained to the participant and that consent was obtained before any trial procedures were carried out.,
  b) The correct version of consent documents was used at the time of consent and that revised versions of the consent form are signed and dated if applicable. Deviations to the informed consent process are documented and all applicable parties are informed.
  c) Ensure the date of consent correlates with a visit date, either before or on the day of the first study-related activity.
  d) The participant/parent/guardian/substitute health attorney/person responsible have personally written their own names, signed and dated the consent form.
  e) In cases where the participant/parent/guardian/substitute health attorney/person responsible is illiterate, an impartial witness has signed and dated the consent form. The

impartial witness cannot be an interpreter providing interpreter services during the consent process.

f) Ensure medical records document the process of informed consent, a signed copy is filed in the participant site file or ISF and a copy of the signed PICF has been given to the participant/parent/guardian.

- Source document verification; 100% at first IMV, 20% at subsequent IMVs
- CRF completion
- Patient eligibility, 100% verification
- Investigational product accountability
- Check and review of the trial binder and all essential documents
- Clinical supply inventory
- SAE reporting, 100% verification
- Significant Protocol violations and deviations
- Enrolment issues and targets
- Any protocol amendments and their approval by the HREC
- Acceptability of facilities
- Personnel changes
- Delegation and Training Logs
- Updated regulatory documentation
- Any other issue as deemed important to the conduct of the trial.

All subsequent IMVs will take place every six months unless a more frequent monitoring schedule is identified as necessary. Source document verification and patient eligibility verification can be reduced to 20% of new documents/participants after the first IMV. This is subject to no significant findings during the first IMV.

8.3 STATISTICAL AND REMOTE MONITORING

When electronic CRFs (eCRFs) become accessible, the monitor will review the entries and REDCap reports to ensure data completeness, with particular attention to sites showing a higher rate of protocol deviations and screen failures. This remote monitoring will be conducted monthly.

The Monitor will analyse accumulating study data using REDCap data quality modules to detect trends, outliers, or anomalies that might indicate issues with data quality, protocol adherence, or participant

safety. This proactive approach uses statistical methods and data visualisation tools to identify patterns or deviations from expected norms. Each monitoring occasion will focus on specific data points or study aspects with higher potential risks. The goal is to identify and address potential problems early, improving the overall quality and reliability of clinical trial data.

8.4 FOR-CAUSE VISITS

For-cause visits may be required to address any unanticipated issues that arise which require training, remediation or other situations for which the site requires assistance. All for-cause visits should be notified to the Sponsor prior to the visit. For-cause visits can be requested by the Sponsor or can be requested by the site. These visits may involve either Onsite Monitoring or Remote Monitoring if applicable.

Examples of triggers for for-cause visits include:

FOR-CAUSE VISIT TRIGGER	HOW IS TRIGGER IDENTIFIED	MONITORING CHECK TO BE PERFORMED AT TRIGGERED VISIT
Data return rate of < 80% for a period of > 3 months	Report from REDCap generated and reviewed by Monitor	Review of patient's source data to ensure visits are being completed as per protocol requirements
Poor data quality defined as > 50% open queries for a period of >3 months	Report from REDCap generated and reviewed by Monitor	Review of all outstanding queries.
Poor quality data for primary outcome for stage 1 of 2 analysis	Report from REDCap generated and reviewed by Monitor	Review of all outstanding queries.
Repeated instances of late reported SAEs in the last quarter	Report from REDCap generated and reviewed by the Monitor	Review of patient's source data to ensure no further SAEs have been missed.

Repeated reports of a critical protocol deviation or after a single report of a serious breach at site	Protocol deviation or serious breach reported to CI, PI or Griffith University's Office for Research.	Review of patient's source data to ensure no further critical deviations or serious breaches have occurred.
Concerns raised by TMG or TMT that are thought to require further investigation	TMG and TMT minutes	Review of patient's source data

8.5 CLINICAL TRIAL CLOSE-OUT VISIT

COV is only done after all CRFs have been completed and data management has closed out all queries. A close-out visit will be conducted to ensure appropriate documentation is present and complete. At the close-out visit, all files should be up to date and all trial documents or copies of those which are to go to the Sponsor should be collected.

Study closure activities may require more than one visit to ensure the proper closure of the study. These activities may be conducted during a series of on-site visits or by telephone. Close-out visits may be conducted at study completion or earlier in the case of study termination by the HREC, DSMB or another Regulatory Body.

The outcome of the visit and other close-out activities will be documented in a report and follow-up letter.

Monitor will perform the activities below during the study close-out process:

a) Consent Documents (for consents not checked since previous monitoring visit)

- Confirm that consent was obtained for each participant prior to initiating study activities.
- Confirm that consents contain appropriate signatures and dates.
- Confirm that the correct version of the consent document was signed and dated.
- Confirm that additional consent was obtained for protocol amendments as required by the site's HREC/RGO.

b) Investigator Site File

- Ensure that essential document files are complete and current (See essential documents listing in this Monitoring Plan).

- Identify any missing study documents.
- Confirm with PI that all essential documents must be retained for a minimum of 15 years.
- Ensure that the Authorised Signature and Delegation Logs are complete and signed by the PI.

c) Source Documentation and CRF Review

- Reconcile the final status of all participants listed on the screening log.
- Confirm that all required data fields have been verified against source.
- Confirm that all data queries have been resolved.
- Confirm that the PI has reviewed, signed, and dated all required CRF pages
- Verify that the site has legible copies of all CRFs
- Confirm that protocol deviations are noted in the source documents.

d) Unanticipated Problems, Adverse Events, and Serious Adverse Events

- Confirm that all AEs, and SAEs have been reported to the appropriate regulatory agencies as required.
- Confirm that the site has and will continue to meet safety reporting requirements.
- Ensure that copies of SAE reports are filed with the corresponding site files.

e) Investigational Medicinal Product

- Confirm that all investigational product accountability records have been maintained appropriately and are consistent with the amount of remaining product.
- Ensure that remaining IMP will be returned or destroyed per institutional requirements. Document proper destruction of any remaining product.

f) Laboratory Samples

- Confirm that all lab samples have either been analysed or stored for future analyses.
- Confirm future use specimen disposition and labelling/de-identification, as appropriate.
- Confirm site process for identification and disposition of future use samples connected to participants who withdraw consent.

g) Regulatory Obligations

  - Confirm that the PI has met and will continue to meet regulatory obligations.
  - Confirm that the PI has provided written notification of study closure to the HREC/RGO and verify acknowledgement by the HREC/RGO of study closure.
  - If the study was terminated prematurely, the Monitor will confirm that enrolled participants were informed, and that appropriate therapy and follow-up was initiated by the PI.

- Inform the PI of the possibility of future audits by regulatory authorities.

h) Records Retention

- All clinical trial records archived for 15 years

At the conclusion of the COV, the Monitor will meet with the PI and site SC to discuss:

- Any findings noted during the visit.
- Retention timeframes for study-related documents.
- Safety reporting requirements.
- Notification of the HREC/RGO that the study has concluded.
- Outstanding issues at study closure and a plan for their resolution.
- A final Report and close-out request will be submitted to the HREC/RGO to close all sites.

9. DOCUMENT ACCESS REQUIREMENTS FOR PARTICIPATING SITES

Sites must ensure that clinical trial monitors have timely and appropriate access to participant and trial documentation. The access protocol depends on the record-keeping system and CRFs in use at the site:

1. **Digital Record System:**
    - Provide the clinical trial monitor with access to digital records and eCRFs before the monitoring visit.
2. **Paper Copies:**
    - Ensure all trial files are scanned and stored in a SharePoint or OneDrive folder, accessible to the clinical trial monitor at the time of the monitoring visit.
    - If no SharePoint or OneDrive folder exists, create one specifically for this purpose.
    - If scanning all files is not feasible, staff must be available during the monitoring visit to present documents via video call.

9.1 FILE NAMING REQUIREMENTS FOR SCANNED DOCUMENTS

When uploading scanned documents, all files must be named in the following format to ensure consistency and ease of identification:

Site Number _ParticipantID_Initials_File Type

- **SITE NUMBER:** The unique identifier for the site (e.g., 1234, 1235).
- **ParticipantID:** The unique ID assigned to the participant.
- **Initials:** The participant's initials.
- **FILE TYPE:** The document type (e.g., PICF for Participant Informed Consent Form)

Example

For a participant with the ID "001" and initials "ABC" from site "1234," a Participant Informed Consent Form would be uploaded as:

1234_001_ABC_PICF

10. MONITORING PROCESSESS

10.1 MONITORING REPORTS AND ACTION ITEMS

Monitoring Visit findings and resulting action items will be documented in Monitoring Visit Reports. The Monitor will provide a written Monitoring Visit Report to the CI and provide a follow up letter to site trial team members within 10 business days of any visit. The Monitoring Visit Report is not for distribution to the site and should be signed by the CI and filed in the TMF only. A copy of the Monitoring Report should also be submitted to Griffith University's Office for Research and Vanessa Vigar as Clinical trial manager.

The follow up letter should be signed by the site PI and filed in the ISF and TMF.

The Monitor will work with designated site staff to resolve any outstanding action items as communicated in the follow up letter.

10.2 LOGGING MONITORING VISITS

The monitor will maintain a Monitoring Visit Tracking document within the Trial Master File (TMF) to record all site visits. This record will include the date of each visit, the date the monitoring visit report was sent to the site and resolved, and the number of critical and major findings identified.

Additionally, each site's Investigator Site File (ISF) must include a site-specific monitoring visit log that documents all visits to that site. This log should be signed by both the attending monitor and a site representative at the end of the visit.

11. GUIDELINES FOR MONITORING OF SERIOUS ADVERSE EVENTS

SAEs must be reported to Griffith University's Office for Research and to Prof Simon Broadley, irrespective of the site where it occurred. Reports of SAEs shall also be submitted to the Research Governance Office of the site where it has occurred. The PI is responsible for informing the Sponsor of the adverse events related to the trial, not just the trial drug. The Monitor shall ensure consistency between entries in the SAE form, the participant records and the CRFs. Where the clinical trial involves the administration of a registered product, the PI is responsible for reporting the adverse events to the Therapeutic Goods Administration via their website. Monitor to check for adherence to this requirement.

12. ESSENTIAL DOCUMENTS

Essential Documents (EDs) should be maintained at each trial site and serve as the central source for ED maintenance at the site. Documents may be kept electronically and/or as paper documents. The Monitor will review the files for accuracy and completeness. Any discrepancies or missing documents should be discussed with the site staff.

The following documents are to be maintained in the files:

- The HREC-approved Protocol and any amendments to the Protocol, Protocol Amendment/s and Signature Pages, sample CRF.
- All versions of the informed consent, advertisements for participant recruitment, diaries, questionnaires and trial documents provided to families.
- Investigator's Brochure/Product Information and any amendments
- Annual Reports, Annual Safety Reports, expedited safety reports, serious breach reports, SAE reports, notification of changes to the trial team.
- All correspondence between the CI / PI and HREC / local Governance Office, as appropriate. This includes submissions, approvals and responses to questions / comments.
- Regulatory Documents (copies of all TGA correspondence, e.g. submission of CTN, CTN acknowledgement, CTN completion).
- Documented evidence (e.g. Note to File) and reporting of non-compliance to GCP, SOPs, Protocol to Sponsor.

- PI and Associate Investigator / Sub-Investigator Curriculum Vitae (CV) signed and dated within 2 years.
- Delegation Log – up to date, all tasks appropriately delegated.
- Training Log – incudes trial-specific and GCP, valid for duration of involvement in trial.
- Screen Log, listing all participants with a signed informed consent and, if the participant was randomised / treated. For any participant not randomised / treated, the reason they were not randomised / treated.
- List of trial participants – list will remain at site and will not be copied. The list also functions as the participant identity list for the trial.
- Monitoring Log.
- Trial Contact List.
- All completed CRFs (and copies) appropriately filed (originals should have been collected for the sponsor; [may be electronic CRFs]).
- Legal documentation and agreements (e.g. Clinical Trial Research Agreement, Confidentiality Agreement).
- IMP reconciliation records are completed and appropriately filed
- IMP shipment and return invoices are present and appropriately filed
- Laboratory certification and reference ranges
- Finance and budget documentation.

13. DOCUMENT VERSION HISTORY

TITLE	Clinical Trials Monitoring Plan			
VERSION	AUTHOR	MODIFICATIONS MADE	DATE	STATUS
1.0	Vanessa Vigar/Lidia Madrid	Administrative updates only.	07 Jan 2025	Final
