# Supplementary material for: Study protocol for a multicentre, randomised, double-blinded, placebo-controlled, multi-arm, multi-stage, trial of SpironolacTone and famciclOovir in the treatment of Progressive Multiple Sclerosis to prevent disability progression: the STOP-MS trial
Source: BMJ Neurol Open. 2025 Dec 23;7(2):e001313. doi: 10.1136/bmjno-2025-001313 (PMC12730750; doi:10.1136/bmjno-2025-001313)
Supplement: online supplemental file 5 [file bmjno-7-2-s005.pdf]

Site #	____	____	____	____	Subj #	____	____	____	Initials	____	____	____

STOP-MS TRIAL

Screening

Consent Form

**Informed Consent Provided**	Yes ☐ No ☐
**Date of Consent** (dd/mm/yyyy)	___/___/______
**Time of Consent** (hh:mm) 24 hour	____ : ____
**Consenting Processes Completed**	
**Has read consent form** (if no then informed consent has not been obtained)	Yes ☐ No ☐
**Understands study** (if no then informed consent has not been obtained)	Yes ☐ No ☐
**Opportunity to ask questions** (if no then informed consent has not been obtained)	Yes ☐ No ☐
**Understands participation is voluntary** (if no then informed consent has not been obtained)	Yes ☐ No ☐
**Consent to EBNA1 antibody testing** (if no then informed consent has not been obtained)	Yes ☐ No ☐
**Understands copy of signed consent to be provided** (if no then informed consent has not been obtained)	Yes ☐ No ☐
**Release of medical information** (if no then informed consent has not been obtained)	Yes ☐ No ☐

Site #	____	____	____	____	Subj #	____	____	____	Initials	____	____	____

Opt-in choices	
**DNA sample and analysis**	Yes ☐ No ☐
**Serum sample biobanking and analysis**	Yes ☐ No ☐
**Future studies on biobanked samples (DNA and Serum)**	Yes ☐ No ☐
**Consent to biobank results being shared with collaborators**	Yes ☐ No ☐
**Consent to MSReactor**	Yes ☐ No ☐
**Consent to MRI collection**	Yes ☐ No ☐
**Consent to future use of MRI**	Yes ☐ No ☐
**Request Study Lay Summary**	Yes ☐ No ☐
**Contact details for Study Lay Summary** (email or postal address)	
**Name of person who undertook consenting process?**	______________________

Signed ________________________________ Date ________________

STOP-MS TRIAL

Screening

Demographic Details

**Site ID #**	_______	_______	_______	_______
**Participant ID #**	_______	_______	_______	
**Initials** (use 'X' if no middle or last initial)	_______	_______	_______	
**Sex**	Female ☐	Male ☐	Other ☐	
**Age**	_________ (Years)			
**Age Band**	25 – 39 Years	☐		
	40 – 54 Years	☐		
	55 – 70 Years	☐		
**Ethnic Ancestry** (can choose max of two)	☐ European<br>☐ Asian<br>☐ Aboriginal & Torres Strait Islander<br>☐ Māori<br>☐ American<br>☐ Middle Eastern<br>☐ African<br>☐ Other _________________________			
**First Name**	____________________			
**Email Address**	________________________________			

Signed ___________________________________ Date _________________

Site #	____	____	____	____	Subj #	____	____	____	Initials	____	____	____

STOP-MS TRIAL

Screening

Prior Relapse History

Complete one sheet for each prior relapse

**Relapse Number**		_____
**Date of Relapse Onset**		
**Day** (only if known)	**Month** (only if known)	**Year**
_____	_____	__________
**Duration of Relapse** (days)		____ days
**IVMP** (intravenous methylprednisolone administered)		Yes ☐ No ☐
**Recovery**		Full ☐ Partial ☐ None ☐
**Systems Affected**		Vision ☐
Brainstem ☐	Pyramidal ☐	Sensory ☐
Cerebellar ☐	Bladder & Bowel ☐	Mood & Cognition ☐
**Clinical Details**		

Signed ____________________________________ Date __________________

STOP-MS TRIAL

Screening

Medical History

Smoking History		
Never Smoker ☐	Ex-Smoker ☐	Current Smoker ☐
**If Ex- or Current Smoker**		

Year started smoking	______________
Year ceased smoking	______________
Habit (cigarettes/roll ups or grams per day)	______________
Cigarettes per day ☐	Grams tobacco per day ☐<br>(roll your own or pipe)

Alcohol	
Standard drinks per week	________ /week

**Height**	________________cm

Guide to Standard Drinks

**Beer**	Strength		
Size	Full	Mid	Low
Pot	1.1	0.8	0.6
Bottle/can	1.4	1.0	0.8
Pint	1.6	1.2	0.9
Case	34	24	19

**Drink**	Size						
	30ml	60ml	100ml	150ml	750ml	2L	4L
Champagne				1.4	7.1		
White Wine			0.9	1.4	6.8	18	36
Red Wine			1.0	1.6	8	21	43
Port		0.9				28	
Spirits	1.0				22		

**Pre-mixed spirits**	Size			
	250	300	375	440
5.0%	1.0	1.2	1.5	1.7
7.0%	1.5	1.6	2.1	2.4

Past Medical History		
**Condition**	**Year**	**Ongoing?**
		Yes □ No □
		Yes □ No □
		Yes □ No □
		Yes □ No □
		Yes □ No □
		Yes □ No □
		Yes □ No □
		Yes □ No □
		Yes □ No □
		Yes □ No □

Surgical History	
**Operation**	**Year**

Signed ____________________________________ Date __________________

Site #	____	____	____	____	Subj #	____	____	____	Initials	____	____	____

**Current Treatment**				
**Drug**	**Dose**	**Frequency**	**Indication**	**Start Date**
				__/__/_____
				__/__/_____
				__/__/_____
				__/__/_____
				__/__/_____
				__/__/_____
				__/__/_____
				__/__/_____
				__/__/_____
				__/__/_____

N.B. Please note current ongoing treatment should be documented under concomitant medications in REDCap

**Past Treatments for MS (DMT Only)**		
**Drug**	**Start Date**	**End Date**
	__/__/_____	__/__/_____
	__/__/_____	__/__/_____
	__/__/_____	__/__/_____
	__/__/_____	__/__/_____
	__/__/_____	__/__/_____
	__/__/_____	__/__/_____
	__/__/_____	__/__/_____
	__/__/_____	__/__/_____
	__/__/_____	__/__/_____
	__/__/_____	__/__/_____

Signed ____________________________________ Date __________________

Site #	____	____	____	____	Subj #	____	____	____	Initials	____	____	____

STOP-MS TRIAL

Child-bearing Potential and Contraception

Screening

(Females/Other only)

**Date of Assessment** (dd/mm/yyyy)	___/___/______
**Current Fertility Status?**	
**Premenopausal**	☐
**Perimenopausal**	☐
**Postmenopausal**	☐
**Other**	☐
**Status?** (if other)	____________________________
**FSH?** (if <45 years and postmenopausal)	_______ IU
**Date of Last Menstrual Period (LMP)?**	___/___/______

**Documented tubal ligation?**	Y ☐ N ☐	Year	_______
**Documented hysterectomy?**	Y ☐ N ☐	Year	_______
**Docum bilateral oophorectomy?**	Y ☐ N ☐	Year	_______

**Deemed incapable of conception by a gynaecologist or endocrinologist?**	Y ☐ N ☐
**Condition** (if yes)	____________________________________

**Contraception**<br>If the participant is postmenopausal (greater than 12 months since LMP if 45 years or more or greater than 12 months since last LMP and FSH >25 IU if under 45 years) or has had a documented tubal ligation, hysterectomy, bilateral oophorectomy or has been deemed incapable of bearing children by a gynaecologist of endocrinologist, then the following requirements for contraception do not apply. In all other circumstances the participant must be willing to adhere to one of the following methods of contraception for the duration of the trial.	
**Oral contraceptive pill (combined or mini)**	☐
**Contraceptive implant or slow release injection**	☐
**Intra-uterine device**	☐
**Other**	☐
**Type of contrapetion?** (if other)	_______________
All other forms of contraception (including vasectomy in the partner) are not acceptable forms of contraception and would not meet eligibility criteria.	

Signed ___________________________________ Date _________________

Site #	____	____	____	____	Subj #	____	____	____	Initials	____	____	____

STOP-MS TRIAL

Visit	

General Examination

**Date of Examination** (dd/mm/yyyy)	___/___/______
**Pulse** (bpm)	_____ bpm
**Blood Pressure** (mmHg)	_____/_____ mmHg
**Temperature** (°C)	_______ °C
**Weight**	_______ kg
**Cardiac**	Normal ☐ Abnormal ☐
**Respiratory**	Normal ☐ Abnormal ☐
**Abdomen**	Normal ☐ Abnormal ☐
**Lymph Nodes**	Normal ☐ Abnormal ☐
**Thyroid Gland**	Normal ☐ Abnormal ☐
**Skin**	Normal ☐ Abnormal ☐
**Joints**	Normal ☐ Abnormal ☐
**Details of Any Abnormal Findings**	
**Name of examiner**	____________________

Signed ________________________________ Date ________________

Site #	____	____	____	____	Subj #	____	____	____	Initials	____	____	____

STOP-MS TRIAL

Check for Gynaecomastia (males only)

Visit	

**Date of Examination** (dd/mm/yyyy)	___/___/______
**Breast enlargement noted by participant**	Yes ☐ No ☐
**Breast pain/tenderness noted by participant**	Yes ☐ No ☐
**Nipple discharge reported by participant**	Yes ☐ No ☐
**Firm breast disc noted on examination** (if present please report as AE)	Yes ☐ No ☐
**Side Affected** (Right, Left, Bilateral)	R ☐ L ☐ B ☐
**Any associated red flag signs** (hard, irregular, asymmetric, skin dimpling, nipple retraction, discharge, axillary lymphadenopathy)	

Signed ___________________________________ Date __________________

Site #	____	____	____	____	Subj #	____	____	____	Initials	____	____	____

STOP-MS TRIAL

MRI Brain Results

Visit	

Compared to last available MRI Brain

**Date of MRI** (dd/mm/yyyy)	___/___/______
**New T2 Lesion**	Yes ☐ No ☐
**Expanded T2 Lesion**	Yes ☐ No ☐
**New Gd-enhancing Lesion**	Yes ☐ No ☐
**Expanded Gd-enhancing Lesion**	Yes ☐ No ☐
**Any Coincidental Pathology**	Yes ☐ No ☐
**Details of Coincidental Pathology**	

Signed ____________________________________ Date __________________

Site #	____	____	____	____	Subj #	____	____	____	Initials	____	____	____

STOP-MS TRIAL

Screening

MS Diagnosis

**Date of First Symptoms** (dd/mm/yyyy)	___/___/______
**Total Number of Relapses**	_____
**Number of Relapses in Last 2 Years**	_____
**Number of Relapses in Last 1 Year**	_____
**Number of Clinical Sites**	_____

**MRI Brain**	Available ☐ Yes ☐ No
Date of Scan (dd/mm/yyyy)	___/___/______
Number of Lesions	_____
Periventricular Lesion	Yes ☐ No ☐
Juxtacortical Lesion	Yes ☐ No ☐
Dawson Finger Lesion	Yes ☐ No ☐
Inferior Temporal Lobe Lesion	Yes ☐ No ☐
Gd-enhancing Lesion	Yes ☐ No ☐ Not given ☐

**MRI Spine**	Available ☐ Yes ☐ No
Date of Scan (dd/mm/yyyy)	___/___/______
Number of Lesions	_____
Longitudinally Extensive Lesion	Yes ☐ No ☐
Gd-enhancing Lesion	Yes ☐ No ☐ Not given ☐

Signed ________________________________ Date ________________

**CSF Analysis**	Available ☐ Yes ☐ No
Date of LP (dd/mm/yyyy)	___/___/______
CSF Protein (mg/L)	_____ mg/L
CSF White Cell Count (x10^6/L)	_____ x10^6/L
CSF Oligoclonal Bands	Yes ☐ No ☐ Unk ☐
Serum Oligoclonal Bands	Yes ☐ No ☐ Unk ☐

**EBV Status**	
History of Glandular Fever	Yes ☐ No ☐
Age of Glandular Fever	_____ Years
EBV IgG Status	Pos ☐ Neg ☐ Unk ☐

**Vitamin D Status**	Available ☐ Yes ☐ No
Vitamin D Level	_____ nmol/L
Date of Test (dd/mm/yyyy)	___/___/______

**McDonald Criteria**	Met? Yes ☐ No ☐
≥ 2 clinical attacks and objective clinical evidence of ≥ 2 lesions	☐
≥ 2 clinical attacks and objective evidence of 1 lesion (DIS by attacks in different sites or MRI criteria)	☐
1 clinical attack and objective clinical evidence of ≥ 2 lesions (DIT by MRI criteria of OCB)	☐
1 clinical attack and objective evidence of 1 lesion (DIS by MRI criteria or DIT by MRI criteria or OCB)	☐

Signed ________________________________ Date ________________

**Disease Course**	
Primary Progressive (no evidence of prior relapses)	☐
Secondary Progressive (evidence of prior relapses)	☐
Relapsing-remitting	☐
Clinically Isolated Syndrome	☐
Radiologically Isolated Syndrome	☐

**Confirmation of PPMS Diagnosis**	
Duration of progression lasting 1 year or more	Yes ☐ No ☐
And	
1 year of disability progression (retrospectively or prospectively determined) independent of clinical relapse	Yes ☐ No ☐
Plus two of the following criteria	
One or more T2-hyperintense lesions characteristic of multiple sclerosis in one or more of the following brain regions: periventricular, cortical or juxtacortical, or infratentorial	Yes ☐ No ☐
Two or more T2-hyperintense lesions in the spinal cord	Yes ☐ No ☐
Presence of CSF-specific oligoclonal bands	Yes ☐ No ☐

Signed ______________________________ Date ______________

Site #	____	____	____	____	Subj #	____	____	____	Initials	____	____	____

**Evidence for Progressive MS**	
Documented worsening of EDSS score over the past 2 years*	☐
Documented worsening of a FS score over the past 2 years	☐
Documented worsening in walking tolerance	☐
Evidence of decline in function as determined by treating neurologist on history	☐
No evidence for progression[2]	☐

**Current MS Symptoms (even if intermittent)**	**Nature and location** (if none = "None")
**Visual**	
**Brainstem**	
**Motor (pyramidal)**	
**Motor (cerebellar)**	
**Sensory**	
**Bladder & bowel**	
**Cognitive**	

Signed ____________________________________ Date __________________

* Defined as an increase in EDSS of 1.0 or more if EDSS is between 0.0 and 4.5 and an increase in EDSS of 0.5 or more if EDSS is 5.0 or higher.

Site #	____	____	____	____	Subj #	____	____	____	Initials	____	____	____

STOP-MS TRIAL

Enrolment

Inclusion-exclusion Criteria

Inclusion Criteria	
**Aged 25 – 70 years (inclusive)**	Yes ☐ No ☐
**Diagnosed with PPMS or SPMS**	Yes ☐ No ☐
**Meets 2017 McDonald criteria**	Yes ☐ No ☐
**EDSS <4.0 – 8.0 (inclusive)**	Yes ☐ No ☐
**Evidence of disability progression over 2 years**	Yes ☐ No ☐
**Speaks English (or has translator)**	Yes ☐ No ☐
**Willing to attend scheduled visits**	Yes ☐ No ☐
**Meets all Inclusion criteria**	Yes ☐ No ☐

Exclusion Criteria	
**Clinical relapse within 3 months of enrolment**	Yes ☐ No ☐
**Relapsing remitting MS or clinically isolated syndrome**	Yes ☐ No ☐
**Any other serious comorbidity**	Yes ☐ No ☐
**Renal failure (eGFR < 30 ml/min/1.73m^2**	Yes ☐ No ☐
**Concomitant use of medication known to cause hyperkalaemia (see list of contraindicated medications)**	Yes ☐ No ☐
**Hypersensitivity to spironolactone**	Yes ☐ No ☐
**Hypersensitivity to famciclovir**	Yes ☐ No ☐
**Pregnant** (if female)	Yes ☐ No ☐
**Lactating** (if female)	Yes ☐ No ☐
**Unwilling to use contraception for the duration of the trial – up to 5 years (for**	Yes ☐ No ☐

**women of child-bearing age who are not incapable of child-bearing)**	
**Recent/current major depression, bipolar disorder, psychosis or suicidality**	Yes ☐ No ☐
**Recent/current illicit substance use**	Yes ☐ No ☐
**Have received IV/oral steroids in previous 3 months for MS relapse**	Yes ☐ No ☐
**Have received any trial therapy within the prior 6 months (or 4 weeks in the case of STOP-MS Stage 1)**	Yes ☐ No ☐
**Negative for all inclusion criteria**	Yes ☐ No ☐

**Participant Outcome**	
**Enrolled into STOP-MS**	Yes ☐ No ☐
**Randomised**	Yes ☐ No ☐
**Screen Failure**	Yes ☐ No ☐
**Reason for Screen Failure**	
**Does not meet inclusion criteria**	☐
**Has one or more exclusion criteria**	☐
**Contraindication to IMP/abnormal ECG**	☐
**Other**	☐
**Details**	

Site #	____	____	____	____	Subj #	____	____	____	Initials	____	____	____

**Participant Information**	
**Participant ID Card handed to participant**	Yes ☐ No ☐
**Participant List of Drugs and Supplements Causing Hyperkalaemia handed to participant**	Yes ☐ No ☐

Signed ____________________________________ Date __________________

Site #	____	____	____	____	Subj #	____	____	____	Initials	____	____	____

STOP-MS TRIAL

Visit	

ECG Report

**Date**	___/___/______
Heart Rate	______________ bpm
Rhythm	☐ Regular<br>☐ Regularly Irregular<br>☐ Irregularly Irregular
Axis	☐ Normal<br>☐ Right Deviation<br>☐ Left Deviation
P-waves	☐ Present<br>☐ Absent
PR Interval	_____________ ms
Conduction Block	☐ None<br>☐ First Degree<br>☐ Second Degree<br>☐ Type I<br>☐ Type II<br>☐ Third Degree
Delta Wave	☐ Absent<br>☐ Present
QRS Complex	☐ Narrow<br>☐ Broad<br>☐ Tall
Q-waves	☐ Normal<br>☐ Pathological
ST Segment	☐ Normal<br>☐ Elevation<br>☐ Depression
T waves	☐ Normal<br>☐ Tall

Signed ____________________________________ Date __________________

STOP-MS TRIAL

Enrolment

Pre-treatment Checklist

Investigation	Date	Result
Pathology		
FBC	___/___/______	☐ Normal ☐ Abnormal
Comment		
EUC	___/___/______	☐ Normal ☐ Abnormal
Comment		
LFTs	___/___/______	☐ Normal ☐ Abnormal
Comment		
eGFR	___/___/______	__________ ml/min/1.73m2
	>30ml/min/1.73m2	☐ Yes
	<30ml/min/1.73m2	☐ Yes
Comment		
bHCG	☐ Normal ☐ Abnormal ☐ N/A	
Comment		
Electrophysiology		
ECG	___/___/______	☐ Normal ☐ Abnormal
Comment		
Concomitant Medication		
Not taking medication known to cause hyperkalaemia		Yes ☐ No ☐
Trial ID Card Given to Participant		Yes ☐ No ☐
List of Contraindications Given to Participant		Yes ☐ No ☐
Comment		

Signed ____________________________________ Date __________________

Site #	____	____	____	____	Subj #	____	____	____	Initials	____	____	____

STOP-MS TRIAL

Visit	

Clinical Review

**Date of Review** (dd/mm/yyyy)	___/___/______
**New Neurological Symptoms Since Last Review?** (please provide details below)	Yes ☐ No ☐
**Relapse Since Last Review?**	Yes ☐ No ☐
If "Yes" please complete "On Study Relapse Details" form	
**Adverse Event Since Last Review?**	Yes ☐ No ☐
If "Yes" please complete "Adverse Event Details" form	
**New Medication Since Last Review?**	Yes ☐ No ☐
If "Yes" please complete "Concomitant Medications" form	
**Continuing Study Medication?**	Yes ☐ No ☐
If "No" please complete "Study Medication Dose Change" form	
**Withdrawing from Study?**	Yes ☐ No ☐
If "Yes" please complete "Withdrawal of Consent" form	
**End of Study?**	Yes ☐ No ☐
**Clinical Details**	

Signed ______________________________ Date _______________

Site #	____	____	____	____	Subj #	____	____	____	Initials	____	____	____

STOP-MS TRIAL

Visit	

Telephone Review

**Date of Review** (dd/mm/yyyy)	___/___/______
**New Neurological Symptoms Since Last Review?**	Yes ☐ No ☐
**Reports Significant Decline in Function?**	Yes ☐ No ☐
**Relapse Since Last Review?**	Yes ☐ No ☐
If "Yes" please complete "On Study Relapse Details"	
**Adverse Event Since Last Review?**	Yes ☐ No ☐
If "Yes" please complete "Adverse Event Details"	
**New Medication Since Last Review?**	Yes ☐ No ☐
If "Yes" please complete "Concomitant Medications"	
**Needs Unscheduled Review?**	Yes ☐ No ☐
If "Yes" please arrange and complete "Unscheduled Review"	
**Clinical Details**	

Signed ____________________________________ Date __________________

STOP-MS TRIAL

Visit	

Concomitant Medications

New Treatments						
**Drug**	**Dose**	**Units**	**Freq**	**Indication**	**Start Date**	**End Date**
					__/__/____	__/__/____
					__/__/____	__/__/____
					__/__/____	__/__/____
					__/__/____	__/__/____
					__/__/____	__/__/____
					__/__/____	__/__/____
					__/__/____	__/__/____
					__/__/____	__/__/____
					__/__/____	__/__/____
					__/__/____	__/__/____
					__/__/____	__/__/____
					__/__/____	__/__/____
					__/__/____	__/__/____
					__/__/____	__/__/____

Signed ____________________________________ Date __________________

Site #	____	____	____	____	Subj #	____	____	____	Initials	____	____	____

STOP-MS TRIAL

Visit	

Adverse Event Details

**Date of Event** (dd/mm/yyyy)	___/___/______
**Brief Description of Event**	
**Was This an MS Relapse**	Yes □ No □
If "Yes" please complete "On Study Relapse Details"	
**Severity of AE**	□ 1 = Mild<br>□ 2 = Moderate<br>□ 3 = Severe<br>□ 4 = Life threatening (SAE)<br>□ 5 = Death (SAE)
**Relationship to IMP**	□ 4 = Definitely<br>□ 3 = Probably<br>□ 2 = Possibly<br>□ 1 = Unrelated<br>□ 0 = Prior Condition
**Was This a Serious AE**	Yes □ No □
If "yes" to above report to HREC and sponsor (within 24 hours)	
**SAE Reported to Sponsor**	Yes □ No □
**Comment**	
**Resolution**	□ 0 = Unknown<br>□ 1 = Recovered completely<br>□ 2 = Recovering/resolving<br>□ 3 = Recovered with sequealae<br>□ 4 =Fatal
**Date of Resolution** (dd/mm/yyyy)	___/___/______

Signed ____________________________________ Date __________________

Site #	____	____	____	____	Subj #	____	____	____	Initials	____	____	____

STOP-MS TRIAL

Visit	

On Study Relapse Details

**Date of Review** (dd/mm/yyyy)		___/___/______
**Date of Symptom Onset** (dd/mm/yyyy)		___/___/______
**Clinical Details**		
**Systems Affected**		Vision ☐
Brainstem ☐	Pyramidal ☐	Sensory ☐
Cerebellar ☐	Bladder & Bowel ☐	Mood & Cognition ☐
**EDSS**		_____
**Has EDSS Deteriorated**		Yes ☐ No ☐
**New/Expanded Lesion on MRI**		Yes ☐ No ☐ Ukn ☐
**IVMP**		Yes ☐ No ☐
**Date of Resolution** (dd/mm/yyyy)		___/___/______
**Recovery**	Full ☐ Partial ☐ None ☐	

Signed ___________________________________ Date __________________

Site #	____	____	____	____	Subj #	____	____	____	Initials	____	____	____

Screening

STOP-MS TRIAL

Hospital Anxiety and Depression Scale (HADS)

Tick the box beside the reply that is closest to how you have been feeling in the past week.
Don't take too long over you replies: your immediate is best.

**I feel tense or 'wound up':**		**I feel as if I am slowed down:**	
Most of the time	☐	Nearly all the time	☐
A lot of the time	☐	Very often	☐
From time to time, occasionally	☐	Sometimes	☐
Not at all	☐	Not at all	☐
**I still enjoy the things I used to enjoy:**		**I get a sort of frightened feeling like 'butterflies' in the stomach:**	
Definitely as much	☐	Not at all	☐
Not quite so much	☐	Occasionally	☐
Only a little	☐	Quite Often	☐
Hardly at all	☐	Very Often	☐
			☐
**I get a sort of frightened feeling as if something awful is about to happen:**		**I have lost interest in my appearance:**	
Very definitely and quite badly	☐	Definitely	☐
Yes, but not too badly	☐	I don't take as much care as I should	☐
A little, but it doesn't worry me	☐	I may not take quite as much care	☐
Not at all	☐	I take just as much care as ever	☐
**I can laugh and see the funny side of things:**		**I feel restless as I have to be on the move:**	
As much as I always could	☐	Very much indeed	☐
Not quite so much now	☐	Quite a lot	☐
Definitely not so much now	☐	Not very much	☐
Not at all	☐	Not at all	☐
**Worrying thoughts go through my mind:**		**I look forward with enjoyment to things:**	
A great deal of the time	☐	As much as I ever did	☐
A lot of the time	☐	Rather less than I used to	☐
From time to time, but not too often	☐	Definitely less than I used to	☐
Only occasionally	☐	Hardly at all	☐
**I feel cheerful:**		**I get sudden feelings of panic:**	
Not at all	☐	Very often indeed	☐
Not often	☐	Quite often	☐
Sometimes	☐	Not very often	☐
Most of the time	☐	Not at all	☐
**I can sit at ease and feel relaxed:**		**I can enjoy a good book or radio or TV program:**	
Definitely	☐	Often	☐
Usually	☐	Sometimes	☐
Not Often	☐	Not often	☐
Not at all	☐	Very seldom	☐

Signed ____________________________________ Date __________________

STOP-MS TRIAL

Visit	

Neurological Examination

**Date of Examination** (dd/mm/yyyy)	___/___/______
**Visual Acuity**	Right _____ Left _____
**Scotoma**	Right ☐ Left ☐
**Colour Vision**	Right _____ Left _____
**Optic Atrophy**	Right ☐ Left ☐
**Brainstem**	Normal ☐ Abnormal ☐
**Details**<br>Nystagmus<br>EOM<br>Facial Movement<br>Speech<br>Bulbar	
**Pyramidal Function**	Normal ☐ Abnormal ☐
**Details**<br>Tone<br>Power<br>Reflexes<br>Plantars<br>Gait	
**Sensory Function**	Normal ☐ Abnormal ☐
**Details**<br>Light Touch<br>Temperature<br>Pin Prick<br>Vibration<br>Joint Position	

**Cerebellar**	Normal ☐ Abnormal ☐
**Details**<br>Limb<br>Trunk<br>Gait	
**Bladder and Bowel**	Normal ☐ Abnormal ☐
**Details**<br>Urgency<br>Frequency<br>Hesitancy<br>Incontinence<br>Catheter<br>Bowels<br>Sexual function	
**Mood and Cognition**	Normal ☐ Abnormal ☐
**Details**<br>Mood disturbance<br>Mild cognitive impairment<br>Moderate cognitive impairment<br>Severe cognitive impairment	
**Name of person who performed the neurological examination?**	________________________

Signed ___________________________________ Date _________________

Site #	____	____	____	____	Subj #	____	____	____	Initials	____	____	____

STOP-MS TRIAL

Visit	

EDSS

**Date of Assessment** (dd/mm/yyyy)	___/___/______
**Functional System**	**Score**
**Visual Function**	_____
**Brainstem Function**	_____
**Pyramidal Function**	_____
**Sensory Function**	_____
**Cerebellar Function**	_____
**Bladder and Bowel**	_____
**Mood and Cognition**	_____
**Ambulation Distance (m)**	_____
**Ambulation Score**	_____
**Overall EDSS**	_____
**Name of person who undertook this EDSS assessment?**	____________________________

Signed ____________________________________ Date __________________

STOP-MS TRIAL

Visit	

MSFC

**Date of Assessment** (dd/mm/yyyy)	___/___/______
**25-Foot Walk**	
**Did participant wear an AFO?**	Yes ☐ No ☐
**Was a walking aid used?**	Yes ☐ No ☐
**Unilateral**	☐
**Bilateral**	☐
**Trial 1**	
**Time for 25-Foot Walk** (seconds)	______ seconds
**Unable to complete**	☐
**Reason unable to complete**	____________________
**Trial 2**	
**Time for 25-Foot Walk** (seconds)	______ seconds
**Unable to complete**	☐
**Reason unable to complete**	____________________

**9-Hole Peg Test**			
**Date of Assessment** (dd/mm/yyyy)		___/___/______	
**Dominant Hand**		Right ☐ Left ☐	
**Overall EDSS**		____	
**Dominant Hand**		**Non-Dominant Hand**	
**Trial 1**		**Trial 1**	
**Time (s)**	_________ s	**Time (s)**	_________ s
**Unable to complete**	☐	**Unable to complete**	☐
**Comment**	______________	**Comment**	______________
**Trial 2**		**Trial 2**	
**Time (s)**	_________ s	**Time (s)**	_________ s
**Unable to complete**	☐	**Unable to complete**	☐
**Comment**	______________	**Comment**	______________
Did it take more than 2 attempts to get 2 successful trials?	Yes ☐ No ☐	Did it take more than 2 attempts to get 2 successful trials?	Yes ☐ No ☐

**SDMT**	
**Date of Assessment** (dd/mm/yyyy)	___/___/______
**Practice Set Completed**	Yes ☐ No ☐
**Number of Substitutions Completed in 90 Seconds**	________
**Number of Correct Substitutions in 90 Seconds**	________
**Unable to complete**	☐
**Reason**	________________

Signed ___________________________________ Date _________________

Site #	____	____	____	____	Subj #	____	____	____	Initials	____	____	____

STOP-MS TRIAL

MSIS-29 – PROM

- The following questions ask for your views about the impact of MS on your day-to-day life during the **past 2 weeks**
- For each statement, please **click** the **one** button that **best** describes your situation
- Please answer **all** questions

In the past two weeks, how much has your MS limited your ability to...		Not at all	A little	Moderately	Quite a bit	Extremely
1.	Do physically demanding tasks?	1	2	3	4	5
2.	Grip things tightly (e.g. turning on taps)?	1	2	3	4	5
3.	Carry things?	1	2	3	4	5

In the past two weeks, how much have you been bothered by...		Not at all	A little	Moderately	Quite a bit	Extremely
4.	Problems with your balance?	1	2	3	4	5
5.	Difficulties moving about indoors?	1	2	3	4	5
6.	Being clumsy?	1	2	3	4	5
7.	Stiffness?	1	2	3	4	5
8.	Heavy arms and/or legs?	1	2	3	4	5
9.	Tremor of your arms or legs?	1	2	3	4	5
10.	Spasms in your limbs?	1	2	3	4	5
11.	Your body not doing what you want it to do?	1	2	3	4	5
12.	Having to depend on others to do things for you?	1	2	3	4	5
Please check that you have answered all the questions before going on to the next page						
©2000 Neurological Outcome Measures Unit, 4th Floor Queen Mary Wing, NHNN, Queen Square, London WC1N 3BG, UK						

Signed ____________________________________ Date __________________

Site #	____	____	____	____	Subj #	____	____	____	Initials	____	____	____

In the past two weeks, how much have you been bothered by...		Not at all	A little	Moderately	Quite a bit	Extremely
13.	Limitations in your social and leisure activities at home?	1	2	3	4	5
14.	Being stuck at home more than you would like to be?	1	2	3	4	5
15.	Difficulties using your hands in everyday tasks?	1	2	3	4	5
16.	Having to cut down the amount of time you spent on work or other daily activities?	1	2	3	4	5
17.	Problems using transport (e.g. car, bus, train, taxi, etc.)?	1	2	3	4	5
18.	Taking longer to do things?	1	2	3	4	5
19.	Difficulty doing things spontaneously (e.g. going out on the spur of the moment)?	1	2	3	4	5
20.	Needing to go to the toilet urgently?	1	2	3	4	5
21.	Feeling unwell?	1	2	3	4	5
22.	Problems sleeping?	1	2	3	4	5
23.	Feeling mentally fatigued?	1	2	3	4	5
24.	Worries related to your MS?	1	2	3	4	5
25.	Feeling anxious or tense?	1	2	3	4	5
26.	Feeling irritable, impatient, or short tempered?	1	2	3	4	5
27.	Problems concentrating?	1	2	3	4	5
28.	Lack of confidence?	1	2	3	4	5
29.	Feeling depressed?	1	2	3	4	5
Please check that you have answered all the questions before going on to the next page						
©2000 Neurological Outcome Measures Unit, 4th Floor Queen Mary Wing, NHNN, Queen Square, London WC1N 3BG, UK						

Signed ____________________________________ Date __________________

Site #	____	____	____	____	Subj #	____	____	____	Initials	____	____	____

STOP-MS TRIAL

MSWS-12 – PROM

**Date** (dd/mm/yyyy)	___/___/______

Regarding the **past 2 weeks**, please click the button that best corresponds with how much your MS has…

	**Not at all**	**A little**	**Moderately**	**Quite a lot**	**Extremely**
1. Limited your ability to walk?	1	2	3	4	5
2. Limited your ability to run?	1	2	3	4	5
3. Limited your ability to climb up and down stairs?	1	2	3	4	5
4. Made standing when doing things difficult?	1	2	3	4	5
5. Limited your balance when standing or walking?	1	2	3	4	5
6. Limited how far you can walk?	1	2	3	4	5
7. Increased the effort needed for you to walk?	1	2	3	4	5
8. Made it necessary for you to use support when walking indoors (e.g. holding on to furniture, using a stick etc.)?	1	2	3	4	5
9. Made it necessary for you to use support when walking outdoors (e.g. using a stick, frame etc.)?	1	2	3	4	5
10 Slowed down your walking?	1	2	3	4	5
11. Affected how smoothly you walk?	1	2	3	4	5
12. Made you concentrate on your walking?	1	2	3	4	5

**Total Score**	______________

Signed ____________________________________ Date __________________

Site #	____	____	____	____	Subj #	____	____	____	Initials	____	____	____

STOP-MS TRIAL

Neuropathic Pain Scale – PROM

**Date** (dd/mm/yyyy)	___/___/______											
With regards to any pain that you experience please click the button that corresponds to the level of pain that you are **currently experiencing** on a scale from 0 = no pain through to 10 = the most severe pain imaginable.												
**Score**		**0**	**1**	**2**	**3**	**4**	**5**	**6**	**7**	**8**	**9**	**10**	
1. How intense is the pain?	No pain	☐	☐	☐	☐	☐	☐	☐	☐	☐	☐	☐	Most intense pain sensation imaginable
2. How sharp is the pain? 'Like a knife"	Not sharp	☐	☐	☐	☐	☐	☐	☐	☐	☐	☐	☐	Most sharp sensation imaginable
3. How hot is the pain? 'On fire'	Not hot	☐	☐	☐	☐	☐	☐	☐	☐	☐	☐	☐	Most hot sensation imaginable
4. How dull is the pain?	Not dull	☐	☐	☐	☐	☐	☐	☐	☐	☐	☐	☐	Most dull sensation imaginable
5. How cold is the pain? 'Freezing'	Not cold	☐	☐	☐	☐	☐	☐	☐	☐	☐	☐	☐	Most cold sensation imaginable
6. How sensitive is the skin to light touch? 'Raw skin'	Not sensit-ive	☐	☐	☐	☐	☐	☐	☐	☐	☐	☐	☐	Most sensitive pain sensation imaginable
7. How itchy is the pain?	Not itchy	☐	☐	☐	☐	☐	☐	☐	☐	☐	☐	☐	Most itchy sensation imaginable
8.How unpleasant is the pain? 'Intolerable'	Not unplea-sant	☐	☐	☐	☐	☐	☐	☐	☐	☐	☐	☐	The most unpleasant sensation imaginable
9. If the pain is deep, how intense is the deep pain?	No deep pain	☐	☐	☐	☐	☐	☐	☐	☐	☐	☐	☐	Most intense deep pain imaginable
10 If the pain is on the surface, how intense is the surface pain?	No surface pain	☐	☐	☐	☐	☐	☐	☐	☐	☐	☐	☐	Most intense surface pain imaginable

**What is the time quality of the pain?**	Background pain present all the time, with occasional flare ups (breakthrough pain)	☐
	Single type of pain present all the time	☐
	Single type of pain only sometimes present	☐
	I have no pain	☐
**Total Score**	___________	

Signed ___________________________________ Date _________________

Site #	____	____	____	____	Subj #	____	____	____	Initials	____	____	____

STOP-MS TRIAL

MFIS-21 – PROM

**Date** (dd/mm/yyyy)	___/___/______
Fatigue is a feeling of physical tiredness and lack of energy that many people experience from time to time. People who have medical conditions like MS experience stronger feelings of fatigue more often and with greater impact than others.<br>Following is a list of statements that describe the effects of fatigue. Please read each statement carefully, then click the button that best indicates how often fatigue has affected you in this way during the **past 4 weeks**. (If you need help in marking your responses, please ask a relative or carer to complete this task based on your responses.) Please answer every question. If you are not sure which answer to select, choose the one answer that comes closest to describing you.	

	**Never**	**Rarely**	**Sometimes**	**Often**	**Almost Always**
1. I have been less alert.	0	1	2	3	4
2. I have had difficulty paying attention for long periods of time.	0	1	2	3	4
3. I have been unable to think clearly.	0	1	2	3	4
4. I have been clumsy and uncoordinated.	0	1	2	3	4
5. I have been forgetful.	0	1	2	3	4
6. I have had to pace myself in my physical activities.	0	1	2	3	4
7. I have been less motivated to do anything that requires physical effort.	0	1	2	3	4
8. I have been less motivated to participate in social activities.	0	1	2	3	4
9. I have been limited in my ability to do things away from home.	0	1	2	3	4
10 I have trouble maintaining physical effort for long periods.	0	1	2	3	4
11. I have had difficulty making decisions.	0	1	2	3	4
12. I have been less motivated to do anything that requires thinking.	0	1	2	3	4
13. My muscles have felt weak.	0	1	2	3	4
14. I have been physically uncomfortable.	0	1	2	3	4
15. I have had trouble finishing tasks that require thinking.	0	1	2	3	4
16. I have had difficulty organizing my thoughts when doing things at home or at work.	0	1	2	3	4
17. I have been less able to complete tasks that require physical effort.	0	1	2	3	4

18. My thinking has been slowed down.	0	1	2	3	4
19. I have had trouble concentrating.	0	1	2	3	4
20. I have limited my physical activities.	0	1	2	3	4
21. I have needed to rest more often or for longer periods.	0	1	2	3	4

**Physical Subscale**	______________
**Cognitive Subscale**	______________
**Psychological Subscale**	______________
**Total MFIS Score**	______________

Signed ____________________________________ Date __________________

Instructions for Scoring the MFIS

Items on the MFIS can be aggregated into three subscales (physical, cognitive, and psychosocial), as well as into a total MFIS score. All items are scaled so that higher scores indicate a greater impact of fatigue on a person's activities.

Physical Subscale (range 0-36) = 4+6+7+10+13+14+17+20+21 (range 0-36)

Cognitive Subscale (range 0-40) = 1+2+3+5+11+12+15+16+18+19

Psychosocial Subscale (range 0-8) = 8+9

Total Score (range 0-84)

Site #	____	____	____	____	Subj #	____	____	____	Initials	____	____	____

STOP-MS TRIAL

EQ-5D-5L – PROM

Under each heading, please click the ONE box that best describes your health **TODAY.**

**MOBILITY**	
I have no problems in walking about	☐1
I have slight problems in walking about	☐2
I have moderate problems in walking about	☐3
I have severe problems in walking about	☐4
I am unable to walk about	☐5
**SELF-CARE**	
I have no problems washing or dressing myself	☐1
I have slight problems washing or dressing myself	☐2
I have moderate problems washing or dressing myself	☐3
I have severe problems washing or dressing myself	☐4
I am unable to wash or dress myself	☐5
**USUAL ACTIVITIES** (e.g. work, study, housework, family or leisure activities)	
I have no problems doing my usual activities	☐1
I have slight problems doing my usual activities	☐2
I have moderate problems doing my usual activities	☐3
I have severe problems doing my usual activities	☐4
I am unable to do my usual activities	☐5
**PAIN / DISCOMFORT**	
I have no pain or discomfort	☐1
I have slight pain or discomfort	☐2
I have moderate pain or discomfort	☐3
I have severe pain or discomfort	☐4
I have extreme pain or discomfort	☐5
**ANXIETY / DEPRESSION**	
I am not anxious or depressed	☐1
I am slightly anxious or depressed	☐2
I am moderately anxious or depressed	☐3
I am severely anxious or depressed	☐4
I am extremely anxious or depressed	☐5

Site #	____	____	____	____	Subj #	____	____	____	Initials	____	____	____

We would like to know how good or bad your health is TODAY.

This scale is numbered from 0 to 100.

100 means the best health you can imagine.

0 means the worst health you can imagine.

Mark an X on the scale to indicate how your health is **TODAY**

Now, please write the number you marked on the scale in the box below.

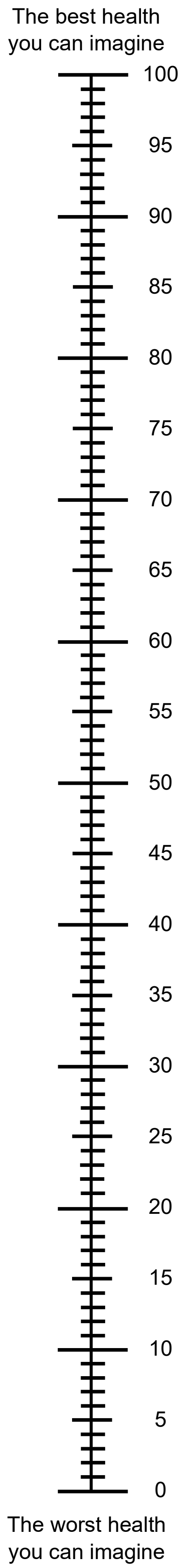

Your Health Today = [ ]

Signed ___________________________________ Date _________________

Site #	____	____	____	____	Subj #	____	____	____	Initials	____	____	____

STOP-MS TRIAL

Blood Results – FBC

Visit	

**Date of Testing** (dd/mm/yyyy)			___/___/______	
**FBC**	**Result**	**Units**	**LLN**	**ULN**
**Hb**	______	**g/L**	______	______
**WCC**	______	**x10^9/L**	______	______
**Neuts**	______	**x10^9/L**	______	______
**Lymphs**	______	**x10^9/L**	______	______
**Plts**	______	**x10^9/L**	______	______
**Clinical Significance (only required for abnormal results)** Please note only grade 1 abnormal results can be regarded as "not clinically significant".				
**Grade 1 Hb abnormality clinically significant?**			Yes ☐ No ☐	
**Grade 1 WCC abnormality clinically significant?**			Yes ☐ No ☐	
**Grade 1 Neuts abnormality clinically significant?**			Yes ☐ No ☐	
**Grade 1 Lymphs abnormality clinically significant?**			Yes ☐ No ☐	
**Grade 1 Plts abnormality clinically significant?**			Yes ☐ No ☐	
**Any Other Abnormality**				
**Grade 1 Other abnormality clinically significant?**			Yes ☐ No ☐	

Signed ________________________________ Date ________________

STOP-MS TRIAL

Blood Results – EUC & eGFR

Visit	

**Date of Testing** (dd/mm/yyyy)			___/___/______	
**EUC**	**Result**	**Units**	**LLN**	**ULN**
**Na**	______	**mmol/L**	______	______
**K**	______	**mmol/L**	______	______
**Urea**	______	**mmol/L**	______	______
**Cr**	______	**umol/L**	______	______
**eGFR**	______	**ml/min/1.73m^2**	______	
**Clinical Significance (only required for abnormal results)** Please note only grade 1 abnormal results can be regarded as "not clinically significant".				
**Grade 1 Na abnormality clinically significant?**			Yes ☐ No ☐	
**Grade 1 K abnormality clinically significant?**			Yes ☐ No ☐	
**Grade 1 Urea abnormality clinically significant?**			Yes ☐ No ☐	
**Grade 1 Cr abnormality clinically significant?**			Yes ☐ No ☐	
**Grade 1 eGFR abnormality clinically significant?**			Yes ☐ No ☐	
**Any Other Abnormality**				
**Grade 1 Other abnormality clinically significant?**			Yes ☐ No ☐	

Signed ______________________________ Date _______________

Site #	____	____	____	____	Subj #	____	____	____	Initials	____	____	____

STOP-MS TRIAL

Visit	

Blood Results – LFTs

**Date of Testing** (dd/mm/yyyy)			___/___/______	
**LFT**	**Result**	**Units**	**LLN**	**ULN**
**Tot Bili**	______	**umol/L**	______	______
**ALP**	______	**U/L**	______	______
**GGT**	______	**U/L**		______
**ALT**	______	**U/L**		______
**AST**	______	**U/L**		______
**Clinical Significance (only required for abnormal results)** Please note only grade 1 abnormal results can be regarded as "not clinically significant".				
**Grade 1 LFT abnormality clinically significant?**			Yes ☐ No ☐	
**Any Other Abnormality**				
**Grade 1 Other abnormality clinically significant?**			Yes ☐ No ☐	

Signed ___________________________________ Date __________________

Site #	____	____	____	____	Subj #	____	____	____	Initials	____	____	____

STOP-MS TRIAL

Visit	

Salivary EBV DNA Detection

**Week**	________________
**Date of Collection** (dd/mm/yyyy)	___/___/______
**Date of Testing** (dd/mm/yyyy)	___/___/______
**EBV DNA Detected**	Yes ☐ No ☐
**Titre**	___________

Signed ____________________________________ Date __________________

STOP-MS TRIAL

Visit	

EBNA1 Titre

**Week**	________________
**Date of Collection** (dd/mm/yyyy)	___/___/______
**Date of Testing** (dd/mm/yyyy)	___/___/______
**EBNA1 Antibodies Detected**	Yes ☐ No ☐
**EBNA1 Titre**	___________

Signed ____________________________________ Date __________________

Site #	____	____	____	____	Subj #	____	____	____	Initials	____	____	____

STOP-MS TRIAL

Pre-dose Escalation Checklist

Dose Escalation

Check week 3 blood results

**Investigation**	**Date ___/___/______**	**Result**
Potassium	Within normal range	☐ Yes ☐ No
eGFR	>30 ml/min	☐ Yes ☐ No
LFTs (ALP, AST, ALT, GGT)	Normal/Mildly Abnormal but Stable	☐ Yes<br>☐ No<br>☐ Mildly abnormal but stable (all <2xLLN)
Platelet Count	Within normal range	☐ Yes ☐ No
**Dose Escalation Decision** (Refer to protocol for guidance)		
Proceed to Full Dose		☐
Remain at Half Dose		☐
Withdraw Participant From Trial		☐

LLN = lower limit of normal

Signed ______________________________________ Date ___________________

Site #	____	____	____	____	Subj #	____	____	____	Initials	____	____	____

STOP-MS TRIAL

Study Medication Study Dose Change

Visit	

**Date of Dose Change** (dd/mm/yyyy)	___/___/______
Reason for Dose Change?	☐ Intolerance<br>(study medication related adverse events<br>☐ Toxicity<br>(new or worsening abnormal blood results)<br>☐ Contraindicated concomitant medication<br>☐ Serious adverse event<br>☐ Participant choice<br>☐ Other
Reason (if Other)	____________________________
Details	
**Dose Escalation Implemented**	
Changed to High Dose	☐
Changed to Half Dose	☐
Medication Ceased (Continuing with Study)	☐
Participant Withdrawn From Study	☐

Signed ___________________________________ Date _________________

Site #	____	____	____	____	Subj #	____	____	____	Initials	____	____	____

STOP-MS TRIAL

Pre-dose Escalation Checklist - Review

Dose Escalation

**Study Medication Dose Changed**	Yes ☐ No ☐
**Date of Dose Change** (dd/mm/yyyy)	___/___/______
Reason for Dose Change?	☐ Intolerance (study medication related adverse events<br>☐ Toxicity (new or worsening abnormal blood results)<br>☐ Contraindicated concomitant medication<br>☐ Serious adverse event<br>☐ Participant choice<br>☐ Other
Reason (if Other)	____________________________
Details	
**Dose Escalation Implemented**	
Changed to High Dose	☐
Changed to Half Dose	☐
Medication Ceased (Continuing with Study)	☐
Participant Withdrawn From Study	☐

Signed ____________________________________ Date __________________

STOP-MS TRIAL

Visit	

Pharmacy Details – Stage 1

**Date of Prescription** (dd/mm/yyyy)		___/___/______
**Randomisation**		
**Identification Checked**	☐	
**Randomisation Code**	___ ___ ___ ___	
**Spironolactone** ☐	**Famciclovir** ☐	**Placebo** ☐
**Half Dose** ☐	**Full Dose** ☐	
**Batch Number**		_____________
**Expiry Date** (dd/mm/yyyy)		___/___/______
**Drug**		_______________
**Dose** (mg)		______ mg
**No of Capsules** (n)		______
**Dispensed**		Yes ☐ No ☐

Signed ___________________________________ Date _________________

STOP-MS TRIAL

Pharmacy Details – Stage 2

Visit	

**Date of Prescription** (dd/mm/yyyy)	___/___/______
**Randomisation**	
**Identification Checked**	☐
**Randomisation Code**	___ ___ ___ ___
**Active Treatment** ☐*	**Placebo** ☐
**Half Dose** ☐	**Full Dose** ☐
**Batch Number**	_____________
**Expiry Date** (dd/mm/yyyy)	___/___/______
**Drug**	_______________
**Dose** (mg)	______ mg
**No of Capsules** (n)	______
**Dispensed**	Yes ☐ No ☐

Signed ___________________________________ Date _________________

* Only the successful IMP will proceed to Stage 2

STOP-MS TRIAL

Visit	

Dispensing Log

**Date of Dispensing** (dd/mm/yyyy)	___/___/______

Bottle	Batch/Lot Number	Tear-off Label
1	____________________	
2	____________________	
3	____________________	
4	____________________	
5	____________________	
6	____________________	

Site #	____	____	____	____	Subj #	____	____	____	Initials	____	____	____

Bottle	Batch/Lot Number	Affix Tear-off Label Here
7	________________	
8	________________	
9	________________	
10	________________	
11	________________	
12	________________	

Signed ____________________________________ Date __________________

STOP-MS TRIAL

Visit	

Compliance Check

**Date of Check** (dd/mm/yyyy)		___/___/______
**Bottle**	**Batch/Lot Number**	**Capsules Remaining**
**1**	____________________	_______________
**2**	____________________	_______________
**3**	____________________	_______________
**4**	____________________	_______________
**5**	____________________	_______________
**6**	____________________	_______________
**7**	____________________	_______________
**8**	____________________	_______________
**9**	____________________	_______________
**10**	____________________	_______________
**11**	____________________	_______________
**12**	____________________	_______________

Signed ______________________________________ Date ___________________

STOP-MS TRIAL

Visit	

Withdrawal of Consent

**Date of Withdrawal** (dd/mm/yyyy)	___/___/______
**Withdrawal of Consent form signed?**	Yes ☐ No ☐
**If not, Withdrawal documented in local case file/medical record?**	Yes ☐ No ☐
**Reason for Withdrawal (if offered)?**	
**Adverse event** (please ensure adverse event form completed)	☐
**Relapse** (please ensure relapse form completed)	☐
**Worsening of MS** (please ensure EDSS form completed – unscheduled visit)	☐
**Contraindicated concomitant medication** (please ensure concomitant medication form completed)	☐
**Change in personal circumstances** **(e.g. moving away, busy with work)**	☐
**Trial burden**	☐
**Other**	☐
**Details**	

Signed ___________________________________ Date _________________
