# Supplementary material for: Study protocol for a multicentre, randomised, double-blinded, placebo-controlled, multi-arm, multi-stage, trial of SpironolacTone and famciclOovir in the treatment of Progressive Multiple Sclerosis to prevent disability progression: the STOP-MS trial
Source: BMJ Neurol Open. 2025 Dec 23;7(2):e001313. doi: 10.1136/bmjno-2025-001313 (PMC12730750; doi:10.1136/bmjno-2025-001313)
Supplement: online supplemental file 6 [file bmjno-7-2-s006.pdf]

Phase III, multicentre, randomised, double-blinded, placebo-controlled, MAMS trial of SpironolacTone and famciclOvir in the treatment of Progressive MS to prevent disability progression (STOP-MS)

Statistical Analysis Plan

**Document History**		
Document	Date	Type of Amendment
Original (v1.0)	17/02/2025	Not Applicable

Approved By:

22/04/2025

Simon Broadley, Coordinating Principal Investigator — Date

23/04/2025

Tomas Kalincik, Chair Statistical Committee — Date

Terms & abbreviations

Abbreviation	Acronym Definition
6mCDP	6-month Confirmed Disability Progression
9-HPT	9-Hole Peg Test
AE	Adverse Event
AI	Associate Investigator
CD8	Cluster of Differentiation 8
CI	Chief Investigator
CNS	Central Nervous System
CONSORT	Consolidated Standards of Reporting Trials
CTN	Clinical Trial Notification
DMT	Disease Modifying Therapy
DNA	Deoxyribonucleic Acid
DSMB	Data Safety Monitoring Board
EBNA1	Epstein-Barr Nuclear Antigen-1
EBV	Epstein-Barr Virus
eCRF	Electronic Cade Report Form
EDSS	Expanded Disability Status Scale
eGFR	Estimated Glomerular Filtration Rate
EQ-5D-5L	EuroQol - 5 Domains – 5 Levels
EUC	Electrolytes, Urea and Creatinine
FBC	Full Blood Count
FIRMS-EBV	Fatigue In Relapsing Multiple Sclerosis – Epstein Barr Virus
FLAIR	Fluid Attenuated Inversion Recovery
FSMCF	Fatigue Scale Motor and Cognitive Functions
Gd	Gadolinium
HADS	Hospital Anxiety and Depression Scale
HLA	Human Leukocyte Antigen
HREC	Human Research Ethics Committee
ICH-GCP	International Conference on Harmonisation – Good Clinical Practice
IM	Infectious Mononucleosis
LFT	Liver Function Tests
LTFU	Lost To Follow Up
MAMS	Multi-Arm, Multi-Stage
MRI	Magnetic Resonance Imaging
MRFF	Medical Research Future Fund
MS	Multiple Sclerosis
MSFC	Multiple Sclerosis Functional Composite
MSIS-29	Multiple Sclerosis Impact Scale-29
MSWS-12	Multiple Sclerosis Walking Scale-12
NPS	Neuropathic Pain Scale
PCR	Polymerase Chain Reaction
PI	Principal Investigator
PICF	Participant Information and Consent Form

PIDN	Participant Identification Number
PLATYPUS	PLatform Adaptive Trial for remYelination & neuroprotection in MS
PROM	Participant Reported Outcome Measure
pwMS	Person with Multiple Sclerosis
QALY	Quality Adjusted Life Year
RCN	Randomisation Code Number
SAE	Serious Adverse Event
SDMT	Symbol Digit Modalities Test
SOC	Standard Of Care
SOP	Standard Operating Procedure
T25FW	Timed 25-Foot Walk
TNF	Tumour Necrosis Factor

Introduction & rationale

Multiple sclerosis (MS) is the commonest cause of neurological disability affecting young adults, with more than 33,000 Australians affected. It is a condition characterised by central nervous system (CNS) inflammation and neurodegeneration. Almost 50% of patients with MS will experience progressive form of the disease. Evidence of infection with Epstein-Barr virus (EBV) is essentially universal in people with MS, compared to being seen in 90% of the general adult population.

This project will harness the potential of removing or reducing EBV to treat progressive MS. Using a multiarm, multi-stage design (MAMS), it will compare the effect of spironolactone and famciclovir on confirmed disability progression in progressive MS.

Description of objectives & endpoints

Objective

Stage 1: to demonstrate that spironolactone or famciclovir plus SOC reduce the frequency of EBV DNA being present in saliva and/or reduce EBNA1 antibody titres in people with progressive MS when compared to placebo plus standard of care (SOC).

Stage 2: to demonstrate that spironolactone or famciclovir plus SOC reduce the likelihood of 6-month confirmed disability progression in people with progressive MS when compared to placebo plus SOC.

Secondary aims: To demonstrate that spironolactone or famciclovir plus standard of care:

a. are safe when used to treat people with progressive MS

b. reduce the rate of brain atrophy at 3 years compared to placebo plus SOC

c. reduce the numbers of new/expanded T2/ FLAIR and Gd-enhancing lesions on MRI brain compared to placebo plus SOC

d. reduce the level of whole brain atrophy on MRI brain compared to placebo plus SOC

e. improve patient-reported measures (PROMs) of disease impact compared to placebo plus SOC.

f. are cost-effective.

Primary endpoint:

Stage 1: composite outcome measures of salivary EBV DNA detection (viral shedding) and serum EBNA1 antibody titres.

Stage 2: time to 6mCDP using a composite of EDSS, T25FW and 9-HPT.

Secondary endpoints:

Clinical	- time to first relapse,<br>- time to 6mCDP using EDSS only<br>- mean change in EDSS<br><br>- MSFC Score<br>- SDMT<br><br>- MSReactor: log-transformed mean reaction times of the 3 tasks: simple reaction time task, choice reaction time task, one-back working memory task

MRI	- new and enlarging lesion counts
	- whole brain atrophy
PROMs	- MSIS-29
	- MSWS-12
	- Neuropathic Pain Scale
	- MFIS
Health economics	- EQ-5D-5L

Safety outcomes:

Adverse events (AEs) will be considered under the following categories: all treatment emergent AEs; treatment related AEs (definitely or probably related); higher grade AEs (grade 2 or higher); serious AEs and deaths. Once a minimum of 50 AEs have been reported (all classes of AE) event rates will be summarised according to treatment allocation (unblinded) for review by the data safety monitoring board (DSMB). Event rates will be compared between active treatment arms and placebo where more than 5 events of any class of AE have been seen.

Study design

Study Overview

A 24 – 240 week multicentre, randomised, double-blinded, placebo-controlled, MAMS phase III clinical trial of anti-EBV therapies as an add-on to SOC to assess outcomes related to EBV shedding, EBV serostatus and disability outcomes in progressive forms of MS.

Recruitment Procedure

Potential participants will be recruited in one of two ways. The first will be through approaches to people with MS being treated at the recruitment sites. The second will be via the MS Trial Screen online portal. The latter is an online web portal hosted by University of Tasmania and supported by MS Australia that allows people with MS to register their potential interest in one of three clinical trials (STOP-MS, PLATYPUS and FIRMS-EBV).

Inclusion Criteria

- Age 25-70 years (inclusive)
- Diagnosed with primary or secondary progressive MS according to McDonald 2017 criteria
- EDSS of 4.0 – 8.0 (inclusive) at the time of randomisation
- Evidence of disability progression over the previous 24 months
- English speaking or non-English speaking but can ensure external interpreter assistance (e.g. relative or friend) to attend all visits for the duration of the clinical trial
- Available to attend clinic visits

Exclusion Criteria

- A clinical relapse within 3 months of randomisation

- A significant co-morbidity that in the opinion of the principal investigator (PI) would negatively affect MS disease outcomes or preclude administration of spironolactone or famciclovir (including renal failure; estimated glomerular filtration rate < 30ml/min)
- Currently taking medication or supplements known to cause hyperkalaemia as listed in Appendix 16 of the Study Protocol
- Hypersensitivity to spironolactone or famciclovir
- Female participants who are pregnant
- Female participants who are breast-feeding
- Women of childbearing potential who are unwilling or unable to use an acceptable method of contraception (see Appendix 17 of the Study Protocol) whilst on trial treatment and for up to 30 days after the last dose of study drug
- Have received treatment with steroids (intravenous and/or oral) for MS relapse/progression within 3 months before randomisation
- Have received any trial therapy within the last 6 months (other than as part of the STOP-MS Stage 1 trial)
- Recent or current history of major depression, bipolar disorder, psychosis or suicidality
- Currently or recently taking any illicit substances (excluding cannabis products used for symptomatic relief)

Treatment allocation

At the enrolment visit, after eligibility is confirmed and the participant is to be enrolled, they will be randomised to one of the investigational medicinal products using the randomisation module of REDCap® which generates a randomisation code (4-digit number) from the "allocation table". The allocation table will be prepared in Excel®, Microsoft (Seattle, CA, USA) stratified by sex (three brackets), age (three brackets) and site. The randomisation codes will be assigned randomly, and the treatment allocations will be randomly assigned in blocks of 3 sorting using the RAND function of Excel®. In Stage 1, participants will be randomised at enrolment to treatment with spironolactone, famciclovir or placebo at a 1:1:1 ratio. The randomised treatment allocation will be displayed based on the treatment code in a REDCap® form only visible to site pharmacists. In Stage 2, participants will be randomised to either the successful treatment arm from stage 1 (spironolactone or famciclovir) or placebo in a 1:1 ratio. The same randomization process as above will be utilised but using a second randomisation schedule with just two treatment allocation options (treatment or placebo) and blocks of two. Randomisation procedures will all be performed by an unblinded monitor under the supervision of the unblinded statistician.

Stage 1:
Arm A: Standard of Care (SOC) plus Placebo
Arm B: SOC plus spironolactone
Arm C: SOC plus famciclovir

Stage 2:
Arms, whose therapies fail to meet the primary endpoint of Stage 1, will be terminated. Participants enrolled in these arms will have the option to be re-randomised into the remaining arms, following a 1-week washout period.

Scenario 2A: One treatment arm fails to meet the primary endpoints of Stage 1 and one treatment arm meets the primary endpoints of Stage 1: One treatment arm will be terminated and one treatment arm will be retained in Stage 2. The participants in the terminated arm will be re-randomised into the remaining two arms and their study time will be reset to time 0 (baseline) at the start of Stage 2. Subsequent analyses will be adjusted for the number of randomisations [1, 2].
Scenario 2B: Both treatment arms fail to meet the primary endpoint of Stage 1: Both treatment arms will be terminated and new treatment arm or arms will be added, commencing Stage 1.
Scenario 2C: Both treatment arms meet the primary endpoints of Stage 1: Both treatment arms will be retained in Stage 2. Study time will not be reset; the study baseline will remain at the start of Stage 1 for all participants.

Figure 1. Summary of Trial Design

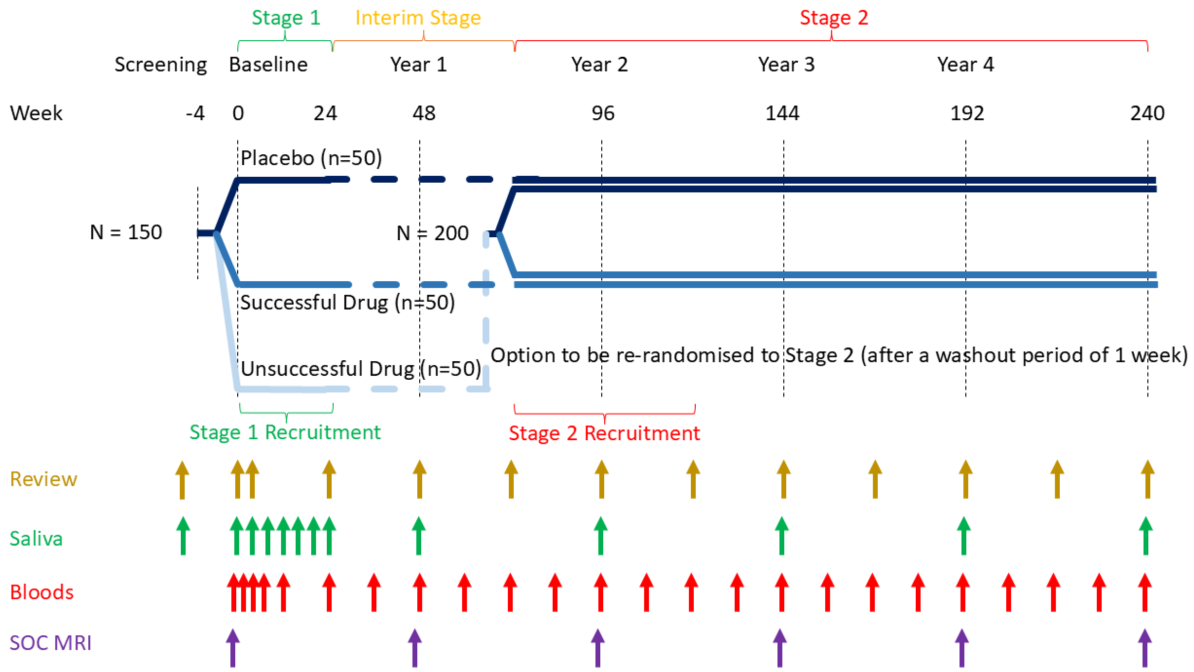

SOC MRI = Standard of Care Magnetic Resonance Imaging

Discontinuation and censoring

Patients will be assigned to treatment groups on an intention-to-treat basis. Participants who cease investigational medicinal product for any reason will be encouraged to remain in the study and complete all review assessments wherever possible. All such data will be included in the analysis. Where a participant withdraws completely from the study, outcome data will be censored at the point in time they left the study.

Assessments and variables

Demographics, MS history, and medical/neurological exam

Participants will be required to undergo a screening visit prior to enrolment (-4 to -1 weeks) to provide written informed consent and undertake comprehensive review of medical history

including; demographic parameters, details of MS history, other past medical and surgical history, family history, social history and allergies, review of past and current medications and health supplements, comprehensive medical and neurological examination including EDSS score, and Hospital Anxiety and Depression Scale (HADS).

Outcome measures

Primary endpoints:

Stage 1:

Proportion of patients with the composite primary end point of:

- EBV DNA present in saliva at 4 time points - in weeks 12, 16, 20 or 24,

OR

- increase/no change in EBNA1 IgG titre in serum at month 6 post-baseline compared to the study baseline.

Arm termination rule:

A treatment arm will be terminated at the end of Stage 1 if the absolute difference in the proportions of patients meeting the composite primary endpoint does not favour the intervention over placebo by at least 10% of the participants within the arm with a 95% confidence.

Stage 2:

The ratio of cumulative hazards of 6mCDP using a composite of EDSS, T25FW and 9-HPT over a period of 240 weeks.

Secondary endpoints:

(Study periods as defined under Primary endpoints, Stage 2):

The ratio of cumulative hazards of experiencing first relapse.

The ratio of cumulative hazards of 6mCDP using EDSS only.

Mean change in EDSS and MSFC scores.

Cumulative number of new or enlarging lesion during the study period.

Patient reported outcomes: Mean change in MSIS-29, MSWS-12, Neuropathic Pain Scale, FSMCF, recorded every 48 weeks during the study period

Economic Impact: Change in EQ-5D-5L over time using both the overall impact score (visual analogue scale) and the 5-digit health state data. Economic impact will be assessed using utility values derived from Australia specific value sets.

Study participants and power

Stage 1

EBV DNA is expected to be detected in 48% of the participants in the placebo arm. Based on a placebo-controlled RCT of teriflunomide, the proportion of patients in whom EBNA1 IgG titre will decrease is expected to be 35-55%. Thus, the number of participants reaching the composite endpoint at the end of Stage 1 is predicted to range from 50% to 70%. An absolute reduction in this rate of 10% would be considered as clinically meaningful.

Stage 2

Sample size calculations are based on α=0.05. 6mCDP is expected to be detected in 40% of the participants in the placebo arm. Based on the placebo-controlled ORATORIO trial, a sample size of 132 participants in each arm will provide 80% power to detect a 40% relative reduction in 6mCDP to 24% of participants in the active treatment arms. Accounting for a 14% drop-out rate, 150 participants will be required in each arm.

We aim to recruit 50 participants into each of the 3 arms in Stage 1. After completion of the Stage 1 and re-randomisation of one of the treatment arms, we will recruit and additional 100 participants into each of the arms regained in Stage 2.

Data handling, setup, and analysis

For the purpose of the analysis outlined in this Plan, data will be stored electronically on a secured password protected eCRF created using REDCap® hosted on a Griffith University server. Analyses will be conducted using R, STATA/SE or SAS statistical packages, as appropriate. Variables will be uniformly coded and labelled, with reference to the primary data sources and study databases.

Missing data

Missing values will be imputed using multiple imputation. Constant variables with information available at the minimum of one timepoint will be allowed to carry-over. A sensitivity analysis examining the validity of the missingness-at-random assumption with a pattern-mixture model will be carried out.

Data quality assurance

Data will be reviewed to ensure values are within their respective plausible ranges, both in general and relative to other measures taken during the study (between timepoints and between related variables). Departures from the plausible will be recorded and potential corrections made, conservatively, such that presumed typographical errors or errors in units may be corrected. In the absence of potential correction, values will be obtained through primary source verification or will be coded as missing.

Statistical methods

Deviations from the inclusion criteria will be handled in consultation with the CPI.

Results from Stage 1 will be evaluated for retention of treatment arms into Stage 2.

Hypotheses will be tested at α≤0.05. In accordance with the US Food and Drug Admnistration guidance on platform trials, Type I error will not be controlled across treatment arms. The comparisons are pairwise against the control study arm. Appropriate point (mean) and interval estimates (95% confidence interval) will be calculated for the associations of interest for the primary, secondary and exploratory analyses.

Treatment arm allocations will be modelled as fixed terms. In mixed-effect models, multiple entries will be included for repeated measures of the outcomes over time, with patient ID included as random intercept.

Definition of Baseline

Study baseline is defined as the date of receiving the first dose of the study therapy or placebo. In Stage 1, this is the date of administration of the study therapy as per randomisation. Participants from discontinued treatment arms, re-randomised in Stage 2, will be given a new baseline at the time of administration of the Stage 2 study therapy, as per re-randomisation. The outcomes will be compared using the intention-to-treat causal contrast. Balance of the compared treatment arms on patient demographic, clinical and paraclinical characteristics at baseline will be confirmed with standardised mean differences between the treatment arms.

Primary analysis

Stage 1

The decision to terminate a treatment/retain a treatment arm will be made based on the clinically meaningful effect size, as defined above. Stratified absolute risk differences and respective 95% confidence intervals will be estimated using the Mantel-Haenszel (MH) risk difference estimator that calculates a weighted average of stratum-specific risk difference estimators across sex and age strata as defined for randomization.

Where the confidence that the difference in the proportion of patients with reduced EBV DNA shedding or EBNA1 IgG does not reach/exceed the value of the clinically meaningful effect size (favouring the treatment group) is greater than 95%, futility of the corresponding treatment arm will be accepted and the arm will be terminated.

Stage 2

Cox proportional hazards model will be used to compare cumulative hazards of 6mCDP among the treatment arms. The proportionality-of-hazards assumption will be evaluated using inspection of Schoenfeld residuals and global test. Participants will be included from the time of their most recent randomisation (baseline). The number of randomisations [with values {1, 2}] will be modelled as a fixed term. Individual records will be right-censored at the time of reaching the outcome of interest or at the last recorded study time-point, whichever occurs first.

Secondary analyses

Similar to the models described above, secondary outcomes (Stage 2) will be analysed with fixed or mixed-effect, linear, ordinal or logistic regression models, as appropriate.

Sensitivity analyses

Primary analysis will be repeated with per-protocol causal contrast.

Analysis of safety outcomes

The frequencies of AEs and the proportions of patients with AEs will be reported – for the overall cohort, stratified by treatment arm and by AE severity and likely association with study intervention. AEs with ≥20% incidence will be highlighted. Continuous laboratory measures will be reported as mean and standard deviation, or median and quartiles, as appropriate. Volcano plot for AEs in the two treatment arms will be reported.

The proportions of patients experiencing specific AEs of interest will be compared with Fisher's exact test. The frequencies of AEs of interest will be compared between treatment arms using a negative binomial model.
